# Febuxostat does not delay progression of carotid atherosclerosis in patients with asymptomatic hyperuricemia: A randomized, controlled trial

**DOI:** 10.1371/journal.pmed.1003095

**Published:** 2020-04-22

**Authors:** Atsushi Tanaka, Isao Taguchi, Hiroki Teragawa, Nobukazu Ishizaka, Yumiko Kanzaki, Hirofumi Tomiyama, Masataka Sata, Akira Sezai, Kazuo Eguchi, Toru Kato, Shigeru Toyoda, Ryoichi Ishibashi, Kazuomi Kario, Tomoko Ishizu, Shinichiro Ueda, Koji Maemura, Yukihito Higashi, Hirotsugu Yamada, Mitsuru Ohishi, Kotaro Yokote, Toyoaki Murohara, Jun-ichi Oyama, Koichi Node, on behalf of the PRIZE study investigators

**Affiliations:** 1 Department of Cardiovascular Medicine, Saga University, Saga, Japan; 2 Department of Cardiology, Dokkyo Medical University Saitama Medical Center, Koshigaya, Japan; 3 Department of Cardiovascular Medicine, JR Hiroshima Hospital, Hiroshima, Japan; 4 Department of Cardiology, Osaka Medical College, Takatsuki, Japan; 5 Department of Cardiology, Tokyo Medical University, Tokyo, Japan; 6 Department of Cardiovascular Medicine, Institute of Biomedical Sciences, Tokushima University Graduate School, Tokushima, Japan; 7 The Department of Cardiovascular Surgery, Nihon University School of Medicine, Tokyo, Japan; 8 Department of Internal Medicine, Hanyu General Hospital, Hanyu, Japan; 9 Department of Clinical Research, National Hospital Organization, Tochigi Medical Center, Utsunomiya, Japan; 10 Department of Cardiovascular Medicine, Dokkyo Medical University, Mibu, Japan; 11 Division of Diabetes, Endocrinology and Metabolism, Department of Medicine, Kimitsu Chuo Hospital, Kisarazu, Japan; 12 Division of Cardiovascular Medicine, Department of Medicine, Jichi Medical University School of Medicine, Shimotsuke, Japan; 13 Department of Clinical Laboratory Medicine, Faculty of Medicine, University of Tsukuba, Tsukuba, Japan; 14 Department of Clinical Pharmacology and Therapeutics, University of the Ryukyus, Nishihara, Japan; 15 Department of Cardiovascular Medicine, Nagasaki University Graduate School of Biomedical Sciences, Nagasaki, Japan; 16 Department of Cardiovascular Regeneration and Medicine, Research Institute for Radiation Biology and Medicine, Hiroshima University, Hiroshima, Japan; 17 Department of Cardiovascular Medical and Hypertension, Graduate School of Medical and Dental Sciences, Kagoshima University, Kagoshima, Japan; 18 Department of Endocrinology, Hematology and Gerontology, Chiba University Graduate School of Medicine, Chiba, Japan; 19 Department of Cardiology, Nagoya University Graduate School of Medicine, Nagoya, Japan; University of Bologna, ITALY

## Abstract

**Background:**

An elevated level of serum uric acid (SUA) is associated with an increased risk of cardiovascular disease. Pharmacological intervention with urate-lowering agents, such as the conventional purine analogue xanthine oxidase (XO) inhibitor, allopurinol, has been used widely for a long period of time in clinical practice to reduce SUA levels. Febuxostat, a novel non-purine selective inhibitor of XO, has higher potency for inhibition of XO activity and greater urate-lowering efficacy than conventional allopurinol. However, clinical evidence regarding the effects of febuxostat on atherosclerosis is lacking. The purpose of the study was to test whether treatment with febuxostat delays carotid intima-media thickness (IMT) progression in patients with asymptomatic hyperuricemia.

**Methods and findings:**

The study was a multicenter, prospective, randomized, open-label, blinded-endpoint clinical trial undertaken at 48 sites throughout Japan between May 2014 and August 2018. Adults with both asymptomatic hyperuricemia (SUA >7.0 mg/dL) and maximum IMT of the common carotid artery (CCA) ≥1.1 mm at screening were allocated equally using a central web system to receive either dose-titrated febuxostat (10–60 mg daily) or as a control-arm, non-pharmacological lifestyle modification for hyperuricemia, such as a healthy diet and exercise therapy. Of the 514 enrolled participants, 31 were excluded from the analysis, with the remaining 483 people (mean age 69.1 years [standard deviation 10.4 years], female 19.7%) included in the primary analysis (febuxostat group, 239; control group, 244), based on a modified intention-to-treat principal. The carotid IMT images were recorded by a single sonographer at each site and read in a treatment-blinded manner by a single analyzer at a central core laboratory. The primary endpoint was the percentage change from baseline to 24 months in mean IMT of the CCA, determined by analysis of covariance using the allocation adjustment factors (age, gender, history of type 2 diabetes, baseline SUA, and baseline maximum IMT of the CCA) as the covariates. Key secondary endpoints included changes in other carotid ultrasonographic parameters and SUA and the incidence of clinical events. The mean values (± standard deviation) of CCA-IMT were 0.825 mm ± 0.173 mm in the febuxostat group and 0.832 mm ± 0.175 mm in the control group (mean between-group difference [febuxostat − control], −0.007 mm [95% confidence interval (CI) −0.039 mm to 0.024 mm; *P* = 0.65]) at baseline; 0.832 mm ± 0.182 mm in the febuxostat group and 0.848 mm ± 0.176 mm in the control group (mean between-group difference, −0.016 mm [95% CI −0.051 mm to 0.019 mm; *P* = 0.37]) at 24 months. Compared with the control group, febuxostat had no significant effect on the primary endpoint (mean percentage change 1.2% [95% CI −0.6% to 3.0%] in the febuxostat group (*n* = 207) versus 1.4% [95% CI −0.5% to 3.3%] in the control group (*n* = 193); mean between-group difference, −0.2% [95% CI −2.3% to 1.9%; *P* = 0.83]). Febuxostat also had no effect on the other carotid ultrasonographic parameters. The mean baseline values of SUA were comparable between the two groups (febuxostat, 7.76 mg/dL ± 0.98 mg/dL versus control, 7.73 mg/dL ± 1.04 mg/dL; mean between-group difference, 0.03 mg/dL [95% CI −0.15 mg/dL to 0.21 mg/dL; *P* = 0.75]). The mean value of SUA at 24 months was significantly lower in the febuxostat group than in the control group (febuxostat, 4.66 mg/dL ± 1.27 mg/dL versus control, 7.28 mg/dL ± 1.27 mg/dL; mean between-group difference, −2.62 mg/dL [95% CI −2.86 mg/dL to −2.38 mg/dL; *P* < 0.001]). Episodes of gout arthritis occurred only in the control group (4 patients [1.6%]). There were three deaths in the febuxostat group and seven in the control group during follow-up. A limitation of the study was the study design, as it was not a placebo-controlled trial, had a relatively small sample size and a short intervention period, and only enrolled Japanese patients with asymptomatic hyperuricemia.

**Conclusions:**

In Japanese patients with asymptomatic hyperuricemia, 24 months of febuxostat treatment did not delay carotid atherosclerosis progression, compared with non-pharmacological care. These findings do not support the use of febuxostat for delaying carotid atherosclerosis in this population.

**Trial registration:**

University Hospital Medical Information Network Clinical Trial Registry UMIN000012911.

## Abstract 2

In a randomized, controlled trial, Atsushi Tanaka and colleagues test whether treatment with febuxostat delays carotid intima-media thickness (IMT) progression in patients with asymptomatic hyperuricemia.

## Author summary

**Why was this study done?:**

- Elevated levels of serum uric acid (SUA) increase the risk of gout and may be associated with the risk of developing cardiometabolic disorders and subsequent cardiovascular disease.
- Translational research suggests that urate-lowering therapy by inhibition of xanthine oxidase (XO) may attenuate experimental atherosclerosis. However, the clinical effects of XO inhibitors on atherosclerosis in patients with asymptomatic hyperuricemia remain uncertain.
- Carotid intima-media thickness (IMT) measurement is a simple and noninvasive method to monitor atherosclerosis. We therefore investigated whether treatment with febuxostat, a non-purine selective inhibitor of XO, delayed the progression of carotid atherosclerosis compared to non-pharmacological care for hyperuricemia in this patient population.

**What did the researchers do and find?:**

- The study was a multicenter, prospective, randomized, open-label, blinded-endpoint clinical trial on 483 eligible participants (mean age 69.1 [standard deviation 10.4], female 19.7%) with asymptomatic hyperuricemia (SUA >7.0 mg/dL) and maximum IMT of the common carotid artery ≥1.1 mm. The results showed that there was no significant difference between febuxostat and non-pharmacological care for hyperuricemia in the progression of carotid atherosclerosis assessed by IMT over 24 months of follow-up.
- Febuxostat treatment was well tolerated and did not increase the incidence of cardiovascular and all-cause mortality.

**What do these findings mean?:**

- Our findings do not support the use of febuxostat to delay carotid atherosclerosis in patients with asymptomatic hyperuricemia.
- Further research is needed to determine the effects of febuxostat on atherosclerosis and cardiovascular safety in patients with hyperuricemia irrespective of the presence of gout.

## Introduction

An elevated level of serum uric acid (SUA) may be associated with cardiometabolic abnormalities, including hypertension and insulin resistance, potentially leading to the development of atherosclerosis and resultant cardiovascular disease (CVD) [1–3]. Thus, hyperuricemia is considered a residual risk factor for CVD [4], and whether urate-lowering therapy can attenuate atherosclerosis and improve prognosis is an important clinical issue.

Pharmacological intervention with urate-lowering agents, such as allopurinol, a conventional purine analogue xanthine oxidase (XO) inhibitor, has been used widely for a long period of time in clinical practice to prevent the development of gout and reduce SUA levels. XO is an enzyme responsible for catalyzing the final step in the conversion of purine to uric acid. Its action produces excess reactive oxygen species that can play a detrimental role in the pathogenesis of atherosclerosis [5,6]. To date, experimental studies in apolipoprotein E knockout mice fed a Western-type diet [7,8] have demonstrated that XO inhibition improved endothelial function and attenuated experimental atherosclerosis by suppressing excess production of oxidative stress.

Febuxostat, a novel non-purine selective inhibitor of XO, has been approved for the treatment of hyperuricemia and gout. Febuxostat is known to have greater potency for inhibition of XO activity and more urate-lowering efficacy than conventional allopurinol [9,10]. Accordingly, febuxostat may have superior anti-oxidative and anti-atherogenic effects to allopurinol [7,11]. However, in the recent CARES trial that enrolled patients with gout and documented CVD, febuxostat was noninferior to allopurinol with respect to the incidence of composite CV events, including atherosclerosis-related coronary and cerebral events [12]. Thus, it is still controversial whether febuxostat can exert a beneficial impact on atherosclerosis and relevant CVD in clinical settings [13].

We herein report the findings of a long-term, multicenter, randomized clinical study that investigated the effects of febuxostat on atherosclerosis in Japanese patients with hyperuricemia by measuring changes in carotid intima-media thickness (IMT) as a surrogate marker for atherosclerosis.

## Methods

### Study design

The PRIZE (program of vascular evaluation under uric acid control by xanthine oxidase inhibitor, febuxostat: multicenter, randomized controlled) study was a multicenter, prospective, randomized, open-label, blinded-endpoint clinical trial. After trial registration was completed in January 2014 (University Hospital Medical Information Network Clinical Trial Registry UMIN000012911 [https://upload.umin.ac.jp/cgi-open-bin/ctr_e/ctr_view.cgi?recptno=R000015081]), trial recruitment was carried out between May 2014 and August 2018 at 48 clinical sites throughout Japan.

The study protocol was approved by the local institutional review boards and independent ethics committees at all sites. The trial was conducted in full compliance with the Declaration of Helsinki and according to the Ethical Guidelines for Medical and Health Research Involving Human Subjects established by the Ministry of Health, Labour, and Welfare and Ministry of Education, Culture, Sports, Science, and Technology in Japan. Prior to enrollment, all participants received an adequate explanation of the study plan and provided written informed consent. The study is reported according to the CONSORT guidelines (**S1 CONSORT Checklist**).

The maximum IMT of the common carotid artery (CCA) was measured prior to randomization to assess eligibility. After eligibility was confirmed and the medical background reviewed, the patients were randomized equally to either a febuxostat group (10–60 mg daily) or a control group (non-pharmacological treatment of hyperuricemia). All patients were followed up with study visits scheduled at 1, 2, 3, 6, 12, and 24 months after the baseline visit. Carotid artery ultrasonography was performed at baseline and after 12 and 24 months (or at premature termination) at each local site. The full protocol appears in **S1 Text**, and the details of the study rationale and design have been described previously [14].

### Study participants

The inclusion and exclusion criteria for the study are listed in **S1 Table**. Individuals eligible for the study were adults (aged ≥20 years) who had asymptomatic hyperuricemia with an SUA >7.0 mg/dL and a maximum CCA-IMT ≥1.1 mm measured at eligibility assessment, defined as a carotid arterial plaque (localized protruding lesion) in accordance with the guidelines of the Japan Society of Ultrasonic in Medicine and the Japan Academy of Neurosonology [15]. Key exclusion criteria were the administration of any SUA-lowering agents within the 8-week period prior to assessment of eligibility, the presence of gouty tophus, or symptoms of gout arthritis within one year before assessment of eligibility.

### Randomization and treatment

Patients were randomized to a febuxostat group or control group in a 1:1 ratio at the automatic web-based PRIZE Data Center. The randomization was performed using a modified minimization method with a biased-coin assignment balanced for age (<65, ≥65 years), gender, presence or absence of type 2 diabetes, SUA (<8.0, ≥8.0 mg/dL), and maximum CCA-IMT (<1.3, ≥1.3 mm).

All participants in both groups received an appropriate lifestyle modification for hyperuricemia, such as healthy diet and exercise therapy, and the modification was continued during the study period. Based on the protocol, patients assigned to the febuxostat group received an initial dose of 10 mg daily that was titrated to 20 mg daily at 1 month and 40 mg daily at 2 months. Febuxostat 40 mg daily was targeted as the maintenance dose, but at 3 months or later, the dose of febuxostat could be increased to 60 mg daily. If SUA levels decreased to ≤2.0 mg/dL during the study period, the maintenance dose was decreased by 20 mg.

Each participant’s background treatment, such as antidiabetic agents, antiplatelet agents, antihypertensive agents, and lipid-lowering agents, was unchanged during the study period if possible, based on the patient’s clinical condition.

### Study endpoints

The primary endpoint of the study was the percentage change in the mean IMT of the CCA from baseline to 24 months.

The secondary endpoints included the following: (1) The values and changes from baseline at 12 and 24 months in the mean and maximum IMT of the CCA (except for the primary endpoint), bulb, and the internal carotid artery (ICA), and the plaque area and plaque grayscale median; (2) the values and changes from baseline in SUA at 6, 12, and 24 months; (3) the values and changes from baseline in vital signs and laboratory measurements; (4) the incidence of prespecified composite and individual clinical events (**S2 Text**), consisting of cardiovascular death, nonfatal myocardial infarction and stroke, renal events (doubling of serum creatinine, initiation of renal replacement therapy, or renal transplantation), and all-cause death; and (5) adverse events during the study period.

### Measurement of carotid IMT

The protocol and method for measuring carotid IMT have been described in detail previously [14,16]. According to the testing manual (**S3 Text**), based on the standardized protocol [17], high-resolution carotid ultrasonography using a standard system equipped with a >7.5-MHz linear transducer was performed at each site in a blinded manner by an expert, trained sonographer who had attended a lecture on acquiring images of the carotid IMT. All the imaging data were stored as JPEG files and then sent to the core imaging laboratory, where an expert analyzer measured the IMT values in a blinded manner using an automated IMT measurement software program (Vascular Research Tools 5, Medical Imaging Applications LLC, Coralville, IA) [18].

To measure mean CCA-IMT, longitudinal B-mode images of each CCA were obtained, and mean IMT of the CCA was determined at the continuous region 10 mm proximal to the origin of the bulb at the far wall using an auto-tracing system. Maximum CCA-IMT was defined as the maximum IMT measured at the near and far walls in the scanned CCA areas. The mean and maximum IMT values on the left and right sides were averaged at baseline and at 12 and 24 months. The absolute and percentage changes from baseline at 12 and 24 months were calculated for each side and then averaged. The following IMTs were also measured in a similar manner: mean and maximum IMTs of the bulb and ICA. Plaque was defined as a focal region with an IMT ≥1.1 mm that protruded into the lumen and was distinct from the adjacent boundary [15], and the plaque area with the lowest echogenicity and its median gray scale were also measured automatically [19].

### Power calculation

The sample size was calculated based on the following assumptions: a 1.6% between-group difference in the primary endpoint 24 months after baseline [16,20], a standard deviation (SD) of 6.0% for individual differences, and 80% detection power for a two-tailed, two-sample *t* test at a significance level of 0.05. The sample size was set at 250 patients in each group, with anticipation of a 10% dropout rate.

### Statistical analysis

The full statistical analysis plan appears in **S4 Text**. The data for the primary and secondary endpoints were collected at each time point. All the efficacy analyses were conducted in a modified intention-to-treat manner, including all randomized subjects who were not lost to follow-up. For the baseline variables, the summary statistics were expressed as frequencies and proportions for categorical data and mean (SD) or median (interquartile range) for continuous variables.

For the primary analysis, the mean percentage change in the mean CCA-IMT from baseline to 24 months and its 95% confidence interval (CI), estimated by analysis of covariance (ANCOVA) using the allocation adjustment factors as covariates, was compared between the treatment groups. Analysis of the primary endpoint was also done in subgroups stratified by the allocation and prespecified factors. Although we did not impute missing data for the primary analysis, a sensitivity analysis using a mixed-effects model for repeated measures (MMRM) was performed to examine the effect of these missing data. Comparisons of changes in the other efficacy endpoints between the treatment groups were performed with a Student unpaired *t* test.

All *P* values were two-sided with a level of significance of 0.05, and there were no adjustments for multiple comparisons. All statistical analyses were performed using SAS software version 9.4.

## Results

### Description of patients and interventions

**Fig 1:**
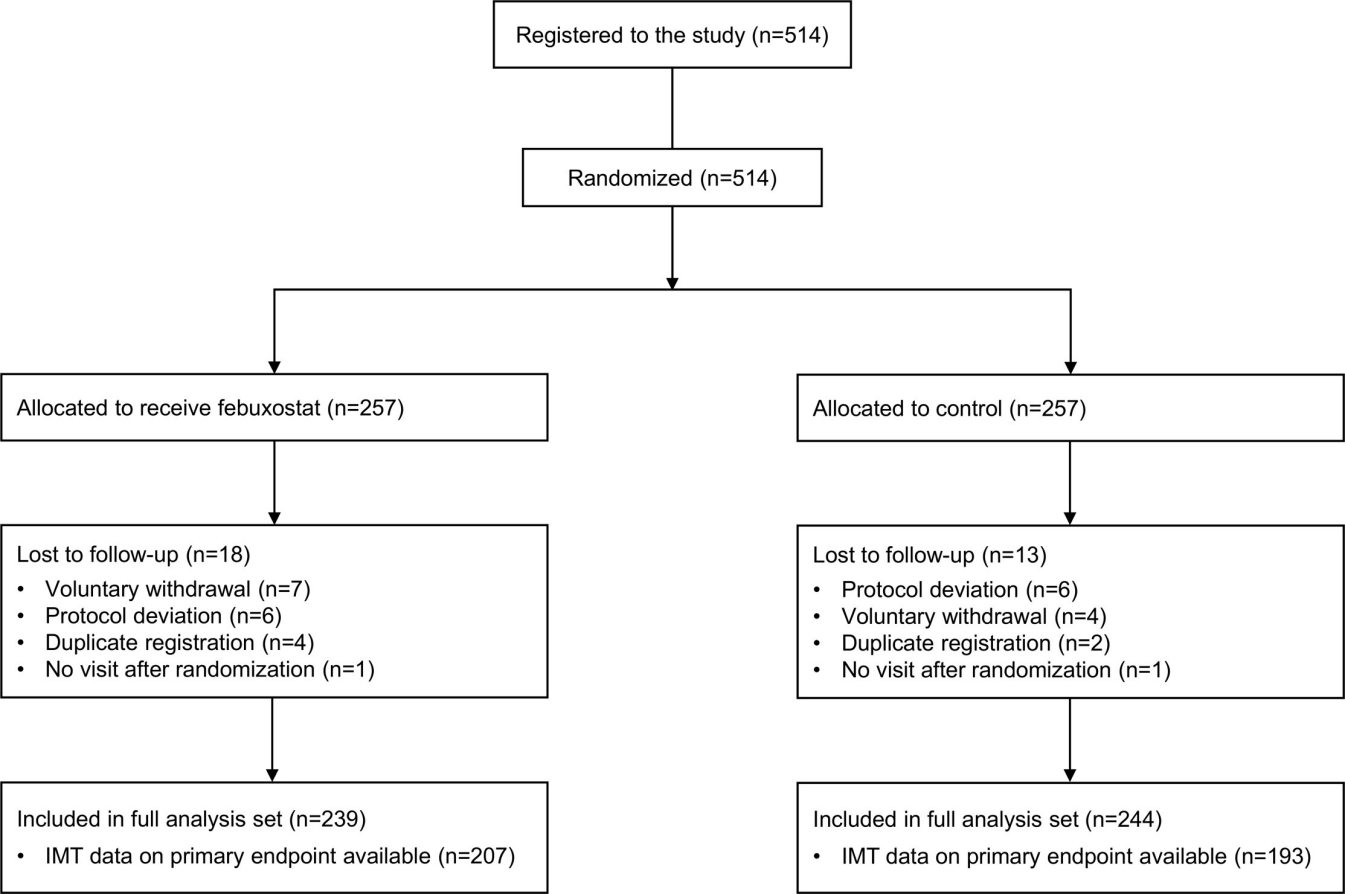
Flow of patients in the PRIZE study. IMT, intima-media thickness.

Between May 2014 and Jun 2016, 514 patients were registered in the study and assigned randomly to either the febuxostat group (*n* = 257) or the control group (*n* = 257) (**Fig 1**). A total of 31 patients (febuxostat, 18 patients; control, 13 patients) were lost to follow-up because of either duplicate registration, voluntary withdrawal, no visit after randomization, or protocol deviation. The study was completed by 86.0% of febuxostat-treated and 82.9% of control patients. Within the modified intention-to-treat population (full analysis set: febuxostat, 239 patients; control, 244 patients), 207 patients (86.6%) in the febuxostat group and 193 patients (79.1%) in the control group were included in the analysis of the primary endpoint. **Table 1** shows the baseline demographics and clinical characteristics of the full analysis population, and it appeared that the groups were well matched.

**Table 1:** Baseline demographics and clinical characteristics.

Variable	Febuxostat (*n* = 239)	Control (*n* = 244)
Sex, number (%)		
Women	49 (20.5)	46 (18.9)
Men	190 (79.5)	198 (81.1)
Age, year		
Mean (SD)	69.1 (10.1)	69.1 (10.7)
Median (IQR)	70 (63–76)	71 (63–78)
BMI, mean (SD), kg/m^2^	24.8 (3.9)	25.0 (3.5)
Current smoker, number (%)	28 (11.7)	24 (9.8)
Blood pressure, mean (SD), mm Hg		
Systolic	128.9 (14.7)	127.0 (15.7)
Diastolic	73.3 (11.6)	74.2 (11.4)
Medical history, number (%)		
Hypertension	212 (88.7)	217 (88.9)
Diabetes	85 (35.6)	88 (36.1)
Dyslipidemia	144 (60.3)	145 (59.4)
Previous gouty arthritis	6 (2.5)	4 (1.6)
Myocardial infarction	34 (14.2)	26 (10.7)
PCI	32 (13.4)	37 (15.2)
CABG	12 (5.0)	13 (5.3)
Stroke	14 (5.9)	12 (4.9)
Heart failure^a^	41 (17.2)	39 (16.0)
Prior medication, number (%)		
Any antihypertensive agent	217 (90.8)	222 (91.0)
Renin-angiotensin system inhibitor^b^	156 (65.3)	167 (68.4)
Calcium channel blocker	132 (55.2)	122 (50.0)
β-Blocker	86 (36.0)	96 (39.3)
Diuretic	70 (29.3)	70 (28.7)
Any lipid-reducing agent	126 (52.7)	129 (52.9)
Statins	115 (48.1)	124 (50.8)
Ezetimibe	10 (4.2)	6 (2.5)
Any antiplatelet agent	96 (40.2)	108 (44.3)
Aspirin	77 (32.2)	88 (36.1)

^a^Investigator reported.

^b^Angiotensin converting enzyme inhibitor or angiotensin receptor blocker.

Abbreviations: BMI, body mass index; CABG, coronary artery bypass grafting; IQR, interquartile range; PCI, percutaneous coronary intervention

In the febuxostat group, 33.9% of the patients received 10 mg, 23.5% received 20 mg, 2.3% received 30 mg, 29.4% received 40 mg, and 5.0% received 60 mg daily as the final adjusted dose of febuxostat (**S1 Fig**). The mean febuxostat daily dose was 22.8 (SD 15.9) mg at 24 months or termination for the full analysis set and 24.5 (SD 15.2) mg at 24 months or termination in those patients who remained on febuxostat until the end of the study.

### Carotid IMT

**Fig 2:**
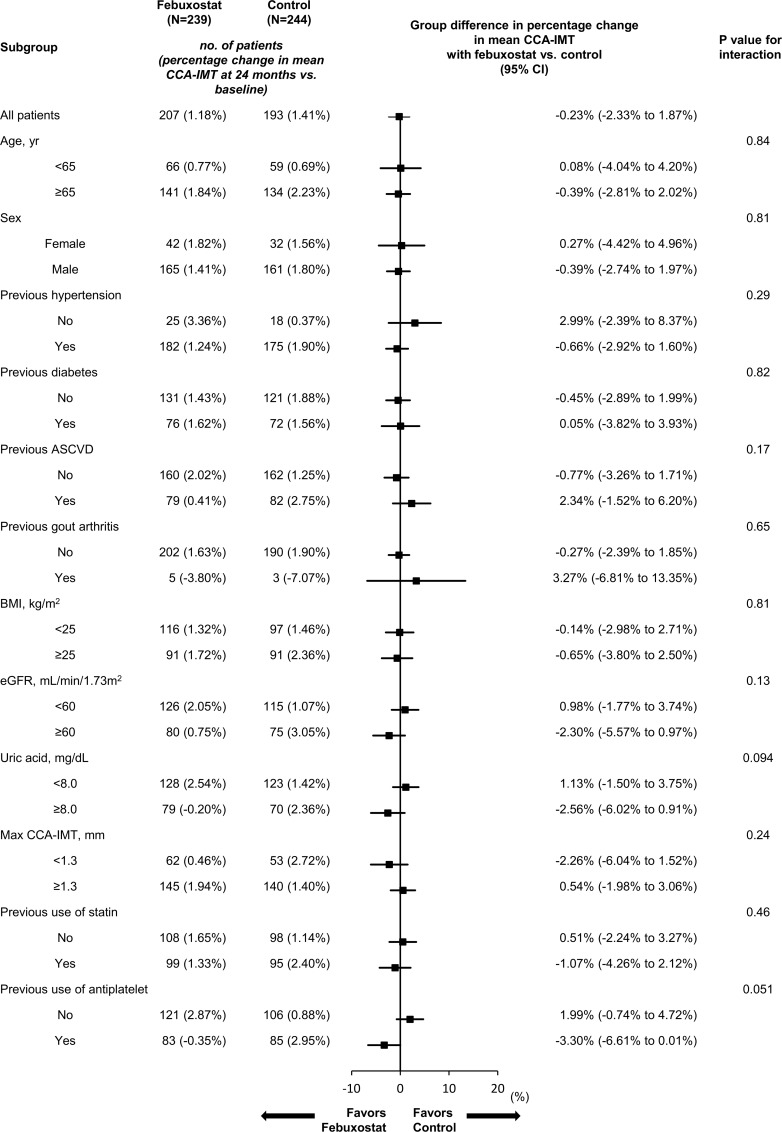
Subgroup analyses of percentage change from baseline to 24 months in the mean CCA-IMT. This shows the point estimate of the mean difference between the treatment groups (febuxostat − control) for the primary endpoint, stratified according to subgroups of the allocation and prespecified factors. The error bars represent the 95% CI. ASCVD, atherosclerotic cardiovascular disease; BMI, body mass index; CCA, common carotid artery; CI, confidence interval; eGFR, estimated glomerular filtration rate; IMT, intima-media thickness.

The baseline mean CCA-IMT values were 0.825 (SD 0.173) mm and 0.832 (SD 0.175) mm in the febuxostat and control groups, respectively. The absolute values and changes from baseline were comparable between the treatment groups at both 12 and 24 months (**Table 2**). For the primary endpoint, the allocation factors-adjusted mean percentage changes were 1.2% (95% CI −0.6% to 3.0%) in the febuxostat group and 1.4% (95% CI −0.5% to 3.3%) in the control group, with a mean between-group difference (febuxostat − control) −0.2% (95% CI −2.3% to 1.9%; *P* = 0.83). No significant difference was observed in the sensitivity analysis using the MMRM (**S2 Table**). Subgroup analyses showed febuxostat had an inconsistent effect without significant heterogeneity on the primary endpoint in the subgroups (**Fig 2**). However, in the febuxostat-treated patients, the subgroups with a SUA ≥8.0 mg/dL at baseline or those who received antiplatelet agents showed a tendency to have a reduction in the primary endpoint. Additional subgroup analyses in patients with an estimated glomerular filtration rate (eGFR) ≥60 mL/min/1.73 m^2^ at baseline also showed that the subgroup taking febuxostat with a higher SUA level (>8.0 mg/dL) tended to have a greater percentage change in mean CCA-IMT, although the difference between the two groups was not statistically significant (**S2 Fig**). Other carotid ultrasonographic values and their changes from baseline also did not differ between the treatment groups at any time point (**Table 2**).

**Table 2:** Changes in carotid ultrasonography parameters.

Endpoint	Time point	Febuxostat		Control		Group difference (95% CI)	*P* Value
		Number	Mean (SD)	Number	Mean (SD)		
Mean CCA-IMT, mm	Baseline	239	0.825 (0.173)	241	0.832 (0.175)	−0.007 (−0.039 to 0.024)	0.65
	12 months	207	0.826 (0.185)	210	0.841 (0.188)	−0.015 (−0.051 to 0.021)	0.42
	24 months	208	0.832 (0.182)	196	0.848 (0.176)	−0.016 (−0.051 to 0.019)	0.37
Delta from baseline, mm	12 months	207	0.003 (0.099)	208	0.012 (0.090)	−0.009 (−0.027 to 0.010)	0.35
	24 months	207	0.006 (0.087)	193	0.008 (0.090)	−0.002 (−0.020 to 0.015)	0.81
Percentage change from baseline	12 months	207	0.011 (0.115)	208	0.021 (0.110)	−0.010 (−0.032 to 0.011)	0.35
	24 months	207	0.015 (0.104)	193	0.018 (0.109)	−0.003 (−0.024 to 0.018)	0.81
Mean bulb-IMT, mm	Baseline	225	1.068 (0.342)	222	1.085 (0.364)	−0.017 (−0.083 to 0.048)	0.61
	12 months	194	1.062 (0.325)	196	1.089 (0.354)	−0.027 (−0.094 to 0.041)	0.44
	24 months	190	1.131 (0.380)	181	1.136 (0.384)	−0.005 (−0.083 to 0.073)	0.89
Delta from baseline, mm	12 months	185	−0.001 (0.263)	179	0.009 (0.258)	−0.010 (−0.064 to 0.044)	0.71
	24 months	178	0.065 (0.272)	169	0.030 (0.279)	0.036 (−0.023 to 0.094)	0.23
Percentage change from baseline	12 months	185	0.044 (0.230)	179	0.050 (0.244)	−0.006 (−0.055 to 0.043)	0.81
	24 months	178	0.104 (0.272)	169	0.064 (0.230)	0.041 (−0.013 to 0.094)	0.13
Mean ICA-IMT, mm	Baseline	145	0.789 (0.297)	155	0.773 (0.328)	0.016 (−0.055 to 0.087)	0.66
	12 months	154	0.840 (0.348)	139	0.822 (0.371)	0.017 (−0.066 to 0.101)	0.68
	24 months	134	0.846 (0.364)	126	0.848 (0.346)	−0.001 (−0.088 to 0.085)	0.98
Delta from baseline, mm	12 months	111	0.024 (0.233)	111	0.024 (0.345)	−0.0002 (−0.078 to 0.078)	>0.99
	24 months	102	0071 (0.268)	101	0.115 (0.276)	−0.044 (−0.119 to 0.031)	0.25
Percentage change from baseline	12 months	111	0.070 (0.273)	111	0.083 (0.315)	−0.013 (−0.091 to 0.065)	0.75
	24 months	102	0.136 (0.354)	101	0.179 (0.350)	−0.043 (−0.141 to 0.054)	0.38
Maximum CCA-IMT, mm	Baseline	239	1.040 (0.230)	241	1.056 (0.235)	−0.016 (−0.057 to 0.026)	0.46
	12 months	207	1.045 (0.254)	210	1.071 (0.264)	−0.025 (−0.075 to 0.024)	0.32
	24 months	208	1.044 (0.244)	197	1.070 (0.250)	−0.026 (−0.074 to 0.022)	0.29
Delta from baseline, mm	12 months	207	0.013 (0.159)	208	0.016 (0.151)	−0.003 (−0.033 to 0.027)	0.84
	24 months	207	0.003 (0.140)	194	0.008 (0.146)	−0.006 (−0.034 to 0.023)	0.70
Percentage change from baseline	12 months	207	0.020 (0.145)	208	0.025 (0.135)	−0.005 (−0.032 to 0.022)	0.74
	24 months	207	0.015 (0.132)	194	0.019 (0.133)	−0.004 (−0.030 to 0.022)	0.76
Maximum bulb-IMT, mm	Baseline	225	1.533 (0.518)	222	1.578 (0.604)	−0.045 (−0.150 to 0.060)	0.40
	12 months	194	1.591 (0.521)	196	1.633 (0.583)	−0.042 (−0.152 to 0.068)	0.45
	24 months	190	1.702 (0.628)	181	1.683 (0.621)	0.019 (−0.109 to 0.147)	0.77
Delta from baseline, mm	12 months	185	0.077 (0.458)	179	0.068 (0.476)	0.009 (−0.088 to 0.105)	0.86
	24 months	178	0.168 (0.470)	169	0.083 (0.485)	0.085 (−0.016 to 0.186)	0.098
Percentage change from baseline	12 months	185	0.119 (0.312)	179	0.107 (0.312)	0.012 (−0.052 to 0.076)	0.71
	24 months	178	0.171 (0.324)	169	0.112 (0.299)	0.059 (−0.007 to 0.125)	0.079
Maximum ICA-IMT, mm	Baseline	145	1.058 (0.380)	155	1.050 (0.457)	0.008 (−0.087 to 0.104)	0.86
	12 months	154	1.197 (0.521)	139	1.167 (0.559)	0.031 (−0.094 to 0.155)	0.63
	24 months	134	1.156 (0.526)	126	1.177 (0.551)	−0.021 (−0.153 to 0.111)	0.75
Delta from baseline, mm	12 months	111	0.080 (0.426)	111	0.074 (0.480)	0.006 (−0.114 to 0.126)	0.92
	24 months	102	0.098 (0.396)	101	0.187 (0.478)	−0.088 (−0.210 to 0.033)	0.15
Percentage change from baseline	12 months	111	0.128 (0.407)	111	0.134 (0.392)	−0.006 (−0.112 to 0.100)	0.91
	24 months	102	0.140 (0.371)	101	0.215 (0.447)	−0.075 (−0.189 to 0.039)	0.20
Plaque area, mm^2^	Baseline	203	7.176 (5.027)	194	6.874 (6.627)	0.302 (−0.863 to 1.467)	0.61
	12 months	85	7.971 (5.206)	91	8.248 (7.419)	−0.277 (−2.175 to 1.621)	0.77
	24 months	79	8.885 (5.665)	89	7.551 (7.109)	1.334 (−0.615 to 3.283)	0.18
Delta from baseline, mm	12 months	85	1.153 (4.695)	91	1.271 (6.235)	−0.117 (−1.753 to 1.519)	0.89
	24 months	79	1.133 (5.200)	89	0.954 (4.653)	0.179 (−1.332 to 1.691)	0.82
Percentage change from baseline	12 months	85	0.526 (1.281)	91	0.586 (1.702)	−0.060 (−0.506 to 0.387)	0.79
	24 months	79	0.526 (1.812)	89	0.338 (0.996)	0.188 (−0.267 to 0.642)	0.42
Plaque grayscale median	Baseline	203	46.732 (25.870)	194	46.339 (25.232)	0.393 (−4.650 to 5.436)	0.88
	12 months	85	54.648 (24.738)	91	54.503 (25.045)	0.145 (−7.264 to 7.554)	0.97
	24 months	79	54.145 (26.906)	89	52.451 (19.897)	1.694 (−5.599 to 8.987)	0.65
Delta from baseline, mm	12 months	85	3.332 (26.636)	91	9.810 (26.446)	−6.478 (−14.381 to 1.425)	0.11
	24 months	79	2.608 (30.699)	89	8.838 (21.664)	−6.231 (−14.432 to 1.970)	0.14
Percentage change from baseline	12 months	85	0.331 (1.326)	91	0.443 (0.759)	−0.112 (−0.437 to 0.214)	0.50
	24 months	79	0.226 (0.722)	89	0.486 (0.994)	−0.260 (−0.523 to 0.002)	0.052

Abbreviations: CCA, common carotid artery; CI, confidence interval; ICA, internal carotid artery; IMT, intima-media thickness; SD, standard deviation

### Effects on laboratory parameters (S3 Table)

**Fig 3:**
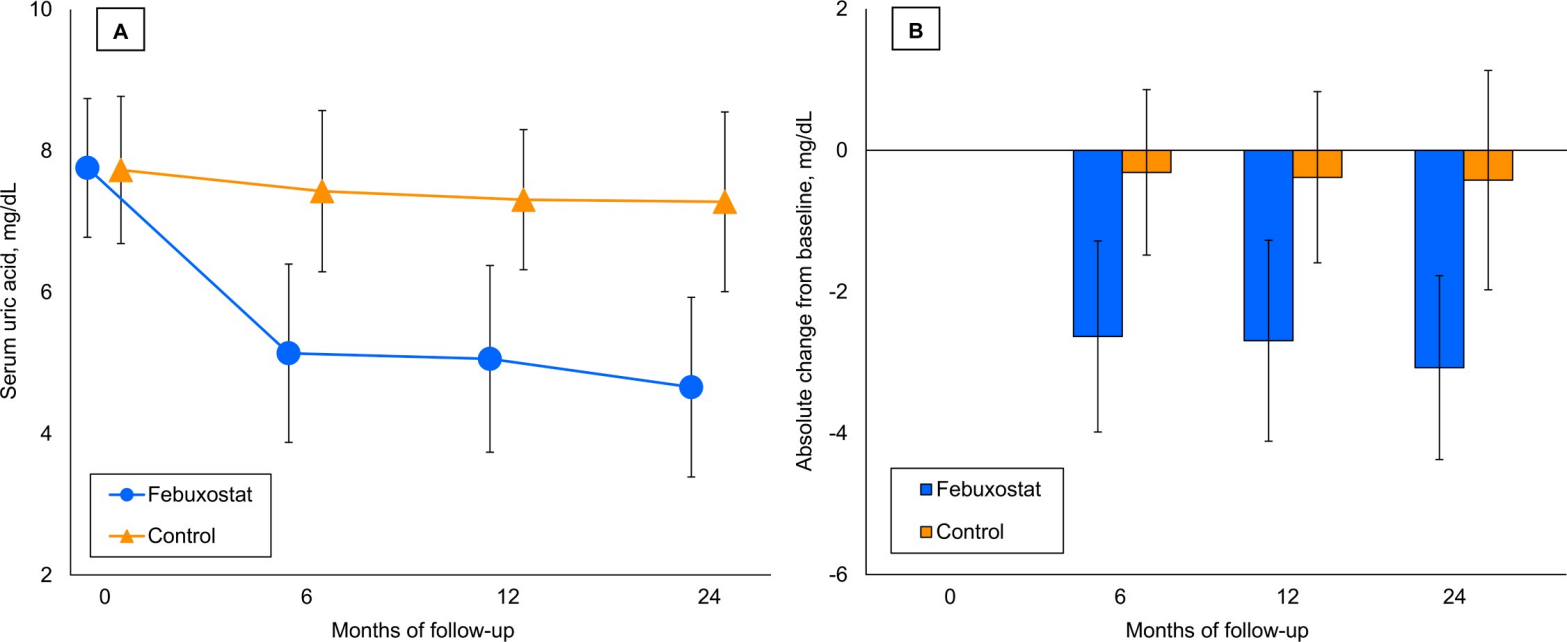
Changes in SUA. The values are expressed as means, with the error bars showing the SDs. (A) Changes in mean SUA. The mean values of SUA were lower in the febuxostat group at 6 months (febuxostat, 5.14 [SD 1.26] mg/dL versus control, 7.43 [SD 1.14] mg/dL; mean between-group difference [febuxostat − control] −2.28 mg/dL [95% CI −2.50 to −2.06 mg/dL ]; *P* < 0.001), 12 months (febuxostat: 5.06 [SD 1.32] mg/dL versus control, 7.31 [SD 0.99] mg/dL; mean between-group difference −2.25 mg/dL [95% CI −2.47 to −2.03 mg/dL]; *P* < 0.001), and 24 months (febuxostat, 4.66 [SD 1.27] mg/dL versus control, 7.28 [SD 1.27] mg/dL; mean between-group difference −2.62 mg/dL [95% CI −2.86 to −2.38 mg/dL]; *P* < 0.001). (B) Absolute changes in SUA. The mean between-group differences in the absolute change in SUA from baseline were also significant at 6 months (−2.31 mg/dL [95% CI −2.55 to −2.08 mg/dL]; *P* < 0.001), 12 months (−2.31 mg/dL [95% CI −2.56 to −2.06 mg/dL]; *P* < 0.001), and 24 months (−2.65 mg/dL [95% CI −2.93 to −2.37 mg/dL]; *P* < 0.001). CI, confidence interval; SD, standard deviation; SUA, serum uric acid.

At baseline, the SUA levels were 7.76 (SD 0.98) mg/dL and 7.73 (SD 1.04) mg/dL in the febuxostat and control groups, respectively. There were significant differences in the SUA levels between the treatment groups at 6, 12, and 24 months, and the final SUA levels were 4.66 (SD 1.27) mg/dL and 7.28 (SD 1.27) mg/dL in the febuxostat and control groups, respectively (**Fig 3**). The absolute reductions in SUA from baseline in the febuxostat group were also significantly greater than those in the control group at 6, 12, and 24 months.

Mean baseline systolic blood pressures were 128.9 (SD 14.7) mm Hg and 127.0 (SD 15.7) mm Hg in the febuxostat and control groups, respectively. Although a significant between-group difference in the absolute change from baseline in systolic blood pressure was observed at 12 months, there was no significant difference at 24 months. There were no significant between-group differences in the values and changes from baseline at 12 and 24 months in diastolic blood pressure and body mass index. In addition, there were no significant between-group differences in the values and changes from baseline at 12 and 24 months in the laboratory parameters.

### Adjudicated clinical events and adverse events

Information was collected from the safety analysis of those patients who had data collected following randomization (febuxostat, 241 patients; control, 247 patients). **Table 3** summarizes the number of patients with adjudicated clinical events. Overall, a numerically lower incidence of clinical events was observed in the febuxostat group compared with the control group. One cardiovascular death and three all-cause deaths occurred in the febuxostat group, whereas there were three cardiovascular deaths and seven all-cause deaths in the control group.

**Table 3:** Number of patients with adjudicated clinical events.

Adjudicated clinical event	Febuxostat (*n* = 241)	Control (*n* = 247)
Total, number (%)	9 (3.7)	16 (6.5)
Cardiovascular death	1 (0.4)	3 (1.2)
Nonfatal myocardial infarction	0 (0.0)	1 (0.4)
Nonfatal stroke	3 (1.2)	5 (2.0)
Composite renal events^a^	3 (1.2)	4 (1.6)
Non-cardiovascular death	2 (0.8)	4 (1.6)
All-cause death	3 (1.2)	7 (2.8)

^a^Composite of doubling of serum creatinine, initiation of hemodialysis, and renal transplantation.

A summary of the adverse events reported by the local investigators is shown in **S4 Table**. Overall, 52 patients (21.6%) had one or more adverse events (total 88 events) in the febuxostat group and 48 patients (19.4%) had them (83 events) in the control group. No gout arthritis occurred in the febuxostat group, whereas four patients (1.6%) developed gout arthritis in the control group. Impaired liver function and skin eruption were more common in the febuxostat group. Individual adverse events reported from the local investigators are listed in **S5 Table**. In the febuxostat group, 19 patients (7.9%) had adverse events that may have been caused by febuxostat administration. In these 19 patients, 11 discontinued febuxostat permanently, three discontinued it temporarily, and two decreased the dose.

## Discussion

### Main findings

The main finding of this multicenter, randomized study was that 24 months of febuxostat treatment did not delay carotid IMT progression compared with non-pharmacological lifestyle modification in Japanese patients with asymptomatic hyperuricemia, despite a significant difference in SUA levels between the groups. Febuxostat treatment was tolerable and did not increase the incidence of CVD and mortality in this population.

### XO inhibition and atherosclerosis

To our knowledge, this is the first randomized trial that has investigated whether or not lowering SUA concentrations using febuxostat delays the progression of carotid atherosclerosis in patients with asymptomatic hyperuricemia. To date, two randomized trials reported that short- and long-term treatment with a conventional XO inhibitor, allopurinol, could attenuate carotid IMT progression in patients with type 2 diabetes and asymptomatic hyperuricemia, and in patients with recent ischemic stroke or transient ischemic attack [21,22]. Due to mechanistic differences in their mode of action, febuxostat is known to have superior effects on XO inhibition and SUA reduction than allopurinol [9]. Furthermore, febuxostat is expected to exert superior anti-oxidative and anti-atherogenic activities [11], suggesting that febuxostat may have more benefit in the treatment of CVD than allopurinol. Although allopurinol was not used in the present study, febuxostat treatment for 24 months was at least not superior to non-pharmacological care for suppressing carotid atherosclerosis progression, despite the obvious urate-lowering effect of febuxostat.

### Febuxostat and CVD

Several previous studies investigated whether pharmacological urate-lowering therapy in patients with gout could improve cardiovascular outcomes, and the results were controversial [13,23,24]. A systematic review and meta-analysis using data based on conventional adverse-event reporting demonstrated that the clinical impact of febuxostat on the incidence of CVD was neutral compared with allopurinol or placebo, whereas there was an increased incidence of cardiovascular death with febuxostat treatment [25]. A large-scale cohort study also demonstrated no difference in the risk of CVD between febuxostat and allopurinol, but there was a trend towards an increased risk for all-cause mortality with long-term use of febuxostat [26]. In the CARES trial, conducted as a Food and Drug Administration (FDA) requirement for the evaluation of febuxostat safety, febuxostat did not increase or decrease the incidence of CVD relative to allopurinol, although there were significant increases in the incidence of cardiovascular and all-cause deaths in the febuxostat arm [12]. Thus, no study has shown a superior or inferior effect of febuxostat on the risk of atherosclerotic CVD, and this may partly support our findings.

A previous cohort study in a Japanese population without comorbidities demonstrated that asymptomatic hyperuricemia was associated with the development of cardiometabolic disease [3]. The Japanese guideline for the management of hyperuricemia and gout officially allows the use of pharmacological urate-lowering drugs in asymptomatic hyperuricemia [27], and this is different from the relevant guidelines in the United States and Europe [28,29]. In particular, the use of urate-lowering agents in asymptomatic hyperuricemia is allowed in patients with non-gout comorbidities, such as hypertension, diabetes, renal impairment, and CVD. In our study, most patients had such comorbidities at baseline but did not have active gout. In addition, their baseline SUA levels and the values of mean CCA-IMT at baseline were relatively low. Our findings suggest that febuxostat treatment for 24 months was not associated with an obvious anti-atherosclerotic effect, at least in the overall population studied. However, because subgroup analysis showed a tendency for febuxostat treatment in patients with SUA ≥8.0 mg/dL to delay CCA-IMT progression relative to non-pharmacological care for hyperuricemia, the effect of febuxostat on atherosclerosis may differ among patients with hyperuricemia according to the levels of SUA and be clinically beneficial in patients with higher SUA. In addition, we observed a trend of attenuated progression of carotid atherosclerosis in the subgroup of patients on febuxostat with less advanced carotid lesions, although this improvement was not statistically significant. This result raises the possibility that febuxostat was effective for attenuating earlier progression of carotid atherosclerosis, suggesting a limited role of febuxostat on the development of atherosclerosis. Further research is therefore warranted to determine the optimal patients with asymptomatic hyperuricemia who could potentially obtain cardiovascular and renal benefits from treatment with febuxostat [30].

Considering recent randomized trials with febuxostat in Japan, the FREED study showed that febuxostat reduced the risk of composite clinical events relative to non-febuxostat therapy in asymptomatic hyperuricemia, and that was largely driven by delaying the progression of renal dysfunction [31]. In the FEATHER study, febuxostat did not slow the decline of renal function compared with placebo in asymptomatic hyperuricemia and stage 3 chronic kidney disease [32]. In both studies, the incidence of cerebral and cardiovascular events was comparable between the treatment groups in asymptomatic hyperuricemia. Given the results of those studies and our findings, it may not be reasonable to use febuxostat to mitigate atherogenesis and subsequent CVD, at least in the overall population with asymptomatic hyperuricemia. However, febuxostat-mediated suppression of gout arthritis and renal impairment may improve quality of life and future prognosis in asymptomatic hyperuricemia [31,32]. Importantly, febuxostat treatment in Japanese patients with asymptomatic hyperuricemia did not show increased mortality in these randomized trials.

### Study limitations

This study has several limitations. First, this was an open-label study rather than a double-blind placebo-controlled trial, and the outcome might have been biased from the investigators’ choice of care. To minimize this possibility, all endpoints for carotid IMT were evaluated automatically in a blinded manner at a central core laboratory. Second, the actual dropout rate was higher than that originally estimated, and therefore the sample size might have been too small to detect a robust treatment effect on carotid IMT. Furthermore, the treatment period (24 months) might have been too short. Third, as in the recent randomized trials in Japanese [31,32], patients with symptomatic hyperuricemia were excluded from enrollment. This is the most striking difference between our study and other studies performed in regions other than Japan. In addition, the baseline SUA levels in our study were relatively low (7.7 mg/dL). Therefore, it may not be possible to generalize our findings to symptomatic hyperuricemia or to other ethnic groups. We also did not compare the effect of febuxostat with conventional allopurinol. Fourth, the values of mean CCA-IMT were relatively low despite the inclusion of patients with a maximum CCA-IMT ≥1.1 mm, which indicates carotid arterial plaque [15]. Such a finding has recently been shown to be a significant risk factor for future CVD in the general Japanese population [33]. This suggests that it would have been difficult to decrease these values further using the study intervention, possibly affecting, in part, the IMTs measured in the study. Moreover, there is evidence that the cardiovascular risk factors, hypertension and hypercholesterolemia, have different effects on the two components (i.e., the intima and media) of the IMT in pig carotid arteries [34]. It is therefore possible that hyperuricemia and XO inhibitors may have similar effects. However, because current carotid ultrasonography techniques used in the clinical setting are not able to measure these two components separately, it was not possible to determine which component was more affected in our study. Fifth, the change in scores used as primary and secondary endpoints in this study could have occurred as a result of either deleting missing data, ignoring the third time point, or as a consequence of measurement errors. Because the same patients were measured multiple times during the study, a better method than ANCOVA might be needed in order to take into account the effect of those errors. Lastly, different dosages of febuxostat were used in the febuxostat arm. The final doses of febuxostat were lower than expected, with one third of the study participants remaining on 10 mg of febuxostat after 24 months. It was therefore not possible to assess any vascular benefits of febuxostat per se, independent of the urate-lowering effect. Further research is therefore needed to determine the effects of febuxostat on atherosclerosis and cardiovascular safety in patients with hyperuricemia irrespective of gout.

In conclusion, in Japanese patients with asymptomatic hyperuricemia, 24 months of febuxostat treatment had no obvious effect on carotid atherosclerosis progression, compared with non-pharmacological lifestyle modification. These findings do not support the use of febuxostat for delaying carotid atherosclerosis in this population.

## Supporting information

S1 CONSORT Checklist (DOC)

S1 Text Study protocol. (DOCX)

S2 Text Event evaluation statement. (DOCX)

S3 Text Carotid scan measurement manual. (DOCX)

S4 Text Statistical analysis plan. (DOCX)

S5 Text The PRIZE study organization and investigators. (DOCX)

S1 Table Inclusion and exclusion criteria for the PRIZE study. (DOCX)

S2 Table Sensitive analysis for primary endpoint. (DOCX)

S3 Table Changes in clinical and laboratory parameters. (DOCX)

S4 Table Summary of adverse events. (DOCX)

S5 Table Individual adverse events reported. (DOCX)

S1 Fig Dose of febuxostat. (TIF)

S2 Fig Additional subgroup analysis of the percentage change from baseline to 24 months in mean CCA-IMT in patients with a normal eGFR (≥60 mL/min/1.73 m^2^) and hyperuricemia (>7.0 mg/dL). CCA, common carotid artery; eGFR, estimated glomerular filtration rate; IMT, intima-media thickness
(TIF)

## Abbreviations

ANCOVA: analysis of covariance
CCA: common carotid artery
CI: confidence interval
CVD: cardiovascular disease
eGFR: estimated glomerular filtration rate
FDA: Food and Drug Administration
ICA: internal carotid artery
IMT: intima-media thickness
MMRM: mixed-effects model for repeated measures
SD: standard deviation
SUA: serum uric acid; XO, xanthine oxidase
