# Supplementary material for: Febuxostat does not delay progression of carotid atherosclerosis in patients with asymptomatic hyperuricemia: A randomized, controlled trial
Source: PLoS Med. 2020 Apr 22;17(4):e1003095. doi: 10.1371/journal.pmed.1003095 (PMC7176100; doi:10.1371/journal.pmed.1003095)
Supplement: S1 Text — (DOCX) [file pmed.1003095.s002.docx]

**S1 Text.**

**Study protocol**

Program of vascular evaluation under uric acid control
by xanthine oxidase inhibitor, febuxostat: multicenter, randomized controlled study

PRIZE study

Principal Investigator: Prof. Koichi Node

Department of Cardiovascular Medicine

Saga University

5-1-1 Nabeshima, Saga 849-8501

TEL: 81-952-34-2364 FAX: 81-952-34-2089

Date of Institutional Review Board approval to December 2019

Ver. 1.0 Written: January 8, 2014

**0　Summary**

**0.1　Study design**

Multicenter collaborative, prospective, randomized, open-label, blinded endpoint (PROBE)
 clinical studies

Randomization Adjustment Factors

・Age

・Sex

・Diabetic complications

・Serum uric acid level

・IMT

Enrollment

Randomization

Patients with hyperuricemia　N=500

Observation up to 24 months, tests, assessment

Primary endpoint (Intimal media thickness, IMT) under blinded central testing

Febuxostat group
N=250

Control group

N=250

経過観察

Lifestyle training for hyperuricemia

**0.2　Objectives**

The objective of this study is to evaluate the efficacy of febuxostat when administered to patients with hyperuricemia in decreasing uric acid levels, and to evaluate this effect in inhibiting arteriosclerosis as determined by measuring intima-media thickness (IMT) on carotid artery ultrasounds as an indicator of its efficacy in preventing the progression of arteriosclerosis.

**0.3　Subjects**

Patients with asymptomatic hyperuricemia who were anti-hyperuricemic treatment naïve

(see 3.1 and 3.2 for details)

**0.4　Investigational treatment**

Investigational treatment is defined as treatment after randomized allocation to 1 of the 2 groups described below.

(1) Febuxostat group: Febuxostat will be orally administered for 24 months from baseline.

(2) Control group: The natural course of the disease will be monitored for 24 months from baseline.

**0.5　Assessment criteria**

0.5.1 Primary endpoints

Percentage change over baseline after 24 months: common carotid artery mean IMT measured by carotid ultrasonography.

0.5.2 Secondary endpoints

(1) Carotid ultrasonography results (common carotid artery, bulbar, internal carotid artery) at baseline and after 6, 12, and 24 months: amount of change and percentage change compared to baseline. (Mean IMT percentage change of the common carotid artery from baseline to 24 months of treatment is the primary endpoint and will be excluded)

Mean IMT, Maximum IMT, Plaque area, Plaque echogenicity

(2) Serum uric acid levels at baseline and after 6, 12, and 24 months: amount of change and rate of change compared to baseline.

(3) Cardiovascular function test values at baseline, and after 12, or 24 months of treatment: amount of change and rate of change compared to baseline (optional test items).

Flow Mediated Dilation (FMD), pulse wave velocity (PWV), cardio-ankle vascular index (CAVI), echocardiography (systolic, diastolic function, left atrial diameter, left ventricular mass index), arteriosclerosis index (AI).

(4) The following clinical laboratory test values at baseline, and after 6, 12, and 24 months of treatment: amount of change and rate of change compared to baseline.

Serum lipids (TC, HDL-C, TG, LDL-C [Indirect method], non-HDL-C), serum creatinine, eGFR (converted), urinary albumin excretion volume (Corrected creatinine) [if complicated by diabetes mellitus], urinary L-FABP [if complicated by diabetes mellitus or CKD].

(5) Clinical laboratory test and cardiovascular function test values at baseline, and after 12, or 24 months of treatment: amount of change and rate of change compared to baseline

NT-proBNP, blood pressure (outpatient clinic)

(6) The following events at baseline, after 24 months of treatment, or if they occur before study discontinuation.

Composite events (cardiovascular death, non-fatal myocardial infarction and stroke, renal events, all-cause death) and the incidence of each event.

(7) Adverse events that occur between baseline to after 24 months of treatment or before study discontinuation.

0.5.3 Exploratory endpoints

(1) Clinical laboratory test and cardiovascular function test values at baseline, and after 12, or 24 months of treatment: amount of change and rate of change compared to baseline [items marked with an * are optional].

High sensitivity CRP, 1, 5AG, small particle LDL, RLP-C, MDA-LDL, serum cystatin C, RAGE, polymer adiponectin, high sensitivity tropinin I, ANGPTL2, blood pressure (home)*

**0.6　Target number of patients and duration of study**

(1) Target number of patients: 500

(febuxostat group: 250, control group: 250)

(2) Treatment observation period: From date of approval by the Clinical Research Ethics
 Review Committee to March 2018 (Enrollment period: January 2016)

(3) Study period: Clinical research institutional review board approval date - December 2019

**1　Background**

Hyperuricemia has been suggested as a risk factor for atherosclerosis along with hypertension, hyperlipidemia, and diabetes mellitus, and studies into the relationship between vascular endothelial dysfunction and uric acid are ongoing. Studies conducted within Japan have suggested a relationship between uric acid and carotid artery plaques, Flow Mediated Dilation (FMD) or worsening intima-media thickness (IMT).^1-3^ It appears that uric acid and xanthine oxidase (XO) enhance reactive oxygen series (ROS) production, and the increase in oxidative stress is known to play an important role in vascular damage causing endothelial damage in hyperuricemia.^4^ First, the uric acid itself affects the vascular endothelial cells causing production of ROS. The inflammation caused by the activation of inflammatory cytokines is believed to damage endothelial cells.^4^ Furthermore, when XO incorporates substrate, ROS is produced, leading to inactivation of nitrous oxide (NO) and inflammation due to oxidative stress, resulting in further damage to the endothelial cells.^4^ In addition, since uric acid is produced from XO, it has been pointed out that elevated uric acid levels may be useful as an oxidative stress marker.^5^ Taken together, investigators are now looking at the possible efficacy of XO inhibitors in preventing vascular endothelial damage.

In fact, many studies have shown that allopurinol, a traditional XO inhibitor that decreases uric acid levels, is effective against atherosclerosis. Meta-analyses were conducted on randomized comparative studies that evaluated endothelial function via flow-mediated vasodilation (FMD) and forearm blood flow (FBF).^6^ Results showed a difference of 2.5 in FMD (95% confidence interval, 0.15-4.84), and 68.8 in FBF (95% confidence interval, 18.7-118.9), representing improved parameters in the allopurinol groups (either 300 mg or 600 mg doses) over control. Arteriosclerosis has been evaluated using IMT as a primary endpoint in many randomized comparative studies with drugs such as statins.^7,8^ Anti-hyperuricemic drugs have not yet been assessed using IMT in clinical studies, but IMT and FMD are known to have a high level of correlation,^9-11^ and it should be possible to use IMT to validate an inhibitory effect on atherosclerosis progression in drugs that lower uric acid levels.

Febuxostat is a new antihyperuricemic agent that was approved for indications of "gout and hyperuricemia" in January, 2011. Febuxostat inhibits XO via a different mechanism of action than allopurinol. In addition, since it can be eliminated via multiple routes, it has been shown effective and safe in patients with mild to moderate renal impairment.^12^ Febuxostat inhibits activated enzymes more potently than allopurinol in vitro, and clinical research has also shown that febuxostat is more effective in lowering serum uric acid levels than allopurinol.^15^ In addition, febuxostat expressed superior antihyperuricemic effects over allopurinol in a randomized, comparative study in patients with hyperuricemia after cardiac surgery.^16^ Febuxostat was also significantly more effective in improving renal function, reducing oxidative stress, and enhancing cardiac function than allopurinol.^16^ Based on the above, it appears we can expect febuxostat to express a more potent inhibitory effect on XO than allopurinol.

We therefore decided to evaluate if febuxostat in patients with hyperuricemia might be effective in preventing the progression of atherosclerosis using IMT as an indicator. In asymptomatic hyperuricemia where the patient has yet to develop gouty arthritis, there is no evidence that the use of an antihyperuricemic agent might inhibit cardiovacular damage in an interventional study, and it appears that guidance to correct lifestyle practices including diet is most important in terms of treating this condition. In this study, all patients were counseled regarding lifestyle habits that are generally observed in those with hyperuricemia, and this clinical study seeks to validate the efficacy of febuxostat administration.

**2　Objectives**

To evaluate the effect of prevention of atherosclerosis progression after anti-hyperuricemic treatment with febuxostat in patients with hyperuricemia using carotid intima-media thickness (IMT) as an indicator.

**2.1　Research hypothesis**

After investigational treatment with febuxostat administration (febuxostat group) or observation (control group) in patients with hyperuricemia for 24 months, inhibition of IMT progression will be significantly superior in the febuxostat group compared to the control group.

**3　Subjects**

This study will be conducted in patients with hyperuricemia who have not been receiving any anti-hyperuricemic treatment. Those who fulfill the following selection criteria and do not violate any of the exclusion criteria will be selected as subjects of this study.

**3.1　Inclusion criteria**

(1) Patients aged ≥20 years at the time informed consent is obtained (both sexes)

(2) Patients with asymptomatic hyperuricemia with serum uric acid level >7.0 mg/dL

(3) Patients with a maximum IMT ≥1.1 mm

(4) Patients who have given written informed consent to participate in the study

〔Rationale〕

(1) To ensure that our subjects reflect the kind of patients observed in actual clinical practice, no age or age limitations were established, only requiring that patients be legal adults at the time of informed consent.

(2) The lower limit of serum uric acid levels was established in accordance with the hyperuricemia, gout treatment guidelines.^17^ Hyperuricemia refers to a serum uric acid concentration that exceeds solubility in body fluids, >7.0 mg/dL.

(3) Atherosclerosis was established based on the presence of plaques and an IMT ≥1.1 mm.^18^

(4) Established for ethical reasons

**3.2　Exclusion criteria**

(1) Those who have been administered one of the following drugs to treat hyperuricemia prior to 8 weeks of the start of eligibility assessment.

allopurinol, benzbromarone, probenecid, bucolome, topiroxostat, febuxostat.

(2) Patients being treated with any of the following agents at the time eligibility criteria are confirmed:

Mercaptopurine hydrate, azathioprine, vidarabine, didanosine

(3) When assessing study eligibility, patients due to have surgery, or in the immediate post-surgical phase, or those with severe infections or serious trauma.

(4) Patients who have had a myocardial infarction or angina pectoris, percutaneous transluminal coronary angioplasty/bypass surgery, cerebral infarction, cerebral hemorrhage, subarachnoid hemorrhage, or a transient cerebral ischemic attack within 3 months of the confirmation of the eligibility criteria

(5) Patients with cardiac dysfunction (NYHA class IV)

(6) Patients with gouty tophus, or those who have had symptoms of gouty arthritis within 1 year of the confirmation of the eligibility criteria

(7) Patients with severe renal dysfunction (eGFR <30 mL/min or patients undergoing dialysis)

(8) Patients with severe liver dysfunction (AST or ALT ≥ 2-fold the upper limit of the institutional reference value)

(9) Patients with malignant tumors, or other complications or medical histories that the investigator feels may pose problems with study participation

(10) Patients who have a history of hypersensitivity to compounds in the febuxostat formulation

(11) Patients who are pregnant or nursing, who may be pregnant, or who may become pregnant during the course of the clinical study

(12) Other patients who are deemed unsuitable for inclusion in this clinical study based on the investigators' discretion

〔Rationale〕

(1) and (3) to (5) were established in consideration of the effect on efficacy evaluation of the test treatment. The administration of antihyperuricemic agents is a treatment method prohibited as a concomitant treatment in this study

(2) and (7) to (11) were established in consideration for the safety of patients. Both mercaptopurine hydrate and azathioprine in (2) are contraindicated for concurrent use with febuxostat. Vidarabine and didanosine should only be used with caution during concurrent febuxostat, and all of these drugs are prohibited concomitant treatments of this study

(6) There is a strong need for anti-hyperuricemic treatment in this population and we felt it would be inappropriate to include these patients into this study where they might be allocated to an observation only treatment group and so they were excluded from the study.

(12) This item is included to allow investigators to exclude subjects for reasons that had not been identified as inappropriate for the study during the planning stages.

**4　Study protocol**

**4.1　Study design**

A multicenter, prospective, randomized, open-label, blinded endpoint (PROBE) clinical study design was adopted for use in this study.

Study duration for individual subjects will be from registration to conclusion of the study (24 months from baseline) or until study discontinuation. During the study, patients in both groups will be counseled on lifestyle changes to improve their hyperuricemia, and also start test treatment (febuxostat administration or observation). This study will be conducted in accordance with the PROBE study design where the primary endpoint of intima-media thickness (IMT) could be measured in a central facility under blinded conditions.

(4wk)

(8wk)

(12wk)

0weeks

Informed consent

Lifestyle related guidance

40 mg

6mongt

10 mg

（60 mg）

20 mg

12mon

Study treatment continued for 24months

Febuoxstat group

Control group

24mon
study completion

Eligibility, enrollment, allocation

Within -2

months

(4 weeks), (8 weeks), (12 weeks): Dose will be increased at the discretion of the physician in charge, but should be increased to 40 mg within 6 months.

[Study design rationale]

This study is a randomized comparative study in patients with hyperuricemia to prove that the antihyperuricemic drug febuxostat is effective in preventing the progression of arteriosclerosis. Since it would have been difficult to administer a placebo under double-blinded conditions in this clinical study, we decided to conduct an open-label study and established an observation group as the control. In order to ensure objectivity, the PROBE study design was adopted and IMT, the primary endpoint, was measured under blinded conditions at a central testing facility.

Preceding research involved creating a manual on how to conduct IMT measurements and use the results.^19^ Various educational projects will be established such as training sessions for technicians who conduct the measurements; and by establishing a central testing facility, it will be possible to minimize measurement errors. In this study, as with preceding research, the IMT measurement manual will be used. Using the same organization and facilities as in the preceding research, and by conducting IMT measurement at a central facility under blinded conditions, it will be possible to minimize IMT measurement errors.

In cases of hyperuricemia with complications, drug treatment will be taken into consideration if the serum uric acid levels exceed 8.0 mg/dL, and in hyperuricemia without complications, if the uric acid levels are ≥9.0 mg/dL.^17^ Subjects in this study were patients with asymptomatic hyperuricemia (uric acid levels 7.0 mg/dL). All patients were counseled regarding lifestyle-related habits in an attempt to lower serum uric acid levels through behavior, and in the febuxostat group, patients will also be given febuxostat, while the control group will just be kept under observation.

If the serum uric acid level exceeds 12.0 mg/dL, the study should be discontinued since the patient would require treatment, and the appropriate measures should be instituted. Even if the investigational treatment is discontinued, follow-up observations should be continued as long as possible.

Febuxostat should be administered as specified in the package insert. Since the antihyperuricemic effects and ROS production inhibition via XO inhibition increase dose-dependently, the 40 mg maintenance dose specified in the package insert should be considered merely a general rule, and increased to the maximum dose of 60 mg per day if possible. Each patient should be treated for no more than 24 months based on the results of the preceding statin study (METEOR), where IMT progression was shown to be inhibited after a treatment period of 24 months.^20^

4.1.1 Target number of patients and study period

(1) Target number of patients: A total of 500 patients (Febuxostat group: 250, Control group: 250).

(2) Treatment observation period: From date of approval by the Clinical Research Ethics Review Committee to March 2018 (Enrollment period: January 2016)

(3) Study period: Date of Institutional Review Board approval to December 2019

(4) Rationale:

Refer to "8.3 Rationale for patient target number" in setting the number of patients for this study.

While the target number of patients is 500, since 50 medical institutions will be participating in the study, we estimated that it would take 2 years to recruit the necessary number of subjects. Since the study duration for individual patients is approximately 2 years, and taking into account the time required for special blood tests and data analysis, the study period was established as shown above.

**4.2　Selection of patients**

The investigators will select potential study subjects based on information obtained from patients during standard clinical practice. If the investigator finds the patient may be eligible for this study, details regarding the study will be explained to the patient and informed consent obtained.

**4.3　Explanation and informed consent**

The investigator will use the "information pamphlet" to explain the details of this study to the potential candidates and obtain voluntary written informed consent from these patients using the informed consent form (also refer to 10.2).

**4.4** **Confirmation of Subject Eligibility**

The investigator will confirm the subject eligibility (3.1, 3.2) of all patients from whom Informed Consent has been obtained.

A carotid duplex test was performed, and the subject is checked for eligibility based on the IMT value for that facility, and enrolled (allocation) if suitable.

**4.5　Enrollment and randomization**

4.5.1 Enrollment and allocation of patients will be conducted through the registration website. After confirmation of eligibility, the investigator will promptly access the registration website and enter the necessary information and send it. Information sent will immediately checked on the registration website and if appropriate, the patient will be registered into the database as a subject and an investigational treatment will be allotted. If the patient is not appropriate for inclusion, a notice to that effect will be sent out.

4.5.2 Establishment of the patient ID number

Patient registration numbers will be assigned based on the following format (XX-YYY).

XX: Study facility number

YYY: Patients at each study facility in order of enrollment

4.5.3 Randomization and randomization adjustment factors

Subjects will be randomized by a central registration system. The following randomization adjustment factors will be used to perform active allocation and patients will be allocated to the febuxostat and control groups at a 1:1 ratio. Details regarding the allocation procedures will be determined by the statistician and the investigators will not be notified of the procedure.

(1) Age (<65 years or ≥65 years of age)

(2) Gender (male/female)

(3) Positive for diabetes mellitus (yes/no)

(4) Serum uric acid (<8.0 mg/dL or ≥8.0 mg/dL)

(5) IMT (measured at each local site) (<1.3 or ≥1.3)

[Rationale for establishing adjustable allocation factors]

(1) Since age will have an impact on IMT, which is the primary endpoint, elderly and non-elderly patients were adjusted.

(2) Relationships between serum uric acid levels and cardiovascular event risk differs between men and women^21^ and this is taken into account when establishing groups.

(3) Since this would affect IMT, the primary endpoint, IMT was established as an adjustment factor.

(4) In patients positive for metabolic syndrome (+), the severity of hyperuricemia (≥7.9 mg/dL) tended to result in lower FMD (adjusted value) compared to patients without hyperuricemia or only mild hyperuricemia,^2^ and so this was established as an adjustment factor.

(5) Established based on the median IMT at baseline in preceding studies using statins.^8^

**4.6** **Baseline studies and start of investigational treatment**

The investigator will conduct a baseline survey within 2 months of obtaining informed consent and if the patient is eligible, investigational treatment will be initiated. The investigator will conduct baseline observations, tests, and evaluation of patients who have been enrolled into this study. In cases where patient ineligibility becomes apparent only after enrollment or if investigational treatment cannot be started for some reason, the study should be terminated in the relevant patient and the reason documented in the survey form.

In the febuxostat group, the investigator will prescribe the febuxostat preparation according to the administration method specified in this study and instruct the subject to begin taking the medication.

**4.7　Investigational treatment**

This is defined as “investigational treatment” in the following 2 groups based on random allocation at enrollment.

(1) Febuxostat group: Febuxostat will be orally administered for 24 months from baseline.

(2) Control group: Patients will receive non-pharmacological treatment and will be followed for 24 months from baseline.

4.7.1 Investigational drug (febuxostat formulation)

Feburic® (10 mg tablets, 20 mg tablets, 40 mg tablets)

Common name: febuxostat (Febuxostat)

Chemical name: 2[-3-cyano-4(-2-methylpropoxy)phenyl]-4-methylthiazole-5-carboxylic acid

The investigators should see Feburic® package insert for details and will always have a copy of the most recent package insert for reference.

4.7.2 How to administer the investigational treatment (febuxostat).

In principle, administration should be as follows. The drug should be given once daily. When deciding whether or not to increase the dose, the investigator must confirm patient drug compliance and conduct examinations and tests as needed.

(1) The investigational treatment (febuxostat formulation) will be started at a dose of 10 mg/day

(2) Dose will be increased to 20 mg/day by 4 weeks after the start of investigational drug administration.

(3) The dose will be increased to 40 mg/day by 8 weeks after the start of investigational drug administration. Thereafter, as a rule, the dose will be maintained at 40 mg/day until 24 months after the start of investigational treatment administration.

(4) If possible, the dose should be increased to 60 mg/day beyond 12 weeks or more after the start of treatment

*Dose will be increased at the discretion of the physician in charge of individual patients, but should be increased to 60 mg within 6 months after starting the study.

4.7.3 Guidelines for treatment (febuxostat) administration

(1) During tapering of the febuxostat, if the serum uric acid level falls below 2.0 mg/dL or under, the dose should not be increased.

(2) If the serum uric acid level is 2.0 mg/dL or less, the dose should be lowered by 1 tablet (20 mg).

**4.8　Concomitant treatments during the study period**

During the study period, the following rules should be followed and other treatments may be used concurrently. Treatment of complications and adverse events will be handled as deemed appropriate by the investigator in charge.

4.8.1 Lifestyle-related guidance in hyperuricemia

For the duration of the clinical study, the investigator will use a lifestyle guidance prescription for this study to provide advice on lifestyle practices to all patients.

Hyperuricemia is a typical lifestyle-related disease and treatment guidelines recommend that lifestyle related guidance including dietary therapy, restriction of alcohol intake, and physical exercise are recommended regardless of whether drug therapy is instituted or not.^17^ Since all study sites should follow the same recommendations, lifestyle-related counseling will be provided using a lifestyle-related prescription.

4.8.2 Prohibited concomitant therapies

The administration of the following drugs is prohibited for the duration of the study. If a prohibited concomitant drug is administered during the study, the investigator should discontinue the concomitant therapy as quickly as possible. If discontinuation is not possible and administration must be continued, the patient should discontinue participation in the study. Prohibited concomitant treatment and restricted concomitant treatments must be studied from the time of eligibility to the end of the study.

1. Anti-hyperuricemia drugs (both groups)

Allopurinol, benzbromarone, probenecid, bucolome, topiroxostat, febuxostat (all treatments other than "febuxostat" are prohibited).

1. Concomitant use with febuxostat prohibited, concomitant use requires caution (febuxostat group): mercaptopurine hydrate, azathioprine, vidarabine, didanosine

〔Rationale〕

(1) may affect the efficacy analysis, while (2) was established in consideration of safety during the study.

4.8.3 Restricted concomitant use therapy

Starting, changing dose, or discontinuing the use of the following drugs for the duration of the study period should be avoided.

Anti-diabetic drugs, anti-platelet drugs, anti-hypertensive drugs, anti-hyperlipidemia drugs

〔Rationale〕

Established since these could affect the efficacy evaluation. Taking into account the fact that patients may require these drugs as part of their treatment, the drugs will be continued without making any changes in the drugs or doses as much as possible.

4.8.4 Treatment for complications

During the study period, complications will be treated as part of standard care. Any treatment that was started before the start of the study will be continued without changing the content as much as possible for the duration of the study.

**4.9　 Study conclusion, discontinuation of the study treatment**

If the investigational treatment is administered for 24 months from baseline, the patient will conclude the study and observation, tests, and assessment scheduled for the end of the study will be performed.

If any of the following situations should arise after registration, the investigator should discontinue study treatment in this patient. Observations, tests, and assessment should be conducted as promptly as possible when discontinuing investigational treatment. Patients should be observed after study discontinuation.

1. If serum uric acid levels are above 12.0 mg/dL

⇒The clinical laboratory test result that led to the decision should be studied

1. If arthritic gout is discovered

⇒Arthritic gout must be studied as an adverse event

1. If the investigator determines investigational treatment in the patient is difficult due to the onset of adverse events or an event

⇒The onset of the adverse event or event should be studied

1. If the use of prohibited concomitant treatment becomes inevitable

⇒How the prohibited concomitant treatment was used and any adverse events should be studied

(5) A patient-specific reason (moved away, changed hospitals, etc.) made it impossible to continue study participation

(6) If the patient asks to discontinue study treatment or asks to discontinue participation in the study, or wishes to withdraw consent

⇒The "Informed consent withdrawal form" should be used to acquire written proof from the patient to leave the clinical study.

(7) If for reasons besides (1) to (6), the investigators deem it difficult for the patient to continue to participate in the clinical study

⇒The reason should be studied

**5　Observation, tests, and assessment**

**5.1　Observation, tests, and assessment schedule**

| Time point  Item | Eligibility assessment | Baseline^2^ | During study treatment | | |
| --- | --- | --- | --- | --- | --- |
| Visit | - | 1 | 2 | 3 | (After study conclusion) or At discontinuation |
| Course (with baseline as starting point) | Registration | 0 weeks | 6 months | 12 months | 24 months or discontinuation |
| Acceptable range | After informed consent is obtained to before enrollment1^1^ | Within 2 months of informed consent | ±2 months | ±2 months | Date of completion ±2months,  at discontinuation or as promptly as possible |
| Patient characteristics | ○ |  |  |  |  |
| Use of concomitant therapy^3^ | ○ | ○ | ○ | ○ | ○ |
| Febuxostat administration conditions (febuxostat group alone) |  | ○^2^ | ○ | ○ | ○ |
| Height (baseline alone), body weight |  | ○ |  | ○ | ○ |
| Blood pressure, pulse |  | ○ | △ | ○ | ○ |
| Home-based blood pressure |  | △ | △ | △ | △ |
| Carotid artery ultrasound, common carotid artery, bulbar, internal carotid artery (IMT is centrally measured) | ○^1^/●^4^ |  |  | ● | ● |
| Cardiovascular function test ^5^ |  | △ |  | △ | △ |
| Appearance of adverse events |  |  |  |  |  |
| Appearance of events^6^ |  |  |  |  |  |
| Clinical laboratory test^7^: hematology (general hematology, blood biochemistry) | ○^1^ | ○ | △ | ○ | ○ |
| Clinical laboratory tests (test items)^8^: serum uric acid, serum lipids, serum creatinine (eGFR), urinary albumin excretion, urinary L-FABP | ○^1^ | ○ | ○ | ○ | ○ |
| Special blood biochemistry tests measured at core laboratoty^9^ |  | ● |  | ● | ● |
| Blood collection volume (mL) to determine median values | - | 12 |  | 12 | 12 |

○: items measured and studied in-house, △: elective items to be measured or studied in-house, ●: items where medians are determined.

^1^ IMT (in-house measurement), serum uric acid eGFR, AST, ALT are required to determine eligibility.

^2^ In the febuxostat group, the baseline is taken on the day drug is dosed, and febuxostat administration is initiated. Baseline surveys will be conducted to confirm registration results on the same day as registration.

^3^Prohibited concomitant drugs and treatments, restricted concomitant use treatment will be surveyed.

^4^ IMT values measured at each local site will be used to determine eligibility and registration (allocation) using IMT, and the centrally determined value will be used as the baseline.

^5^ Echocardiography (systolic, diastolic function, left atrial diameter, left ventricular weight coefficient), flow mediated dilation (FMD), pulse wave velocity (PWV), cardio-ankle vascular index (CAVI), arteriosclerosis index (AI).

^6^ Cardiovascular deaths, non-lethal myocardial infarction and stroke, renal events, all-cause deaths

^7^ (as a rule, fasting blood samples) measured in-house, and safety of investigational treatment is confirmed. If abnormal changes are observed, it will be studied as adverse event

Hematology (red blood cell count, white blood cell count, hemoglobin, hematocrit, platelet count), blood biochemistry (AST, ALT, LDH, BUN, Na, K, Cl, blood glucose)

^8^ (blood generally drawn under fasting conditions) institutionally determined, and the measurement is recorded in the survey form. If abnormal changes are observed, it will be studied as an adverse event.

Serum uric acid level, serum lipids (TC, HDL-C, TG, non-HDL-C, LDL-C [indirect method]), serum creatinine (eGFR), diabetic complications: urinary albumin excretion [corrected creatinine], diabetes mellitus or CKD complications: urinary L-FABP.

^9^ Special blood biochemistry tests (NT-proBNP, high sensitivity CRPC1,5AGCsmall particle LDL-C RLP-C, CMDA-LDLm Cserum cystatin C, CRAGE, polymer adiponectin, high sensitivity troponin I, ANGPTL2)

**5.2　Details on survey items**

5.2.1 Patient characteristics

The following items will be checked during eligibility assessment

Patient registration no., sex, date of birth, alcohol/tobacco use (yes/no), menopause (women)

complications, underlying disease (diabetes mellitus, renal disease, liver disease, cerebrovascular disease, hypertension, hyperlipidemia, gouty arthritis, etc.), current pharmacotherapy (prohibited concomitant drugs, restricted concomitant treatment), IMT (institutional measurements), blood serum uric acid, eGFR, AST, ALT

5.2.2 Concomitant therapies

At confirmation of eligibility, baseline, and after 6, 12, and 24 months of treatment (or at study discontinuation), it will be determined if the following concomitant treatments are being used (content, duration of use).

(1) Prohibited concomitant therapies

1) Antihyperuricemic drugs (both groups)

Allopurinol benzbromarone, probenecid, bucolome, topiroxostat, febuxostat ("investigational use in any group except the febuxostat group" is prohibited)

2) Contraindicated or use with caution (in just the febuxostat group) during concomitant use with febuxostat

mercaptopurine hydrate, azathioprine, vidarabine, didanosine

(2) Restricted concomitant treatment

Anti-diabetic agents, anti-platelet agents, anti-hypertensive agents, lipid metabolism disorder drugs

5.2.3 Administration of febuxostat (febuxostat group only)

Administration conditions (dosage and administration, duration of treatment) will be determined at baseline and after 6, 12, or 24 months of administration (or at study discontinuation).

Conditions will be determined when adjusting febuxostat dose within 6 months after the start of treatment.

5.2.4 Height, body weight

Heights will be measured at baseline. Body weights will be measured at baseline and after 12 and 24 months of treatment (or at discontinuation of treatment). BMI will be calculated from the height and body weight.

5.2.5 Blood pressure, pulse

Blood pressure (systolic/diastolic blood pressures) and pulse will be measured at baseline and after 6*, 12, and 24 months of treatment (or at study discontinuation). (items marked with a * are optional).

Office blood pressure and pulse will generally be measured in a sitting position after at least 5 minutes at rest. Measurement times will be kept as constant as possible throughout the study period. Home-based blood pressure measurement (early morning blood pressure) is optional. (This will only be available for patients with hypertension who happen to have sphygmomanometers in their home).

5.2.6 Carotid ultrasonography (IMT measurement)

Carotid artery ultrasounds (common carotid artery, bulbar, and internal carotid artery) will be taken at eligibility determination (institutional measurements are used at enrollment and allocation, and central measurements are used as baseline values), and after 12, and 24 months of treatment (or study discontinuation). Carotid artery ultrasound results will be sent to the IMT central measurement facility and measured centrally.

IMT will be measured as specified in the separately specified "carotid artery ultrasound procedure manual".

5.2.7 Cardiovascular function tests

Echocardiography (systolic function, diastolic function, left atrial diameter, left ventricular weight coefficient), flow mediated dilation (FMD), pulse wave velocity (PWV), cardio-ankle vascular index (CAVI), echocardiography and arteriosclerosis index (AI) will be determined at a clinical site where these tests can be performed at baseline, and after 12 and 24 months of treatment (or at study discontinuation). Results of measurements will be evaluated by the substudy managers.

Echocardiography and FMD tests will be performed in accordance with the separately established "echocardiography procedure manual" and "FMD procedure manual".

5.2.8 Onset of events

The following will be determined for any events (cardiovascular death, nonlethal myocardial infarction and stroke, renal events, and all-cause deaths) that appear between baseline and after 24 months of treatment:

Date of onset, outcome, and date of outcome, classification of event, and investigator findings and comments; details regarding assessment of these events will be defined separately.

5.2.9 Adverse events

Any adverse events that are subject to assessment (6.2) that appear between baseline and after 24 months or treatment (or at study discontinuation) will be studied

Details are provided in "6 Assessment and reporting of adverse events"

5.2.10 Clinical laboratory tests: blood tests (general hematology, blood biochemistry)

The following items will be measured at baseline, and at 6*, 12, and 24 months (or study discontinuation) (* is optional) to determine study treatment safety. If any abnormal changes are seen, they will be studied as possible adverse events.

(1) General hematology (red blood cell count, white blood cell count, hemoglobin, hematocrit, platelet count)

(2) Blood biochemistry (AST，ALT，LDH，BUN，Na，K，Cl，Blood glucose)

5.2.11 Clinical laboratory tests (assessment items)

The following items will be measured at individual study facilities at baseline, and after 6, 12, and 24 months of treatment (or at discontinuation) and the institutional measurements must be documented in the survey forms. Confirm the safety of the study treatment and if any abnormal changes are observed, check for adverse events.

(1) Serum uric acid level (includes during febuxostat dose changes)

(2) Serum lipids (TC, HDL-C, TG, non-HDL-C, LDL-C [indirect method]), serum creatinine.

eGFR is calculated according to the following formula provided by the Japanese Society of Nephrology CKD treatment guide 2012.

eGFR (mL/min/1.73m2) = 194 × Cr-1.094 x age-0.287 (multiply by 0.739 if female)

(3) Urinary albumin excretion (corrected creatinine) [in diabetic complications]

(4) Urinary L-FABP [in diabetes or CKD complications]

5.2.12 Central measurement of clinical laboratory tests (assessment item)

The following blood tests will be measured centrally at a central measurement facility. Procedures for samples sent for central measurement and reporting are detailed separately.

1. Special blood biochemistry tests

NT-proBNP, high sensitivity CRP, 1,5AG, small particle LDL, RLP-C, MDA-LDL, serum cystatin C, RAGE, polymer adiponectin, high sensitivity Troponin I, and ANGPTL2.

These items will be measured at a central facility at baseline and after 12 and 24 months of treatment (or at discontinuation).

**6　Assessment and reporting of adverse events**

Adverse events are defined as all unwanted symptoms and signs (includes clinical laboratory abnormalities), and have nothing to do with causality. If adverse events should occur, the clinical investigator will promptly take the necessary measures (tests, treatment, discontinuation of treatment) and attempt to ensure the patient's well-being.

The clinical investigator will assess and report the adverse events according to the following procedures.

**6.1　Evaluation of adverse events**

Adverse events in which the causal relationship of febuxostat administration cannot be ruled out will be considered adverse drug reactions of febuxostat. Adverse drug reactions to febuxostat that are listed in the febuxostat package insert will be considered known adverse drug reactions, and those that are not listed will be considered unknown adverse drug reactions.

In this study, the following adverse events that appeared between baseline and completion or discontinuation of the study will be subject to evaluation as adverse events.

(1) Serious adverse events (Regardless of whether there is any relationship to the investigational treatment).

(2) Adverse events associated with febuxostat administration (in the febuxostat group)

1) Known adverse events of the febuxostat formulation

2) Unknown adverse events of the febuxostat formulation

(3) Adverse events associated with this clinical study

**6.2　Assessment items**

When the investigator or subinvestigator checks for adverse events, the following items will be examined.

(1) Adverse event name (diagnosis), date of onset

(2) Severity (mild, moderate, or severe)

(3) Seriousness (serious, not serious)

(4) Relationship to study participation (yes, no, unknown)

(5) (in the febuxostat group) causal relationship to febuxostat administration (can be ruled out, cannot be ruled out [if ruling out causality, provide rationale]) (in the febuxostat group) countermeasures related to febuxostat administration (continued, dose decrease, drug suspended, drug discontinued) treatment (yes, no [if yes, document content])

(6) Outcome (recovered, improved, not recovered, recovered but with sequelae, death, unknown [if patient was not recovered or status was unknown at time survey was completed, provide a reason]), date that outcome was confirmed.

6.2.2 Severity

Severity should be rated according to the following 3-level scale based on the worst severity observed for this adverse event

(1) Mild: Although signs and symptoms are present, there is no disruption of daily life activities and no treatment is required.

(2) Moderate: Daily life activities are disrupted due to discomfort, or clinical condition is affected as a result of this symptom, and treatment is required.

(3) Severe: Activities of daily life become impossible or a clinically significant impact is observed.

6.2.3 Serious adverse events

(1) Fatal events

(2) Life-threatening events

(3) Events that require hospitalization or extension of hospitalization

(4) Events that result in severe impairment or disabilities of function

(5) Events that cause congenital abnormalities

(6) Other clinically significant events or reactions

In case of (6), even if the event is not immediately life-threatening or leads to death or hospitalization, items fall under this category if there is a risk of exposing the patient to danger, or if there is a vital medical issue where treatment or countermeasures are required to prevent the results defined in items (1) to (5).

6.2.4 Outcome

Outcomes include 6 levels "recovered, improved, not recovered, recovered but with sequelae, death, or unknown"

If patients are found to be “recovered, improved, or deceased” on the day outcome is confirmed, this date will be noted as the day of outcome confirmation. If the outcome is "not recovered" or "unknown" and follow-up is to be concluded, the final day that the investigator checked the outcome will be recorded as the date of follow-up and the reason why follow-up had to be discontinued or remained unknown will be documented in the comments field.

6.2.5 Causal relationship to febuxostat administration (in febuxostat group)

Causal relationship to febuxostat administration is assessed according to the following 2-item scale. If the event is assessed as "Causality can be ruled out", then the reason for this assessment should be documented in the comments section.

(1) Causality can be ruled out: The temporal sequence makes a relationship to febuxostat administration impossible or a medically relevant cause other than febuxostat administration is implicated.

(2) Related to the investigational treatment: If the above does not apply.

**6.3　Reporting adverse events**

In adverse events related to the investigational treatment, if a serious adverse event is noted, it should be reported in accordance with reporting by doctors, pharmacists, and other parties related to medical institutions of information regarding pharmaceutical side effects, infectious diseases, etc. to the Minister of Health, Labour and Welfare (Pharmaceutical Affairs Law Article 77-4-2) and Spontaneous reports by the MAH (Pharmaceutical Affairs Law Article 77-4-1) should be handled as appropriate by each study facility.

6.3.1 Emergency reports

If any serious adverse events (6.2.3) should occur, they will be subject to an emergency report. If the relevant adverse event is observed, the investigator will immediately notify the head of the research facility. When the investigator is made aware of the relevant adverse event, s/he will immediately document the required information in the adverse event report and fax it to the Research Secretariat at (03-3814-3388).

6.3.2 Normal reports

"Adverse events that are reported as part of the "6.1 adverse event assessment". If an adverse event subject to standard reporting should occur, the clinical investigator will document the specified information regarding the adverse event in the adverse event report and send it to the research secretariat or enter the information into EDC. The investigator should attempt to document the outcome of the adverse event for the duration of the study period as much as possible.

6.3.3 Efficacy and the safety evaluation committee

The principal investigator should report any emergency reports from the study institutions or secretariat to the safety evaluation committee in writing, and simultaneously ask for a committee review of the principal investigator’s comments and actions related to how the event was handled.

The safety evaluation committee will review the report and provide written recommendations to the principal investigator on how the case was handled, whether the study may be continued, and any future measures that should be instituted.

6.3.4 Responsibilities of the research secretariat

The clinical investigators will ask the principal investigator or his stead (clinical investigator in charge) and if necessary, the safety evaluation committee, for their judgement on how to handle the adverse event depending on the urgency, seriousness, and impact. In addition, if necessary, recruitment of new patients will temporarily be discontinued (all study facilities will be contacted) and the study facilities will take the necessary emergency contact measures to deal with the situation.

**6.4　Adverse drug reactions of the investigational treatment (febuxostat)**

Check the "package insert" for Feburic® tablets. For the most recent package insert information, please check the home page of the Pharmaceuticals and Medical Devices Agency (PMDA) at <http://www.info.pmda.go.jp/>.

**7　Study endpoints**

**7.1　Primary endpoint**

Percentage change in the mean IMT for the common carotid artery from baseline to after 24 months of treatment. Percentage changes in left and right common carotid arteries are averaged and then assessed.

〔Rationale〕

IMT is an established marker in assessing arteriosclerosis.^18^ Noninvasive morphological examinations of arteriosclerosis include ultrasound (carotid ultrasonography) tests and CT tests, and IMT is determined from ultrasound data.^18^ In addition to IMT, FMD and PWV are markers of arteriosclerosis, but since IMT is used as an endpoint in evaluating various drugs such as statins in randomized comparative studies, and since the measurement of IMT is standardized with relatively little risk of measurement error, and since IMT use (≥1.1 mm) is recommended as a marker of the degree of atherosclerosis in treatment guidelines,^18^ IMT was selected as the primary endpoint in this study.

**7.2　Secondary endpoints**

(1) Carotid artery ultrasound measurements (common carotid artery, bulbar, and internal carotid artery) at baseline and amount of change and percentage change from baseline to 12 and 24 months of treatment.

(The mean IMT from baseline after 24 months of treatment of the common carotid artery is excluded since this is the primary endpoint)

Mean IMT, Maximum IMT, plaque area, plaque echogenicity

(2) Baseline value of serum uric acid and amount of change and percentage change from baseline in serum uric acid levels after 6, 12, and 24 months of treatment.

(3) The following measurements, amount of change, and percentage change in cardiovascular function tests from baseline at baseline and after 12 and 24 months of treatment. (optional items)

Flow Mediated Dilation (FMD), Pulse Wave Velocity (PWV), Cardio-ankle vascular index (CAVI), IMT, Echocardiography (systolic, diastolic function, left atrial diameter, left ventricular weight coefficient, atherosclerosis index (AI)

(4) Amount of change and percentage change from baseline for the following clinical laboratory test items at baseline, and after 6 months, 12 months, and 24 months of treatment.

Serum Lipids (TC, HDL-C, TG, LDL-C [indirect method], non-HDL-C), serum creatinine, eGFR(converted), urinary albumin excretion (corrected for creatinine) [In case of diabetes complication], urinary L-FABP [diabetes or CKD complication]

(5) Values, amount of change, and percentage change in the following clinical laboratory tests and cardiovascular function tests at baseline, after 12 months, and after 24 months of treatment. (* is optional)

NT-proBNP, blood pressure (outpatient)

(6) All following events that occur from baseline until after 24 months of treatment or study discontinuation.

Onset of complex events (cardiovascular death, non-fatal myocardial infarction and stroke, renal event, total deaths) and each event

(7) Adverse events that occur between baseline to after 24 months of treatment or before study discontinuation.

For safety information, evaluate all adverse events subject to assessment (6.2).

**7.3　Exploratory endpoints**

(1) The following measurements, amount of change, and percentage change from baseline in clinical laboratory tests and cardiovascular function tests at baseline and after 12 and 24 months of treatment. (* are optional measurement items)

High sensitivity CRP, 1,5AG, smaller LDL particle, RLP-C, MDA-LDL, serum cystatin C, RAGE, high molecular weight adiponectin, high sensitivity troponin I, ANGPTL2, blood pressure (home)*

**8　Statistical methods**

**8.1　Analysis set**

The research secretariat will investigate individual cases for deviations from the study protocol and the seriousness of the deviation, dropouts from the study and the timing, and wishes of the patient on whether or not their data can be used in the data analysis before the following 3 are subjected to data analysis

1. Full analysis set (FAS)

The maximum set of all subjects comprising all subjects enrolled and allocated to a test treatment is called the full analysis set (FAS). However, any patients in serious violation of the study protocol (informed consent has not been obtained, registration outside of the specified time period, etc.) will be excluded from this group.

(2) Per protocol set (PPS): patients who fulfill the study protocol requirements

All of the FAS patients after excluding all those who deviate largely from the study protocol regarding the test treatment or concomitant treatment and with the following serious violations.

Eligibility criteria violation, exclusion criteria violation, concomitant drug violation, and concomitant therapy violation.

(3) Safety analysis set

Patients who were registered in this study and started the treatment allocated, who received part or all of the specified treatment.

8.1.2 Patients and data handling

The steering committee will decide how to handle the cases and data before starting statistical analysis. Cases and data handling will be determined by the data handling criteria established before data lock.

**8.2　Statistical analysis plan**

All efficacy analyses will be performed as the main analysis in the FAS, and as reference, analyses will be conducted in the patients who fulfill the PPS and match the per protocol set or study protocol requirements. Safety analyses will be conducted in the safety analysis set. Details are provided separately in the statistical analysis plan.

8.2.1 Analysis of patient characteristics

Distribution of patient characteristics for the various analysis sets and summary statistics will be calculated for each allocation group. Nominal and ordinal variables will be shown by the frequency and percentage of the allocated groups. Summary statistics will be calculated for continuous variables in each allocation group. The tests used in these studies for nominal variables will be Chi-squared tests (or Fisher's exact test as necessary), and for ordinal data, Wilcoxon's ranked sum test, and for continuous variables, a t-test will be employed (level of significance = two-tailed 0.05).

8.2.2 Primary endpoint analysis: percentage change in mean IMT of common carotid artery from baseline to after 24 months of treatment

The primary endpoint, IMT analysis will be conducted under allocation group blinding by the statistician independently of the clinical investigator. The main objectives of this study are to see if the percentage change in IMT decreases significantly after 24 months of treatment with febuxostat compared to the control group. The null hypothesis that the percentage change in IMT in both groups was equal will be tested and using the allocation adjustment factors as covariates, analysis of covariance will also be performed. To determine sensitivity of the analysis, profiles in IMT changes over time will be determined and the linear mixed effect model used for the profile measurement data, confirming there are no major differences from the results of the analysis of covariance. The level of significance will be a two-sided 5% and the two-sided 95% confidence interval will also be calculated.

8.2.3 Secondary endpoint analysis

Secondary endpoints will also be analyzed in order to supplement the main analytical results in this study. Adjustments for multiplicity will not be made in secondary endpoints. Group comparisons will be conducted as necessary. Details on methodology are documented separately in the statistical analysis plan.

8.2.4 Analysis of exploratory endpoints

In order to discover factors related to analysis of the data in this study, exploratory endpoints will be analyzed as needed.

8.2.5 Safety analysis

Safety endpoints will be assessed through evaluation of all adverse events subject to evaluation (6.2). Events will be classified by their MedDRA names and each item will be summarized in tables to conduct interval estimations for 2-item distribution to allow for an accurate two-sided 95% confidence interval calculation in each group. Fisher's exact test will be used to compare groups as necessary.

**8.3　Rationale for the target number of patients**

In this study, the percent change in mean IMT of the common carotid artery from baseline after 24 months of treatment in the control group (no treatment) versus the febuxostat group will be established as follows. The Tokyo Medical College FMD-J research (observation period 18 months) showed a rate of change of 1.96% in mean IMT of the common carotid artery from baseline in the data from 58 patients.^22^ This value will be extrapolated to 24 months using proportional calculations and the rate of change in mean IMT after 24 months of treatment compared to baseline would be 2.62%. We assumed that the standard deviation for IMT after 24 months would be equal to that after 18 months. Based on the above, percent change in the mean common carotid artery IMT in the control group of our study should equal 2.6±6.0 (%, mean±SD). On the other hand, to determine the percentage change in the febuxostat group, we looked at a report on pitavastatin administration.^8^ The change volume in the mean IMT for the common carotid artery after 12 months of treatment with pitavastatin (moderate) was -0.0078 (mm) which when compared with the baseline man IMT value of 0.94 (mm) led to a percentage change of -0.8%. For this reason, in our study, a conservative assumption regarding percentage change in mean IMT after 24 months of treatment with febuxostat in this study would be +1.0%, with a standard deviation of the same 6.0% as the control group.

Based on the above, assuming the difference in the percentage change in the mean IMT for common carotid artery between the control group and the febuxostat group was 1.60 (%), with a standard deviation of 6 (%) and a two-tailed level of significance of 5% with a detection level of 80%, the necessary subject count came to 222 patients per group. Allowing for a drop-out rate of approximately 10% during the course of the study, we set the target number of patients as 250 patients in each group or a total of 500 patients overall.

**9　Data collection**

**9.1　Submission of case report forms**

The investigator will enter the Case Report Form data into the EDC.

**9.2　Data management**

The data center will be managed as defined in a separate data management protocol. Reminders will be sent out to those who do not submit data, data will be checked for precision and submitted data questioned, correcting data as necessary depending on the results of such questions. Database management and statistical analysis dataset preparation will be performed as necessary.

**10　Ethics**

This study will be conducted in strict observance of the Declaration of Helsinki and the Ethical Guidelines for Clinical Research from the MHLW.

**10.1** **Ethics review**

Investigators will submit all necessary documentation in accordance with the regulations of
 each facility and receive approval to conduct this study from the Ethics Review Committee of
 their facility. If the Ethics Review Committee approval is obtained, a copy of the approval will
 be sent to the Research Secretariat.

Annual updates to the Ethics Review Committee approval will be handled as specified at each
 study site.**10.2　Explanation and informed consent**

Before checking for eligibility, clinical investigators must provide the patients with an institutional review board-approved information pamphlet and explain the study orally. After explaining the study to the patient, the investigator must confirm that the subject understands the content of the research and ask for his or her participation in the study. Using the informed consent form, written informed consent must be obtained of the patient's own free will.

If the patient agrees to participate in the study, the patient will write the date and sign the informed consent form. In addition, the clinical investigator who explained the study and CRC (if the CRC provides additional information) will document the date that they gave their explanations and sign and stamp the consent form. A copy of the informed consent form and information pamphlet will be prepared and given to the patient, while the original informed consent form will be stored at the study facility.

10.2.1 If any information that might influence participation in the clinical study comes to light during the course of the study

(1) If the clinical investigator obtains any new information that could affect the patient's wish to continue to participate in the clinical study, the patient must immediately be made aware of this information in written form and the patient reminded that the patient is free to leave the study at any time.

(2) The informational pamphlet and informed consent form will be signed by the investigator and the CRC (if the CRC has added information) with the date of explanation. The patient will record the date that he received the information and sign the informed consent form. A copy of these documents will be given to the patient. The clinical investigator will confirm whether the patient wishes to continue to participate in the study and after documenting the results of this confirmatory procedure on the information pamphlet and informed consent form, together with the date of confirmation, sign these documents. A copy of the informed consent form will be given to the patient, while the original consent form will be archived at the clinical study facility.

(3) The clinical investigator will revise the information pamphlet and informed consent form and if necessary receive institutional review board approval. The revised information pamphlet and informed consent form will be used in giving explanations; consent of whether or not to continue participation in the clinical study will be obtained of the patient's free will in writing. At this time, the clinical investigator and CRC (if the CRC adds information) will document their explanations in the informed consent form and sign it and the patient will document the date of consent and sign it. A copy of the informed consent form will be given to the patient and the original consent form will be archived at the clinical study facility.

**10.3　Protection of patient confidentiality**

10.3.1 Protection of the patient's privacy and personal information

All persons involved in this study will take sufficient care to protect the personal information and privacy of the patients. The head of the clinical study facility will ensure that adequate measures and systems are in place to ensure confidentiality of personal information and all necessary safety measures.

When publicizing the results of this study, no data that could potentially lead to identifying the subjects will be used. Subject data from the study will not be used for any purpose other than the objectives stated in this study. Should any of the subject data obtained in this study be used for any purpose other than those stated in the objectives of this study, consent must be obtained from the subjects in advance.

10.3.2 Method of data receipt and patient ID

Any information that could potentially lead to identifying the patient such as the patient's name or hospital patient ID will not be entered in the case report forms. All registered data will only include the case report form patient ID and this will be used to refer to the patients. Any information that could link the case report form ID and the participating individuals will be controlled by the clinical investigator.

**10.4　Potential benefits and disadvantages for study participants**

The patients who participate in this study will suffer no special advantages or disadvantages compared to those undergoing standard care. If an adverse event should occur during study participation or if investigational product efficacy is insufficient and it is deemed necessary to change to alternative treatment, the clinical investigator will make the utmost effort to quickly conduct the necessary tests, introduce countermeasures, and discontinue treatment, if necessary, to ensure the patient's safety and access to optimal care.

All treatment during the study period will be based on standard clinical care and covered as insured medical care. In the febuxostat group, the patient will be required to co-pay for the febuxostat formulation as insured medical treatment. All costs for central measurement in this study will be paid for by the clinical study. The amount of blood collected for central measurement in this study will be approximately 12 mL more than during a standard blood collection. Patients in this study will receive a small fee for their participation.

This is a randomized comparative study comparing febuxostat administration against observational care, and there is insufficient evidence regarding which of the two is superior. The premise is that the efficacy and safety of both group is equipoised. However, subjects in the febuxostat group may experience adverse drug reactions (see package insert). Since concomitant use of hyperuricemia treatment drugs is prohibited during the study period, they cannot be used, but in accordance with the treatment guidelines, all patients should receive lifestyle-related guidance.

**11　Quality control and quality assurance**

**11.1　Audit**

The objective is to confirm that this study is being conducted appropriately and to assure the reliability of the resulting data. The Audit responsibilities of this study will be outsourced to a CRO. In carrying out an audit, any records or medical data that may identify the subject must be kept confidential to maintain patient privacy.

**11.2　Archiving of records**

Records related to patient informed consent, patient ID number tables, and other related documents, raw data to create clinical study reports (test data, etc.), institutional review board approval, various records and documents prepared at individual study facilities, will all be archived by the study investigators. Data will be archived for a period of 3 years after study discontinuation or completion.

**11.3　Direct access to source documents, etc.**

If requested, the investigators and clinical study sites will permit direct inspection of all records related to this study for monitoring or auditing purposes or by the Ethics Review Committee, and cooperate in full.

**12　Costs to study participants and dealing with injury to health**

**12.1　Costs to the study participants**

All medical treatment conducted during the study period including investigational treatments will be handled in the same manner as standard clinical practice and will be paid for by the patient's medical insurance and patient co-payments. This research requires central measurement of blood samples and these costs will be funded by the study. To compensate the patients for their trouble in participating in the study, subjects will receive a 2000-yen prepaid QUO card at registration, 1 year after the start of the study, and after conclusion of the study (or at discontinuation).

**12.2　Dealing with health impairment**

If any impairment or damages arise from this study, all required treatment will be handled by the medical insurance of the patient as with standard clinical care. To ensure compensation will be available in case a patient suffers a health impairment resulting from this study, the investigators will take out clinical research insurance policies for all those involved. If any health impairment (physical impairment) should occur in the subjects due to participation in this clinical study, the investigators who are covered by this insurance will be protected from responsibility for compensation or any legal liabilities and will be able to pay damages through the availability of insurance money to cover such costs.

**13　Conflict of interest and source of research funding**

**13.1　Conflict of interest**

Decisions regarding the conception, planning, execution, analysis, and publication of this study will be made by the principal investigator and the steering committee. Individual investigators will manage matters to follow conflict of interest management policies of various academic societies and individual study facilities, and when requested for information regarding conflict of interest by the academic society or medical journal will disclose information appropriately.

**13.2　Source of research funds and roles of the sponsors**

The main funding for this study was provided by TEIJIN PHARMA Limited to Saga University based on a collaborative research contract.

The sponsor, TEIJIN PHARMA LIMITED will be involved in providing information for the conception and test product provision (febuxostat formulation) process to ensure proper use, but will not be involved in the planning, execution, analysis, and publication process.

**13.3　Collaborating institutions measuring biomarkers**

NT-proBNP will be measured through collaborative research between Roche Diagnostics K.K. and Saga University.

High-sensitivity Troponin I will be measured under a collaborative research agreement between ABBOTT JAPAN Co., Ltd. and Saga University.

**13.4　External contract research organizations**

The secretariat duties involved in this study will be contracted out to Nouvelle Place Inc., an external research organization in accordance with a separately concluded funding protocol. Nouvelle Place Inc. will be responsible for handling all of the office work related to its duties as the research secretariat involved in the promotion and management of the study and will provide various forms of support to Saga University in its role in overseeing and guiding the study, and in ensuring that the research moves forward smoothly. Management of other outside contract organizations will be handled by Saga University or Nouvelle Place Inc.

**13.5　Compensation to cooperative study facilities**

Collaborative study facilities that contribute to this study will receive 10,000 yen as compensation for their cooperation each time web entries are made for the various specified tests and observations at baseline and after 12 and 24 months of treatment or at discontinuation.

**14　Publication of research results, ownership of rights**

**14.1　Clinical study registration**

This study will be registered on UMIN-CTR (http://www.umin.ac.jp/ctr/index-j.htm) for information disclosure. Clinical study registration will be completed before enrollment of the initial patient, and the principal study facility or study secretariat will be responsible for this process.

**14.2** **Publication of study results and ownership of rights**

Matters relating to presentations to academic societies or publications in journals based on the results of this research will be determined after discussion between the principal investigator and the steering committee. The presenter of the study results must undergo review and approval from the principal investigator and the steering committee before publication.

Authors of any publications or presentations to academic societies will follow the author requirements of the International Committee of Medical Journal Editors (authorship)^23^ and the principal investigator will decide accordingly.

After approval from the principal investigator and steering committee, TEIJIN PHARMA LIMITED may use the information from this research (excluding publicly known information) to provide information to medical facilities, and as references when applying for manufacturing approval for drugs or medical devices.

Presentations on research results regarding High-sensitivity Troponin I assay by ABBOTT JAPAN Co., Ltd. and ownership of the results will be as concluded in a contract between ABBOTT JAPAN Co., Ltd. and Saga University.

**14.3　Access rights to the data**

All data collected in this study will belong to the principal investigator. In order to fulfill the needs of this study, those in the data center, research secretariat, and lead statistician will be permitted to access the collected data.

**15　Adherence to study protocol, amendments**

Investigators and others involved in this study will adhere to this study protocol as long as it does not threaten the safety and rights of patients.

**15.1　Study discontinuation or suspension**

(1) The efficacy and safety evaluation committee will consider the appropriateness of continuing the investigation as necessary. If the committee finds that continuation is not appropriate, it will recommend to the principal investigator that the study be discontinued or suspended. If the principal investigator follows the recommendation and decides to terminate the research, the investigators should be notified of this fact and the reason for discontinuation as soon as possible, together with how to deal with the study participants. The study investigators must notify the heads of the participating medical institution of the course of this decision in writing and also report to the institutional review board of each respective facility, and follow the instructions of the respective institutional review boards in dealing with the study participants.

(2) If the Ethics Review Committee of the study site informs or instructs the clinical investigator to discontinue the study, the investigator will promptly report to the principal investigator. After receiving the report, the principal investigator will report to the steering committee to review whether it is appropriate to continue the study (1). If the investigator receives a warning or instructions that the Ethics Review Committee of their institution has decided to suspend or discontinue the study, s/he must immediately report this fact to the head of the facility in writing.

(3) The principal investigator will review whether the study may be continued in the following cases.

1) If vital information relating to quality, safety, and efficacy of the drug used in this study is obtained.

2) If recruiting patients is difficult and the investigator determines reaching the targeted number of subjects is unrealistic.

3) If the objectives of the study are fulfilled before the planned number of patients or study duration has been reached.

4) If the Institutional Review Board of the medical facility in question orders a change in the study protocol, but the principal investigator determines following those instructions would be difficult.

**15.2　Deviations from study protocol**

The investigator must not deviate from or amend the study protocol before obtaining approval from the head of the medical facility based on the institutional review board's preliminary review.

The clinical investigator may, with the prior agreement of the principal investigator and before prior approval from the institutional review board of the study facility, deviate from or change the study protocol if the investigator deems it necessary to avoid emergencies. At that time, the study investigator must promptly notify the principal investigator and institutional review board of the study facility of the deviation or change, its content, and the reason it was made, and whether a revision of the study protocol is necessary, and receive approval from the principal investigator, and the facility head and institutional review board of the study facility. If there is a deviation from the study protocol, the investigator must document the deviation together with the reason why it occurred.

**15.3　Study protocol amendments**

15.3.1 Classification of study protocol amendments

Study protocol amendments will be divided into study amendments and study revisions. Definitions of these classifications will be as follows.

1. Amendment: Partial amendments to the study protocol related to items that might increase the risk to the study participants or partial amendment involving the principal endpoints.

Approval by both the steering committee and the institutional review board are required. Date of approval by the steering committee will be documented on the cover page.

1. Revision: Changes to the study protocol where there is no increase in the risk to study participants and there will be no impact on the primary endpoints.

Institutional review board approval is not required, but the approval of the lead study investigator and a report to the steering committee is required. Review and approval by the institutional review board of the study facility will be up to the individual study facility. Date of approval by the principal investigator should be shown on the cover page

15.3.2 Institutional review board approval of the amendment/revision of the study protocol

If the study protocol or information pamphlet for the patient are amended during the course of this study with the approval of the steering committee, the amended study protocol and information pamphlet must be approved by the institutional review board of the participating study facility. If the change in content is not an amendment but a revision, then each facility should decide for itself if the item requires institutional review board approval.

If approval regarding the revision is obtained from the institutional review board of the study facility, the lead study investigator at each facility should send a copy of the facilty's institutional review board approval document to the research secretariat. The investigator responsible for the study at each facility will retain a copy of the original approval from the institutional review board of the study site.

**16　Research organization**

**16.1　PRIZE study organization**

16.1.1 Principal investigator

Responsible for the conception, planning, execution, analysis, and announcement of this study and for the entire study including management.

Koichi Node, Professor, Department of Cardiovascular Medicine, Saga University

16.1.2 Steering committee

Responsible for the conception, planning, execution, analyses, and announcements of this research; aware of issues associated with the execution of this study and discusses potential solutions; carries out all of the required negotiations to manage this research project.

Chair

Toyoaki Murohara Professor, Department of Cardiology, Nagoya University

Committee member

Teruo Inoue, Professor, Dokkyo Medical University Hospital, Department of Cardiovascular Medicine

Masataka Sata, Professor, Department of Cardiovascular Medicine, Tokushima University

Mitsuru Ohishi, Professor, Department of Cardiovascular Medicine and Hypertension, Kagoshima University Graduate School of Medical and Dental Sciences

16.1.3 Executive (Committee)

Provides advice on the planning and management of this study.

Chair:

Koutarou, Professor, Yokote Clinical Cell Biology and Medicine, Chiba University

Comiittee member:

Kazuomi Kario, Professor, Cardiocascular Medicine, Jichi Medical University

Hirotaka Watada, Professor, Division of Metabolism and Endocrinology, Department of Internal Medicine

Iichirou Shimomura, Professor, Department of Metabolic Medicine, Osaka University

Munehide Matsuhisa, Professor, Therapeutics and Research Center, The University of Tokushima

Yoshihiro Fukumoto, Professor, Department of Cardio-Vascular Medicine, Kurume University

Koji Maemura, Professor, Department of Cardiovascular Medicine, Nagasaki University Hospital

Yusuke Ohya, Professor, Department of Cardiovascular Medicine, Nephology and Neurology University of the Ryukyus

16.1.4 Data safety monitoring board (DSMB)

Assesses safety data investigations into the need for protocol revision, and appropriateness of continuing the study and makes recommendataions to the principal investigator.

Chair

Hiroyuki Daida, Professor, Department of Cardiovascular Medicine, Juntendo University

Members

Junya Ako, Professor, Department of Cardio-angiology, Kitasato University

Kazuo Kitagawa, Associate Professor, Department of Neurology, Osaka University

16.1.5 Researchers responsible for the substudy

Secondary endpoints such as Flow Mediated Dilation (FMD), Pulse Wave Velocity (PWV), Cardio-ankle vascular index (CAVI), IMT, Echocardiography, atherosclerosis index (AI) will all be evaluated.

FMD: Yukihito Higashi, Professor, Research Institute for Radiation Biology and Medicine

PWV/CAVI: Hirofumi Toyama, Professor, Second Department of Internal Medicine, Tokyo Medical University

IMT: Tomoko Ishizu, Lecturer, Department of Cardiovascular Medicine / Clinical Pathology, Tsukuba University

Echocardiography: Hirotsugu Yamada, Lecturer, Department of Cardiovascular Medicine / Echo Center, Tokushima University

AI: Kazuo Eguchi, Lecturer, Cardiovascular Medicine, Jichi Medical Unviersity,

16.1.6 Clinical event committee (CEC)

Responsible for central assessment of events related to the secondary endpoint items.

Committee chairman

Wataru Shimizu, Professor, Department of Cardiovascular Medicine, Nippon Medical School

Committee member

Yoshio Kobayashi, Professor, Department of Cardiovascular Medicine, Chiba University

Masaharu Ishihara, Director, Cardiovascular Medicine, National Cerebral and Cardiovascular Center

16.1.7 IMT measurement central facility

Responsible for the centralized measurement of IMT.

Researcher-in-charge

Tomoko Ishizu, Lecturer, Department of Cardiovascular Medicine / Clinical Pathology, Tsukuba

16.1.8 Statistical analysis committee member

Yasunori Sato, Lecturer, Chiba University Hospital Clinical Research Center

16.1.9 Principal study facility

To ensure that this study runs smoothly, oversees and manages the entire research project.

Department of Cardiovascular Medicine, Saga University

Junichi Oyama

Address: 5-1-1 Nabeshima, Saga 849-8501

TEL: 81-952-34-2364 FAX: 81-952-34-2089

**16.2　Outsource organization**

16.2.1 Research secretariat

Responsible for all of the clerical work related to the promotion and management of this study, provides support to ensure the smooth execution of this research at all participating facilities and by all investigators.

Nouvelle Place Inc.

Address: 1-6-3 Yushima-Iccyoume Building, Yushima, Tokyo 113-0034

TEL: 81-3-3814-1616 FAX: 81-3-3814-3388

16.2.2 Data management

Conducts various forms of data management such as data input and data cleaning.

Data Management

CPOC Co., Ltd

Address: 1-7 East Nihonbashi, Nomura Real Estate East Nihonbashi Building 9th Floor,

Tokyo 103-0004

Phone: 03-4500-9501 (Main number)

FAX: 03-5825-505516.2.3 Audit

16.2.3 Audit

Auditing duties will confirm study operation and data reliability of this research.

Person in charge: Shinichiro Ueda, Director, Clinical Research Support Center, University of the Ryukyus Hospital

Address: 207 Azauehara, Nishihara, Nakagami, Okinawa 903-0215

TEL: 81-98-895-1351

16.2.4 Central laboratory center

Collects samples for measurement at a central laboratory, assays, and prepares results reports.

SRL Inc.

Address: 2-1-1 Nishishinjuku, Tokyo 163-0409

TEL: 81-3-6279-0900

16.2.5 NT-proBNP assay

Roche Diagnostics K.K.

Address: 2-6-1 Shiba, Tokyo 105-0014

TEL: 81-3-5443-7041

16.2.6 High-sensitivity Troponin I measurement

ABBOTT JAPAN CO., LTD.

Address: Sumitomofudousan Sanda Twin Building Nishi-kan, Sanda, Tokyo 108-6305

TEL 81-3-4555-1000

**16.3　Research fee sponsor**

Teijin Pharma Limited.

Address: Kasumigaseki Common Gate West Tower, 2-1, Kasumigaseki 3-chome, Tokyo 100-8585

TEL: 81-3-3506-4822

**17　Amendments of the study protocol**

**Revision 1 (Ver. 1.0 to Ver. 2.0)**

| Revised items | Previous  Study Protocol Ver. 1.0  January 8, 2014 | Revised  Study Protocol Ver. 2.0  September 25, 2014 | Reason for Amendment |
| --- | --- | --- | --- |
| 11.1 | Audit | Monitoring and Audit | Changes in the Monitoring and Audit System |
| 3.2 | Exclusion Criteria:  (12) Other patients who are deemed unsuitable for inclusion in this clinical study based on the investigators' discretion | Exclusion Criteria:  (12) Patients who have had carotid endarterectomy or carotid stent placement  (13) Other patients who are deemed unsuitable for inclusion in this clinical study based on the investigators' discretion | To add exclusion criteria |
| 3.2 | Exclusion Criteria:  [Rationale for these criteria]  (12) This item is included to allow investigators to exclude subjects for reasons that had not been identified as inappropriate for the study during the planning stages. | Exclusion Criteria:  [Rationale for these criteria]  (12) Patient was excluded since it was not possible to obtain the primary endpoint data: a common carotid artery IMT reading.  (13) This item is included to allow investigators to exclude subjects for reasons that had not been identified as inappropriate for the study during the planning stages. |  |
| 5.1 | Observation, Tests, Evaluation Schedule  Acceptable range (Baseline)  Within 2 months of informed consent | Observation, Tests, Evaluation Schedule  Acceptable range (Baseline)  Within 2 months of informed consent^10^  ^10^ However, depending on the conditions at the institution and the patient's preference for timing of next visit, as much as 3 months will be permitted. | To add the following expression |
| 7.2(4) | (4) Urinary L=FABP [in Diabetes or CKD complications] | (4) Urinary L-FABP [in Diabetes or CKD complications] | To revise the expression |
| 9.1 | Submission of case report forms  The investigator will enter the Case Report Form data into the EDC. | Submission of case report forms  The investigator will enter the Case Report Form data into the EDC within roughly 2 months of the patient visit. | To add the following expression |
| 10 | Ethics  This study will be conducted in strict observance of the Declaration of Helsinki and the Ethical Guidelines for Clinical Research from the MHLW. | Ethics  This study will be conducted in strict observance of the most recent Declaration of Helsinki and the Ethical Guidelines for Clinical Research from the MHLW. | To add the following expression |
| 11.1 | Audit  The objective is to confirm that this study is being conducted appropriately and to assure the reliability of the resulting data. The Audit responsibilities of this study will be outsourced to a CRO. In carrying out an audit, any records or medical data that may identify the subject must be kept confidential to maintain patient privacy. | Monitoring and Audit  The objective is to confirm that this study is being conducted appropriately and to assure the reliability of the resulting data. The Monitoring and Audit responsibilities of this study will be outsourced to a CRO. In carrying out the monitoring and audit, all records that may expose the identity of the patient or medical information will be kept confidential to protect patient confidentiality. | Changes in the Monitoring and Audit System |
| 11.2 | Archiving of records  Data will be archived for a period of 3 years after study discontinuation or completion. | Archiving of records  Data will be archived for a period of 5 years after study discontinuation or completion, or for 3 years after the final results have been published, whichever is longer. | Changed in view of ethical guidelines revision |
| 11.3 | Direct access to source documents, etc.  If requested, the investigators and clinical study sites will permit direct inspection of all records related to this study for monitoring or auditing purposes or by the Ethics Review Committee, and cooperate in full. | Direct access to source documents, etc.  If requested, the investigators and clinical study sites will permit direct inspection of all records related to this study for monitoring or auditing purposes or by the Ethics Review Committee, and cooperate in full. | Changes in the Monitoring and Audit System |
| 14.2 | Publication of study results and ownership of rights | Publication of study results and ownership of rights  (Add the following expression at the end.)）  Announcement of research findings and results on NT-proBNP measurement from Roche Diagnostics K.K. will be handled as per the cooperative research agreement signed between Roche Diagnostics K.K. and National University Corporation Saga University on May 29, 2014. | To add the following expression |
| 14.3 | Access rights to data  All data collected in this study will belong to the principal investigator. In order to fulfill the needs of this study, those in the data center, research secretariat, and lead statistician will be permitted to access the collected data. | Access rights to data  All data collected in this study will belong to the principal investigator. In order to fulfill the needs of this study, those who are responsible for auditing duties, monitoring duties, statistical analysis duties or in the research secretariat will be permitted to access the collected data. | Changes in the Monitoring and Audit System |
| 16.1.4 | Data and Safety Monitoring Board (DSMB)  Committee members  Kazuo Kitagawa, Associate Professor, Department of Neurology, Osaka University | Data and Safety Monitoring Board (DSMB)  Committee members  Kazuo Kitagawa, Professor, Department of Neurology, Tokyo Women's Medical University | Due to change in Affiliation |
| 16.1.6 | Clinical Events Committee (CEC)  Committee members  Masaharu Ishihara, Director, Cardiovascular Medicine, National Cerebral and Cardiovascular Center | Clinical Events Committee (CEC)  Committee members  Masaharu Ishihara, Clinical Director, Coronary Heart Disease, Hyogo College of Medicine | Due to change in Affiliation |
| 16.1.9 | Primary Study Site  Department of Cardiovascular Medicine, Saga University  Junichi Oyama | Primary Study Site  Department of Cardiovascular Medicine, Saga University  Junichi Oyama  Ayumu Yamazaki | Addition of a researcher to take on responsibilities |
| 16.2.2 | Data Management  CPOC Co., Ltd  Address: 1-7 East Nihonbashi, Nomura Real Estate East Nihonbashi Building 9th Floor, Tokyo 103-0004  Phone: 03-4500-9501 (Main number)  FAX: 03-5825-5055 | Data Management  Data Science, Mediscience Planning Inc  Address: 3-2 Nihonbashi Hamacho Chuo-ku Tokyo 103-0007 Nihonbashi Hamacho Central Building 3F  Phone 03-5820-7204,  FAX: 03-5825-5055 | Change in Company name and Address |
| 16.2 | Outsource organization  Audit  Auditing duties will confirm study operation and data reliability of this research. | Monitoring  Monitoring duties will be performed to ensure that this study is conducted appropriately | Changes in the Monitoring and Audit System |
| 16.2 | Outsource organization | Add the item below.  Audit  Auditing duties will be performed to ensure the reliability of study results from this research.  Outsource will be determined by the Steering Committee. | Changes in the Monitoring and Audit System |
| 16.2 | Outsource organization | Add the item below.  Inflammation markers (RAGE, ANGPTL2) measured  Department of Cardiovascular Medicine, Saga Medical School, Faculty of Medicine, Saga University  Address: 5-1-1 Nabeshima City, Saga City, Saga Prefecture, 849-8501, JAPAN  Phone 0952-34-2364  FAX: 0952-34-2089 | To add an assay institution |

**Revision 2 (Ver. 2.0 to Ver. 2.1)**

| Revised items | Previous  Study Protocol Ver. 2.0  September 25, 2014 | Revised  Study Protocol Ver. 2.1  April 7, 2015 | Reason for amendment |
| --- | --- | --- | --- |
| 3.2 | (7) Patients with severe renal dysfunction (eGFR <30 mL/min or patients undergoing dialysis) | (7) Patients with severe renal dysfunction (eGFR <30 mL/min/1.73 m^2^ or patients undergoing dialysis) | To correct typo |

**Revision 3 (Ver. 2.1 to Ver. 3.0)**

| Revised items | Previous  Study Protocol Ver. 2.1  April 7, 2015 | Revised  Study Protocol Ver. 3.0  June 5, 2015 | Reason for amendment |
| --- | --- | --- | --- |
| 4.4 | 4.4 Confirmation of Subject Eligibility The investigator will confirm the subject eligibility (3.1, 3.2) of all patients from whom Informed Consent has been obtained.  A carotid duplex test was performed, and the subject is checked for eligibility based on the IMT value for that facility, and enrolled (allocation) if suitable. | 4.4 Confirmation of Subject Eligibility The investigator will confirm the subject eligibility (3.1, 3.2) of all patients from whom Informed Consent has been obtained.  A carotid duplex test was performed and the subject is checked for eligibility based on the IMT value for that facility, and enrolled (allocation) if suitable. Refer to "Confirmation of Subject Eligibility, Enrollment (allocation), Baseline survey, SOP to start investigational treatment" for details. | To ensure consistency in study procedures at all participating sites |
| 4.5.1 | 4.5.1 Enrollment and allocation of patients will be conducted through the registration website. After confirmation of eligibility, the investigator will promptly access the registration website and enter the necessary information and send it. Information sent will immediately checked on the registration website and if appropriate, the patient will be registered into the database as a subject and an investigational treatment will be allotted. If the patient is not appropriate for inclusion, a notice to that effect will be sent out. | 4.5.1 Enrollment and allocation of patients will be conducted through the registration website. After confirmation of eligibility, the investigator will promptly access the registration website and enter the necessary information and send it. Information sent will immediately checked on the registration website and if appropriate, the patient will be registered into the database as a subject and an investigational treatment will be allotted. If the patient is not appropriate for inclusion, a notice to that effect will be sent out. Refer to "Confirmation of Subject Eligibility, Enrollment (allocation), Baseline survey, SOP to start investigational treatment" for details. | To ensure consistency in study procedures at all participating sites |
| 4.6 | 4.6 Baseline studies and start of investigational treatmentThe investigator will conduct a baseline survey within 2 months of obtaining informed consent and if the patient is eligible, investigational treatment will be initiated. The investigator will conduct baseline observations, tests, and evaluation of patients who have been enrolled into this study. In cases where patient ineligibility becomes apparent only after enrollment or if investigational treatment cannot be started for some reason, the study should be terminated in the relevant patient and the reason documented in the survey form.In the febuxostat group, the investigator will prescribe the febuxostat preparation according to the administration method specified in this study and instruct the subject to begin taking the medication. | 4.6 Baseline studies and start of investigational treatmentThe investigator will conduct a baseline survey within 2 months of obtaining informed consent and if the patient is eligible, investigational treatment will be initiated. The investigator will conduct baseline observations, tests, and evaluation of patients who have been enrolled into this study. In cases where patient ineligibility becomes apparent only after enrollment or if investigational treatment cannot be started for some reason, the study should be terminated in the relevant patient and the reason documented in the survey form.In the febuxostat group, the investigator will prescribe the febuxostat preparation according to the administration method specified in this study and instruct the subject to begin taking the medication. Refer to "Confirmation of Subject Eligibility, Enrollment (allocation), Baseline survey, SOP to start investigational treatment" for details. | To ensure consistency in study procedures at all participating sites |
| 5.1  List | 5.1 Observation, tests, and assessment scheduleEligibility assessment○^1^/●^4^ | 5.1 Observation, tests, and assessment scheduleEligibility assessment Baseline(○^1^) ●^4^ | To make tables easier to understand |
| 10.1 | 10.1. Ethics reviewInvestigators will submit all necessary documentation in accordance with the regulations of each facility and receive approval to conduct this study from the Ethics Review Committee of their facility. If the Ethics Review Committee approval is obtained, a copy of the approval will be sent to the Research Secretariat.Annual updates to the Ethics Review Committee approval will be handled as specified at each study site. | 10.1. Ethics reviewInvestigators will submit all necessary documentation in accordance with the regulations of each facility and receive approval to conduct this study from the Ethics Review Committee. If the Ethics Review Committee approval is obtained, a copy of the approval will be sent to the Research Secretariat.Annual updates to the Ethics Review Committee approval will be handled as specified at each study site. | To ensure consistency in study procedures at all participating sites |
| 15.1  (2)  (3) | (2) If the Ethics Review Committee of the study site informs or instructs the clinical investigator to discontinue the study, the investigator will promptly report to the principal investigator. After receiving the report, the principal investigator will report to the steering committee to review whether it is appropriate to continue the study (1). If the investigator receives a warning or instructions that the Ethics Review Committee of their institution has decided to suspend or discontinue the study, s/he must immediately report this fact to the head of the facility in writing.(3) The principal investigator will review whether the study may be continued in the following cases.1) If vital information relating to quality, safety, and efficacy of the drug used in this study is obtained | (2) If the Ethics Review Committee of the study site informs or instructs the clinical investigator to discontinue the study, the investigator will promptly report to the principal investigator. After receiving the report, the principal investigator will report to the steering committee to review whether it is appropriate to continue the study (3). If the investigator receives a warning or instructions that the Ethics Review Committee of their institution has decided to suspend or discontinue the study, s/he must immediately report this fact to the head of the facility in writing.(3) The principal investigator and Steering Committee will review whether the study may be continued in the following cases.1) If vital information relating to study quality, safety, and efficacy is obtained | To add a role to the Steering Committee |
| 16.2.3 | Shinichiro Ueda, Director, Clinical Research Support Center, University of the Ryukyus HospitalAddress: 207 Azakamihara, Nishihara-cho, Nakagami-gun, Okinawa-ken 903-0215Phone: 098-895-1351 | Shinichiro Ueda, Director, Clinical Trials and Management Center, University of the Ryukyus HospitalAddress: 207 Azakamihara, Nishihara-cho, Nakagami-gun, Okinawa-ken 903-0215Phone: 098-895-1351 | Due to change in Affiliation |
| 16.2.4 | 16.2.4 AuditAuditing duties will be performed to ensure the reliability of study results from this research.Outsource vendor will be determined by the Steering Committee. | 16.2.4 AuditAuditing duties will be performed to ensure the reliability of study results from this research.Clinical Research Support Center, University of the Ryukuus HospitalAddress: 207 Azakamihara, Nishihara-cho, Nakagami-gun, Okinawa-ken 903-0215Phone: 098-895-1351 | Subcontractor decided |

**Revision 4 (Ver. 3.0 to Ver. 4.0)**

| Revised items | Previous  Study Protocol Ver.3.0 June 5, 2015 | Revised  Study Protocol Ver.4.0 October 1, 2015 | Reason for amendment |
| --- | --- | --- | --- |
| 0.6 | (2) Treatment observation period: From date of approval by the Clinical Research Ethics Review Committee to March 2018 (Enrollment period: January 2016) | (2) Treatment Observation period: From date of approval by the Clinical Research Ethics Review Committee to August 2018 (Enrollment period: June 2016. However, the test treatment must be initiated before the end of the enrollment period) | The Steering Committee decided to extend the enrollment period |
| 4.1.1 | (2) Treatment observation period: From date of approval by the Clinical Research Ethics Review Committee to March 2018 (Enrollment period: January 2016) | (2) Treatment Observation period: From date of approval by the Clinical Research Ethics Review Committee to August 2018 (Enrollment period: June 2016. However, the test treatment must be initiated before the end of the enrollment period) | The Steering Committee decided to extend the enrollment period |

**Revision 5 (Ver. 4.0 to Ver. 4.1)**

| Revised items | Previous  Study Protocol Ver.4.0  October 1, 2015 | Revised  Study Protocol Ver.4.1  January 18, 2016 | Reason for amendment |
| --- | --- | --- | --- |
| 16.1.9 | 16.1.9 Primary Study Site Will provide overall oversight to promote the smooth operation of this study.  Department of Cardiovascular Medicine, Saga Medical School, Faculty of Medicine, Saga University  Junichi Oyama  Ayumu Yamazaki  Address: 5-1-1 Nabeshima City, Saga City, Saga Prefecture, 849-8501, JAPAN  Phone: 0952-34-2364 FAX: 0952-34-2089 | 16.1.9 Primary Study Site Will provide overall oversight to promote the smooth operation of this study.  Department of Cardiovascular Medicine, Saga Medical School, Faculty of Medicine, Saga University  Junichi Oyama  Ayumu Yamazaki  Atsushi Tanaka  Address: 5-1-1 Nabeshima City, Saga City, Saga Prefecture, 849-8501, JAPAN  Phone: 0952-34-2364 FAX: 0952-34-2089 | Due to change in Person in charge |

**Revision 6 (Ver. 4.1 to Ver. 5.0)**

| Revised items | Previous  4.1  January 18, 2016 | Revised  5.0  February 15, 2016 | Reason for amendment |
| --- | --- | --- | --- |
| 7.2 Secondary endpoints | (3) The following measurements, amount of change, and percentage change in cardiovascular function tests from baseline at baseline and after 12 and 24 months of treatment. (optional items)  Flow Mediated Dilation (FMD), Pulse Wave Velocity (PWV), Cardio-ankle vascular index (CAVI), IMT, Echocardiography (systolic, diastolic function, left atrial diameter, left ventricular weight coefficient, atherosclerosis index (AI)(4) Amount of change and percentage change from baseline for the following clinical laboratory test items at baseline, and after 6 months, 12 months, and 24 months of treatment. Serum Lipids (TC, HDL-C, TG, LDL-C [indirect method], non-HDL-C), serum creatinine, eGFR(converted), urinary albumin excretion (corrected for creatinine) [In case of diabetes complication], urinary L-FABP [diabetes or CKD complication](5) Values, amount of change, and percentage change in the following clinical laboratory tests and cardiovascular function tests at baseline, after 12 months, and after 24 months of treatment. (* is optional) NT-proBNP, blood pressure (outpatient)(6) All following events that occur from baseline until after 24 months of treatment or study discontinuation. Onset of complex events (cardiovascular death, non-fatal myocardial infarction and stroke, renal event, total deaths) and each event | (3) Deleted (moved to exploratory items)(3) Amount of change and percentage change from baseline for the following clinical laboratory test items at baseline, and after 6 months, 12 months, and 24 months of treatment. Serum Lipids (TC, HDL-C, TG, LDL-C [indirect method], non-HDL-C), serum creatinine, eGFR (converted), urinary albumin excretion (corrected for creatinine) [In case of diabetes complication], urinary L-FABP [diabetes or CKD complication](4) Values, amount of change, and percentage change in the following clinical laboratory tests and cardiovascular function tests at baseline, after 12 months, and after 24 months of treatment. (* is optional) NT-proBNP, blood pressure (outpatient)(5) All following events that occur from baseline until after 24 months of treatment or study discontinuation. Onset of complex events (cardiovascular death, non-fatal myocardial infarction and stroke, renal event, total deaths) and each event(6) All adverse events that occur from baseline until after 24 months of treatment or study discontinuation. Appearance of Adverse events that need to be evaluated (6.2) will be reviewed for safety. | Since these are optional, to change to exploratory endpoints |
| 7.3 Exploratory Evaluation Item | (1) The following measurements, amount of change, and percentage change from baseline in clinical laboratory tests and cardiovascular function tests at baseline and after 12 and 24 months of treatment. (* are optional measurement items)High sensitivity CRP, 1,5AG, smaller LDL particle, RLP-C, MDA-LDL, serum cystatin C, RAGE, high molecular weight adiponectin, high sensitivity troponin I, ANGPTL2, blood pressure (home)* | (1) The following measurements, amount of change, and percentage change from baseline in clinical laboratory tests and cardiovascular function tests at baseline and after 12 and 24 months of treatment. (* are optional measurement items)High sensitivity CRP, 1,5AG, smaller LDL particle, RLP-C, MDA-LDL, serum cystatin C, RAGE, high molecular weight adiponectin, high sensitivity troponin I, ANGPTL2, blood pressure (home)*, Flow Mediated Dilation*(FMD), Pulse Wave Velocity* (PWV), Cardio Ankle Vascular Index* (CAVI), echocardiography (Systolic, diastolic function, left atrial diameter, left ventricular weight coefficient), atherosclerosis indicator(AI) | Since these are optional, to change to exploratory endpoints |
| 16.1.5  Researcher in Charge of Substudy | Secondary endpoints such as Flow Mediated Dilation (FMD), Pulse Wave Velocity (PWV), Cardio-ankle vascular index (CAVI), IMT, Echocardiography, atherosclerosis index (AI) will all be evaluated. | Secondary endpoints and exploratory endpoints such as Flow Mediated Dilation (FMD), Pulse Wave Velocity (PWV), Cardio-ankle vascular index (CAVI), IMT, echocardiography, atherosclerosis index (AI) will all be evaluated. | Since these are optional, to change to exploratory endpoints |

**Revision 7 (Ver. 5.0 to Ver. 5.1)**

| Revised items | Previous  Study Protocol Ver.5.0  February 15, 2016 | Revised  Study Protocol Ver. 5.1  August 1, 2016 | Reason for amendment |
| --- | --- | --- | --- |
| 6.3.1 Emergency Report | 6.3.1 Emergency report If any serious adverse events (6.2.3) should occur, they will be subject to an emergency report. If the relevant adverse event is observed, the investigator will immediately notify the head of the research facility. When the investigator is made aware of the relevant adverse event, s/he will immediately document the required information in the adverse event report and fax it to the Research Secretariat at (03-3814-3388). | 6.3.1 Emergency ReportIf any serious adverse events (6.2.3) should occur, they will be subject to an emergency report. If the relevant adverse event is observed, the investigator will immediately notify the head of the research facility. When the investigator is made aware of the relevant adverse event, s/he will immediately document the required information in the adverse event report and fax it to the Research Secretariat at (03-6777-0033). | Change in Contact information due to relocation |
| 16.1.9 Primary Study Site | 16.1.9 Primary Study Site Will provide overall oversight to promote the smooth operation of this study.  Department of Cardiovascular Medicine, Saga Medical School, Faculty of Medicine, Saga University  Junichi Oyama  Ayumu Yamazaki  Atsushi Tanaka | 16.1.9 Primary Study Site Will provide oversight to promote the smooth operation of this study.  Department of Cardiovascular Medicine, Saga Medical School, Faculty of Medicine, Saga University  Junichi Oyama  Ayumu Yajima  Atsushi Tanaka | For name change |
| 16.2.1 Research Secretariat | 16.2.1 Research Secretariat To promote the operation of this study, this group will be responsible for all administrative duties and provide support so that participating study sites and participating investigators will be able to conduct their research smoothly.  Nouvelle Place Inc.  Address: 1-6-3 Yushima, Tokyo, Yushima 1-chome Building, 113 - 0034  Phone03-3814-1616 FAX: 03-3814-3388 | 16.2.1 Research Secretariat To promote the operation of this study, this group will be responsible for all administrative duties and provide support so that participating study sites and participating investigators will be able to conduct their research smoothly.  Nouvelle Place Inc.  Address: Toranomon Towers Office 4-1-28 Toranomon Tokyo 105-0001  Phone: 03-6680-2525 FAX: 03-6777-0033 | Change in Contact information due to relocation |

**Revision 8 (Ver. 5.1 to Ver. 5.2)**

| Revision  Item | Previous  Study Protocol Version 5.1  September 1, 2016 | Revised  Study Protocol Ver.5.2  December 12, 2016 | Reason for Amendment |
| --- | --- | --- | --- |
| 0.5.3  Exploratory Evaluation Item | 3 Exploratory Evaluation Item(1) The following measurements, amount of change, and percentage change from baseline in clinical laboratory tests and cardiovascular function tests at baseline and after 12 and 24 months of treatment. (* are optional measurement items) High sensitivity CRP, 1,5AG, smaller LDL particle, RLP-C, MDA-LDL, serum cystatin C, RAGE, high molecular weight adiponectin, high sensitivity troponin I, ANGPTL2, blood pressure (home)* | 3 Exploratory Evaluation Item(1) The following measurements, amount of change, and percentage change from baseline in clinical laboratory tests and cardiovascular function tests at baseline and after 12 and 24 months of treatment. (* are optional measurement items) High sensitivity CRP, 1,5AG, smaller LDL particle, RLP-C, MDA-LDL, serum cystatin C, RAGE, high molecular weight adiponectin, high sensitivity troponin I, ANGPTL2, blood pressure (home)*, FGF23, PCSK9, Alb, Ca, P | New measurement added |
| 5  Observation, Tests, Evaluation | 5.1 Observation, Tests, Evaluation Schedule 9 Special Blood biochemistry tests  (NT-proBNP, High sensitivity CRP, 1,5AG, smaller LDL particle, RLP-C, MDA-LDL, serum cystatin C, RAGE, high molecular weight adiponectin, high sensitivity Troponin I, AGPTL2) | 5.1 Observation, Tests, Evaluation Schedule 9 Special Blood biochemistry tests  (NT-proBNP, High sensitivity CRP, 1,5AG, smaller LDL particle, RLP-C, MDA-LDL, serum cystatin C, RAGE, high molecular weight adiponectin, high sensitivity Troponin I, AGPTL2, FGF23, PCSK9, Alb, Ca, P) | New measurement added |
| 5  Observation, Tests, Evaluation | 5.2.12 Measurement of Clinical Laboratory Tests (endpoints) at a Central Testing Facility(1) Special Blood Biochemistry TestsNT-proBNP, high sensitivity CRP, 1,5AG, LDL, RLP-C, MDA-LDL, serum cystatin C, RAGE, high molecular weight adiponectin, high sensitivity Troponin I, ANGPTL2.Measured at a central testing facility at baseline, and after 12, and 24 months of treatment (or after study discontinuation). | 5.2.12 Measurement of Clinical Laboratory Tests (endpoints) at a Central Testing Facility(1) Special Blood Biochemistry TestNT-proBNP, high sensitivity CRP, 1,5AG, smaller LDL particle, RLP-C, MDA-LDL, serum cystatin C, RAGE, high molecular weight adiponectin, high sensitivity troponin I, ANGPTL2, FGF23, PCSK9, Alb, Ca, PMeasured at a central testing facility at baseline, and after 12, and 24 months of treatment (or after study discontinuation). | New measurement added |
| 7  Evaluation  Item | 3 Exploratory Evaluation Item(1) The following measurements, amount of change, and percentage change from baseline in clinical laboratory tests and cardiovascular function tests at baseline and after 12 and 24 months of treatment. (* are optional measurement items)High sensitivity CRP, 1,5AG, smaller LDL particle, RLP-C, MDA-LDL, serum cystatin C, RAGE, high molecular weight adiponectin, high sensitivity troponin I, ANGPTL2, blood pressure (home)*, Flow Mediated Dilation*(FMD), Pulse Wave Velocity* (PWV), Cardio Ankle Vascular Index* (CAVI), echocardiography* (systolic, diastolic function, left atrial diameter, left ventricular weight coefficient), atherosclerosis indicator* (AI). | 3 Exploratory Evaluation Item(1) The following measurements, amount of change, and percentage change from baseline in clinical laboratory tests and cardiovascular function tests at baseline and after 12 and 24 months of treatment. (* are optional measurement items)High sensitivity CRP, 1,5AG, smaller LDL particle, RLP-C, MDA-LDL, serum cystatin C, RAGE, high molecular weight adiponectin, high sensitivity troponin I, ANGPTL2, blood pressure (home)*, Flow Mediated Dilation*(FMD), Pulse Wave Velocity* (PWV), Cardio Ankle Vascular Index* (CAVI), echocardiography* (systolic, diastolic function, left atrial diameter, left ventricular weight coefficient), atherosclerosis indicator* (AI), FGF23, PCSK9, Alb, Ca, P | New measurement added |
| 16  Research  Organization |  | 16.2.9 Alb, Ca, FGF23, P measurementsCardiology, Osaka Medical CollegeAddress: 2-7 Daigakumachi, Takatsuki, Osaka 569-0801 JapanPhone: 072-683-122116.2.10 PCSK9 measurementBML, Inc.Address: 21-3-5, Sendagaya, Tokyo 151-0051Phone: 03-3350-0111 (Main) | New measurement added |

**Revision 9 (Ver. 5.2 to Ver. 5.3)**

| Revised items | <Old>  Study Protocol Ver.5.2  December 12, 2016 | <Revised>  Study Protocol Ver.5.3  September 12, 2018 | Reason for Amendment  Reason |
| --- | --- | --- | --- |
| 0 Summary | 0.5.2 Secondary endpoints  (3) The following measurements, amount of change, and percentage change in cardiovascular function tests from baseline at baseline and after 12 and 24 months of treatment. (optional items)  Flow Mediated Dilation (FMD), Pulse Wave Velocity (PWV), Cardio-ankle vascular index (CAVI), IMT, Echocardiography (systolic, diastolic function, left atrial diameter, left ventricular weight coefficient, atherosclerosis index (AI) | 0.5.2 Secondary endpoints  (3) Deleted (moved to exploratory items) | Changed to exploratory endpoints since they are optional |
| 0 Summary | 0.5.3 Exploratory Evaluation Item  (1) Values, amount of change, and percentage change in the following clinical Laboratory test and cardiovascular function tests at baseline, after 12 months, and after 24 months of treatment. (tests marked * are optional)  High sensitivity CRP, 1,5AG, smaller particle LDL, RLP-C, MDA-LDL, serum cystatin C, RAGE, high molecular weight adiponectin, high sensitivity troponin I, ANGPTL2, blood pressure (home)*, FGF23, PCSK9, Alb, Ca, P | 0.5.3 Exploratory Evaluation Item  (1) Values, amount of change, and percentage change in the following clinical laboratory tests and cardiovascular function tests at baseline, after 12 months, and after 24 months of treatment. (* is optional)  High sensitivity CRP, 1,5AG, smaller LDL particle, RLP-C, MDA-LDL, serum cystatin C, RAGE, high molecular weight adiponectin, high sensitivity troponin I, ANGPTL2, blood pressure (home)*, Flow Mediated Dilation*(FMD), Pulse Wave Velocity* (PWV), Cardio Ankle Vascular Index* (CAVI), echocardiography (Systolic, diastolic function, left atrial diameter, left ventricular weight coefficient), atherosclerosis indicator(AI), FGF23, PCSK9, Alb, Ca, P | Since these are optional, they have been changed to exploratory endpoints |
| 1 Background | Hyperuricemia has been suggested as a risk factor for atherosclerosis along with hypertension, hyperlipidemia, and diabetes mellitus, and studies into the relationship between vascular endothelial dysfunction and uric acid are ongoing. | Hyperuricemia has been suggested as a risk factor for atherosclerosis along with hypertension, dyslipidemia, and diabetes mellitus, and studies into the relationship between vascular endothelial dysfunction and uric acid are ongoing. | Ensure language meets that used in guidelines |
| 16 Clinical Research Organization | 16.1.5 Researcher in Charge of Substudy  AI Kazuo Eguchi, Lecturer Cardiovascular Medicine, Jichi Medical University | 16.1.5 Researcher in Charge of Substudy  AI Kazuo Eguchi, Head of Internal Medicine, Hanyu General Hospital | Change in Affiliation |
| 16 Clinical Research Organization | 16.1.8 Statistical Analysis Committee Members  Yasunori Sato, Lecturer, Chiba University Hospital Clinical Research Center | 16.1.8 Statistical Analysis Committee Members  Yasunori Sato, Associate Professor, Keio University School of Medicine | Change in Affiliation |
| 16 Clinical Research Organization | 16.2.4  Clinical Research Support Center, University Hospital, School of Medicine, University of the Ryukyus | 16.2.4  Person in Charge: Katsunori Nakamura Vice director, Clinical Research Support Center, University Hospital, School of Medicine, University of the Ryukyus | Person responsible was selected |

**Revision 10 (Ver. 5.3 to Ver. 5.4)**

| Revised items | <Old>  Study Protocol Version 5.3  September 12, 2018 | <Revised>  Study Protocol Version 5.4  September 10, 2018 | Reason for Amendment  Reason |
| --- | --- | --- | --- |
| 5 Observations, Exams, Assessments | 5.1  ^10^ However, depending on the conditions at the institution and the patient's preference for timing of next visit, as much as 3 months will be permitted. | 5.1  ^10^ Depending on the conditions at the institution and the patient's preference for timing of next visit, as much as 3 months prior to and after consent will be permitted.  <Additions>  ^11^ Depending on the situation at the institution or patient preference regarding next hospital visit, allow this to be obtained up to 90 days before or after the specified date.  ^12^ Depending on the situation at the institution or patient preference regarding next hospital visit, allow this to be obtained up to 90 days before or 180 days of the specified date.  ^13^ Depending on the situation at the institution or patient preference regarding next hospital visit, allow this to be obtained up to 180 days before or after the specified date.  ^14^ Depending on the situation at the institution or patient preference regarding next hospital visit, allow this to be obtained up to 360 days before informed consent.  Final version of schedule is shown below table | Additional notations to account for change in allowance duration. |

| Time point  Item | Eligibility assessment | Baseline^2^ | During study treatment | | |
| --- | --- | --- | --- | --- | --- |
| Visit | - | 1 | 2 | 3 | (After study conclusion) or At discontinuation |
| Course (with baseline as starting point) | Registration | 0 weeks | 6 months | 12 months | 24 months or discontinuation |
| Acceptable range | After informed consent is obtained to before enrollment1^1^ | Within 2 months of informed consent^10^ | ±2 months^11^ | ±2 months^12^ | Date of completion ±2months,  at discontinuation or as promptly as possible^13^ |
| Patient characteristics | ○ |  |  |  |  |
| Use of concomitant therapy^3^ | ○ | ○ | ○ | ○ | ○ |
| Febuxostat administration conditions (febuxostat group alone) |  | ○^2^ | ○ | ○ | ○ |
| Height (baseline alone), body weight |  | ○ |  | ○ | ○ |
| Blood pressure, pulse |  | ○ | △ | ○ | ○ |
| Home-based blood pressure |  | △ | △ | △ | △ |
| Carotid artery ultrasound, common carotid artery, bulbar, internal carotid artery (IMT is centrally measured) | (○^1^) | ●^4,14^ |  | ● | ● |
| Cardiovascular function test ^5^ |  | △^14^ |  | △ | △ |
| Appearance of adverse events |  |  |  |  |  |
| Appearance of events^6^ |  |  |  |  |  |
| Clinical laboratory test^7^: hematology (general hematology, blood biochemistry) | ○^1^ | ○ | △ | ○ | ○ |
| Clinical laboratory tests (test items)^8^: serum uric acid, serum lipids, serum creatinine (eGFR), urinary albumin excretion, urinary L-FABP | ○^1^ | ○ | ○ | ○ | ○ |
| Special blood biochemistry tests measured at core laboratoty^9^ |  | ● |  | ● | ● |
| Blood collection volume (mL) to determine median values | - | 12 |  | 12 | 12 |

**Final version of observation, tests, and assessment schedule**

**References**

1. Ishizaka N, Ishizaka Y, Toda E, Nagai R, Yamakado M. Association between serum uric acid, metabolic syndrome, and carotid atherosclerosis in Japanese individuals. *Arterioscler Thromb Vasc Biol*. 2005;25(5):1038-44. doi:10.1161/01.ATV.0000161274.87407.26

2. Tomiyama H, Higashi Y, Takase B, et al. Relationships among hyperuricemia, metabolic syndrome, and endothelial function. *Am J Hypertens*. 2011;24(7):770-4. doi:10.1038/ajh.2011.55

3. Takayama S, Kawamoto R, Kusunoki T, Abe M, Onji M. Uric acid is an independent risk factor for carotid atherosclerosis in a Japanese elderly population without metabolic syndrome. *Cardiovasc Diabetol*. 2012;11:2. doi:10.1186/1475-2840-11-2

4. Puddu P, Puddu GM, Cravero E, Vizioli L, Muscari A. Relationships among hyperuricemia, endothelial dysfunction and cardiovascular disease: molecular mechanisms and clinical implications. *J Cardiol*. 2012;59(3):235-42. doi:10.1016/j.jjcc.2012.01.013. Epub 2012 Mar 6

5. The Japanese Circulation Society, The Japan Society for Transplantation, The Japanese Association for Thoracic Surgery, The Japanese Society of Hypertension, Japanese Society of Pediatric Cardiology and Cardiac Surgery, The Japanese Society for Cardiovascular Surgery, et al; Guidelines for Treatment of Chronic Heart Failure(JCS 2010) and Guidelines for the Diagnosis and Treatment of Cardiovascular disease(JCS 2009) [cited 2011 June 3]. Available from: http://www.j-circ.or.jp/guideline/index.htm

6. Higgins P, Dawson J, Lees KR, McArthur K, Quinn TJ, Walters MR. Xanthine oxidase inhibition for the treatment of cardiovascular disease: a systematic review and meta-analysis. *Cardiovasc Ther*. 2012;30(4):217-26. doi:10.1111/j.1755-5922.2011.00277.x

7. Salonen R, Nyyssönen K, Porkkala E, et al. Kuopio Atherosclerosis Prevention Study (KAPS). A population-based primary preventive trial of the effect of LDL lowering on atherosclerotic progression in carotid and femoral arteries. *Circulation*. 1995;92(7):1758-64.

8. Ikeda K, Takahashi T, Yamada H, et al. Effect of intensive statin therapy on regression of carotid intima-media thickness in patients with subclinical carotid atherosclerosis (a prospective, randomized trial: PEACE (Pitavastatin Evaluation of Atherosclerosis Regression by Intensive Cholesterol-lowering Therapy) study). *Eur J Prev Cardiol*. 2013;20(6):1069-79. doi:10.1177/2047487312451539

9. Matsushima Y, Takase B, Uehata A, et al. Comparative predictive and diagnostic value of flow-mediated vasodilation in the brachial artery and intima media thickness of the carotid artery for assessment of coronary artery disease severity. *Int J Cardiol*. 2007;117(2):165-72. doi: 10.1016/j.ijcard.2006.04.063

10. Kobayashi K, Akishita M, Yu W, Hashimoto M, Ohni M, Toba K. Interrelationship between non-invasive measurements of atherosclerosis: flow-mediated dilation of brachial artery, carotid intima-media thickness and pulse wave velocity. *Atherosclerosis*. 2004;173(1):13-8. doi: 10.1016/j.atherosclerosis.2003.10.013

11. Hashimoto M, Eto M, Akishita M, et al. Correlation between flow-mediated vasodilatation of the brachial artery and intima-media thickness in the carotid artery in men. *Arterioscler Thromb Vasc Biol*. 1999;19(11):2795-800.

12. Teijin Pharma Limited. Interview Form: Feburic®. 2011 May. 31-3 p.

13. Malik UZ, Hundley NJ, Romero G, et al. Febuxostat inhibition of endothelial-bound XO: implications for targeting vascular ROS production. *Free Radic Biol Med*. 2011;51(1):179-84. doi:10.1016/j.freeradbiomed.2011.04.004

14. Okamoto K, Eger BT, Nishino T, Kondo S, Pai EF, Nishino T. An extremely potent inhibitor of xanthine oxidoreductase. Crystal structure of the enzyme-inhibitor complex and mechanism of inhibition. *J Biol Chem*. 2003;278(3):1848-55. doi:10.1074/jbc.M208307200

15. Kamatani N, Fujimori S, Hada T, et al. An allopurinol-controlled, randomized, double-dummy, double-blind, parallel between-group, comparative study of febuxostat (TMX-67), a non-purine-selective inhibitor of xanthine oxidase, in patients with hyperuricemia including those with gout in Japan: phase 3 clinical study. *J Clin Rheumatol*. 2011;17(4 Supple 2):S13-8. doi: 10.1097/RHU.0b013e31821d36cc

16. Sezai A, Soma M, Nakata KI, et al. Comparison of Febuxostat and Allopurinol for Hyperuricemia in Cardiac Surgery Patients (NU-FLASH Trial). *Circ J*. 2013;77(8):2043-9.

17. Guideline for the management of hyperuricemia and gou. Version 2. Osaka: Medical Review Co., Ltd.; 2010.

18. Japan Atherosclerosis Society. Guidelines for the management of Dyslipidemias to prevent atherosclerotic diseases 2013 edition. Tokyo: Kyorinsha; 2013.

19. Oyama J, Ishizu T, Sato Y, et al. Rationale and design of a study to evaluate the effects of sitagliptin on atherosclerosis in patients with diabetes mellitus: PROLOGUE study. Int J Cardiol. 2014;174(2):383-4. doi: 10.1016/j.ijcard.2014.03.204

20. Crouse JR 3rd, Raichlen JS, Riley WA, et al; METEOR Study Group. Effect of rosuvastatin on progression of carotid intima-media thickness in low-risk individuals with subclinical atherosclerosis: the METEOR Trial. *JAMA*. 2007;297(12):1344-53. doi:10.1001/jama.297.12.1344

21. Kawai T, Ohishi M, Takeya Y, et al. Serum uric acid is an independent risk factor for cardiovascular disease and mortality in hypertensive patients. *Hypertens Res*. 2012;35(11):1087-92. doi:10.1038/hr.2012.99

22. Tomiyama H, Ishizu T, Kohro T,et al. Longitudinal association among endothelial function, arterial stiffness and subclinical organ damage in hypertension. *Int J Cardiol*. 2018;253:161–166. doi: 10.1016/j.ijcard.2017.11.022

23. International Committee of Medical Journal Editors [Internet]. Uniform Requirements for Manuscripts Submitted to Biomedical Journals: Writing and Editing for Biomedical Publications [updated 2010 April]. Available from: http://www.icmje.org
