# Supplementary material for: Febuxostat does not delay progression of carotid atherosclerosis in patients with asymptomatic hyperuricemia: A randomized, controlled trial
Source: PLoS Med. 2020 Apr 22;17(4):e1003095. doi: 10.1371/journal.pmed.1003095 (PMC7176100; doi:10.1371/journal.pmed.1003095)
Supplement: S2 Text — (DOCX) [file pmed.1003095.s003.docx]

**S2 Text.**

**Event evaluation statement**

**1 Cardiovascular death**

**1-1. Sudden cardiac death**

1) If the patient dies within 1 hour of presenting with serious chest symptoms, or if the patient
 dies at almost the same time as symptoms appear.

2) If the patient dies within 24 hours of the appearance of symptoms and no clear cause other than
 cerebrovascular or cardiovascular disease can be observed.

3) Other cases where cardiovascular disease is identified upon autopsy or other procedures.

**1-2. Fatal myocardial infarction**

Death associated with myocardial infarction that satisfies the criteria listed below in section 2.

**1-3. Fatal stroke**

Death associated with stroke that satisfies the criteria listed below in section 3.

**2. Non-fatal myocardial infarction**

Patients who develop symptoms associated with myocardial infarction that satisfy the criteria listed below and survive.

**[Assessment criteria for myocardial infarction]**

Two or more of the following 3 are positive for myocardial infarction: symptoms, blood biochemistry,
 ECG findings

[1] Symptoms (at least 1 of the following is present)

- Chest pain lasting for 30 minutes or longer

- Pulmonary edema

- Cardiogenic shock with no other causes

[2] Blood biochemistry criteria (increase in at least one of the following myocardial markers)

- CK or CK-MB ≥2-fold upper limit of normal

- Rapid Troponin-T or H-FABP tests are positive

[3] ECG findings, coronary angiography (findings indicative of myocardial infarction) (at least 1 of
 the above apply)

- New Q wave appears, or ST elevation is seen in at least 2 of the above leads

- Left bundle branch block, ischemic ST-T abnormality

- Coronary angiography reveals significant narrowing or occlusion of coronary arteries

**3. Non-fatal stroke**

Patient survived stroke that satisfies the following criteria

**[Evaluation criteria for stroke]**

- If new neurological signs appear and the source lesion can be identified on various imaging studies

- If a definitive diagnosis can be confirmed through various imaging studies (head MRI/CT or
 angiography)

**3-1. Cerebral infarction**

Types of cerebral infarctions are classified according to the TOAST disease classification (1993). When the CT is performed during head imaging, even cases where new abnormalities that could explain the neurological signs are not observed, should be included in events. If an MRI is performed, an event should only be recorded if an infarct lesion can be confirmed.

1) Atheroma cerebral infarction

No emboligenic heart disease* is identified on an ultrasound or angiography test (DSA or MRA, carotid duplex, TTE, ECG), and arteriosclerotic narrowing of more than 50% is observed in intracranial or extracranial arteries.

2) Cardioembolic stroke

More than 1 emboligenic heart disease is identified on an ultrasound or angiography test (DSA or MRA, carotid duplex, TTE, ECG) and arteriosclerotic narrowing of more than 50% is not observed in intracranial or extracranial arteries.

3) Lacunar infarction

[1] No emboligenic heart disease* is identified on an ultrasound or angiography test (DSA or MRA, carotid duplex, TTE, ECG) and arteriosclerotic narrowing of more than 50% is not observed in intracranial or extracranial arteries.

[2] No other diseases or conditions that could account for cerebral infarction** are present.

[3] Head MRI/CT is normal or infarct lesions <15 mm are present in the brain stem or cerebral
 subcortex.

[4] Cortical signs are not observed clinically.

4) Cerebral infarction for other reasons

[1] No emboligenic heart disease* is identified on an ultrasound or angiography test (DSA or MRA, carotid duplex, TTE, ECG) and arteriosclerotic narrowing of more than 50% is not observed in intracranial or extracranial arteries.

[2] ** are possible causes of cerebral infarction other than 1) to 3) above

5) Cerebral infarctions other than 1) to 4) cannot be classified.

Cerebral infarctions where investigations are insufficient or searching has been sufficient, but lesion fails to fall into any of the above categories, or cerebral infarction is of unknown cause

* emboligenic heart disease:

(high risk)

mechanical valve, mitral valve stenosis with atrial fibrillation, atrial fibrillation (excluding

afib alone), left atrium thrombus, left atrial appendage thrombus, sick sinus syndrome, fresh

myocardial infarction (<4 weeks post-MI), left intraventricular thrombus, dilated

cardiomyopathy, partial

left ventricular wall asystole, left atrial myxoma, infectious endocarditis

(intermediate risk)

prolapsed mitral valve, mitral valve annulus calcification, mitral valve stenosis without atrial

fibrillation, interartrial smoke-like echo, interatrial septal aneurysm, foramen ovale patent,

atrial flutter, atrial fibrillation alone, bioprosthetic valve, nonbacterial thrombotic endocarditis,

congenital heart disease, partial hypokinesis of the left ventricular wall, over 4 weeks since

onset <6 months since myocardial infarction

** Other reasons

Artery dissection, Moyamoya disease, fibromuscular dysplasia (FMD), vasculitis (includes meningitis), pulmonary arteriovenous fistula, antiphospholipid antibody syndrome, coagulative disorder

**3-2. Cerebral hemorrhage**

　[1] Onset of neurological signs

　[2] Head MRI/CT reveals signs of cerebral, cerebellar, or brain stem hematoma or scars and the lesion

　　　distribution agrees with the neurological signs of the patient. However, hemorrhagic infarctions and 　　
　　　　intratumoral hemorrhage will not be included under cerebral hemorrhages, but rather, hemorrhagic 　　　　　
　　　　infarctions will be classified as cerebral infarctions.

**3-3.　Subarachnoid hemorrhage**

[1] Appearance of sudden headache, impaired consciousness (localized neurological signs do not have 　　
　　　　　to appear).

[2] New hematoma present in the subarachnoid space on head MRI/CT. If it cannot be confirmed on
　　　　　head MRI/CT, a cerebrospinal fluid exam reveals bloody CSF.

**3-4. Stroke of unknown disease type**

Head MRI/CT was not performed, or it is unknown if patient falls into cerebral infarction, cerebral hemorrhage or subarachnoid hemorrhage category.

**4. Renal events**

Doubling of serum creatinine, initiation of hemodialysis, renal transplant

**5. All-cause deaths**
