# Supplementary material for: Febuxostat does not delay progression of carotid atherosclerosis in patients with asymptomatic hyperuricemia: A randomized, controlled trial
Source: PLoS Med. 2020 Apr 22;17(4):e1003095. doi: 10.1371/journal.pmed.1003095 (PMC7176100; doi:10.1371/journal.pmed.1003095)
Supplement: S3 Text — (DOCX) [file pmed.1003095.s004.docx]

**S3 Text.**

**Carotid scan measurement manual**

**1: Static images will be stored as a JPEG file (BMP/TIFF also acceptable) and images will be measured at a central facility.**

Patient information will be removed for blinding.

**2: Recorded area should include: right and left images of the common carotid artery, bulb region, and internal carotid artery for a total of 6 image**s

(Add body marks to indicate left and right sides)

- Show ECGs and record the R wave time phase diastolic image.

- Longitudinal image: Common carotid artery images should be parallel to the horizontal line.

Try to obtain clear images of the common carotid artery far wall plaques^*1^ or double structure of the intima-media.

The subject should turn his face towards the converse side at a 45º angle and measurements taken at a 90º angle to the nose as a rule.

[1] Longitudinal image: (Changes in IMT over time) Take an image so that at least 1 cm of the IMT on the far wall, including the inflection point^*2^ from the common carotid artery will appear at the right end of the image (if a plaque is present in this area, take an image including the plaque and record without necessarily excluding the plaque).

[2] Bulb longitudinal image (to measure plaque): Take an image of the bulb plaque (if no plaque is present, take the far wall IMT image)

[3] Internal carotid artery longitudinal image (to measure plaque): Take an image so that the internal carotid artery cardiac side plaque is clearly imaged (if there is no plaque, take a far wall IMT image).

**3: Measurements**

Check the web registered image in electronic media to allow central assessment.

[1] Longitudinal image (measure IMT change over time) using automated software to determine maximum IMT of 10 mm length of the far wall cranial-side of the common carotid artery. Mean IMT is measured on both left and right sides and mean IMT is reported as calculated data of the primary endpoint.

[2] Bulb, internal carotid artery longitudinal image is similarly analyzed, and the parameters below are also reported as secondary endpoints.

・Max IMT: Maximum IMT at the common carotid artery, bulb, and internal carotid artery.

・Plaque gray scale median (GSM): Echogenicity median of the most hypoechoic plaque

・Plaque area: Plaque area of the most hypoechoic plaque

[3] Difference between the 12 month and 24-month values, percentage change of the above indicators

*1 Morphology is defined as irrelevant in hypertrophic plaque lesions ≥1.1 mm thick.

*2 If inflexion point is unclear, 10 mm towards the cardiac side from the bifurcation will be defined as the border between the carotid bulb and common carotid artery.

**Carotid Duplex Ultrasound Applications Manual**

1. **Preparing for measurement**

| **Examiner** | **Subject** |
| --- | --- |
| Scans may be taken either caudally or cranially. | Measurements: Supine position  The head should be tilted back so that the contralateral side is at a 45º angle.  Set a goniometer at the top of the head. |

**2. Diagnostic Ultrasound Device Preparation (Preset)**

| Save image  Digital photo image: JPEC file (BMP/TIFF also acceptable)  Anonymize patient information |
| --- |
| Probe  Linear Probe  Standard wavelength >7 MHz |
| Monitor  Depth: 4 cm x 3 cm  Focus: Single, near far wall  Show ECG |

**3. Measurement procedure**

| ***Ordering*** | ***Radial/Longitudinal image*** | ***Region*** | ***Operating procedures*** | ***Save image*** |
| --- | --- | --- | --- | --- |
|  |  |  |  |  |
| 1 | Radial image plaque screening | From common carotid artery to bifurcation | Check the entire common carotid artery for signs of plaque, and where lesions are. | None |
| 2 | Longitudinal CCA IMT image | 10 mm | The basic image should be a 90º lateral image, and a well visualized cross-section.  Extract so that vascular wall is parallel  Clear double structure on the far wall (Image 1)  Include plaque area if present  If common carotid artery and bulb region border is unclear, take an image of the entire region including the bifurcation and use the border on 10 mm from the bifurcation on the cardiac side to take the image. | Take at least 1 image each of the left and right sides (multiple images may be recorded) |
| 3 | Longitudinal bulb Plaque/IMT image | Bulb region | Extraction of long axial image of plaques found on radial image  Use IMT image if plaque is not present  Adjust gain so that the vascular lumen is echo-free, and the outer membrane is hyperechoic.  Do not use zoom function | At least 1 image on the right and left sides (multiple images may be recorded) |
| 4 | Longitudinal Plaque/IMT image | Internal carotid artery | Capture the longitudinal image of plaques seen on radial image  Use the IMT image if no plaque is present  Adjust gain so that the vascular lumen is echo-free, and the outer membrane is hyperechoic.  Do not use the zoom feature | Minimum of 1 image each on right and left sides (multiple images can be recorded) |

**Image 1. Carotid IMT Measurement Longitudinal image: Fresh image from the common carotid artery far wall 10 mm on the cranial side.**

<-- cardiac side

cranial side -->

CCA-bulb junction

Bifurcation

Near wall

Far wall

Bulb

ICA

Mean CCA-IMT

10 mm

CCA = common carotid artery, ICA = internal carotid artery.

* If border between common carotid artery and bulb is indistinct, use position 10 mm on the cardiac side from the bifurcation.

CCA


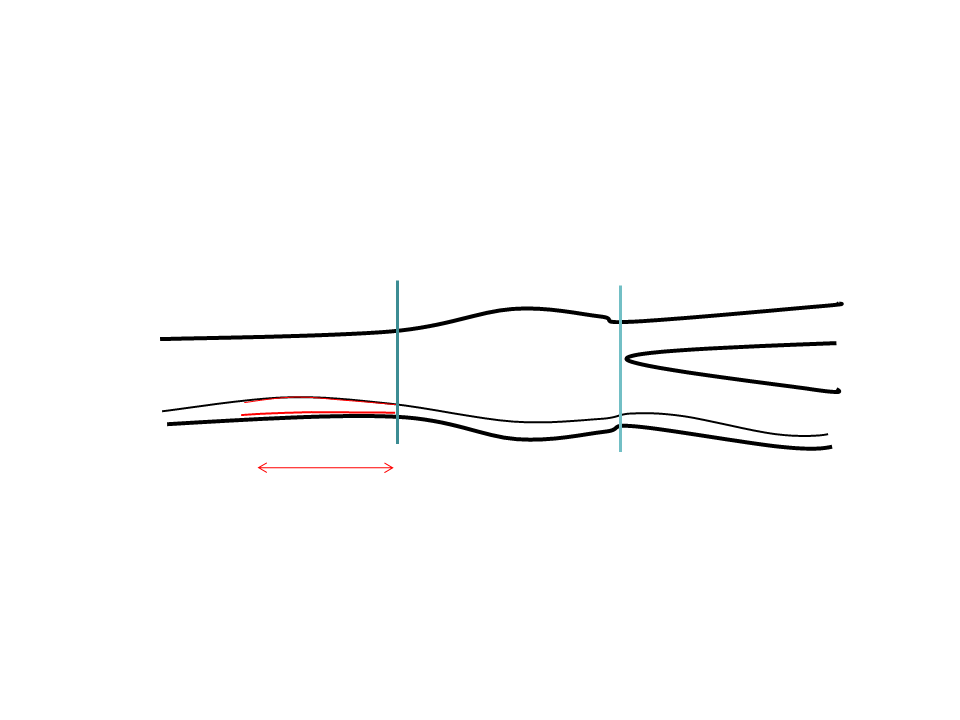


分岐部

総頸動脈・頸動脈洞境界

総頸動脈

頸動脈洞

内頸動脈


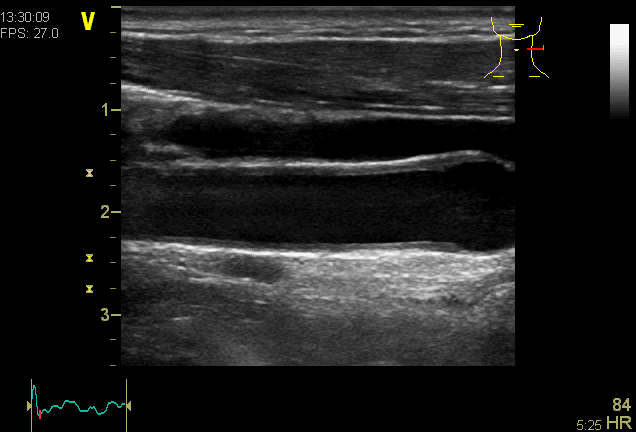


10ｍｍ

**4. Submit images for central facility evaluation**

Total of 6 images uploaded to web

Rt-CCA Lt-CCA


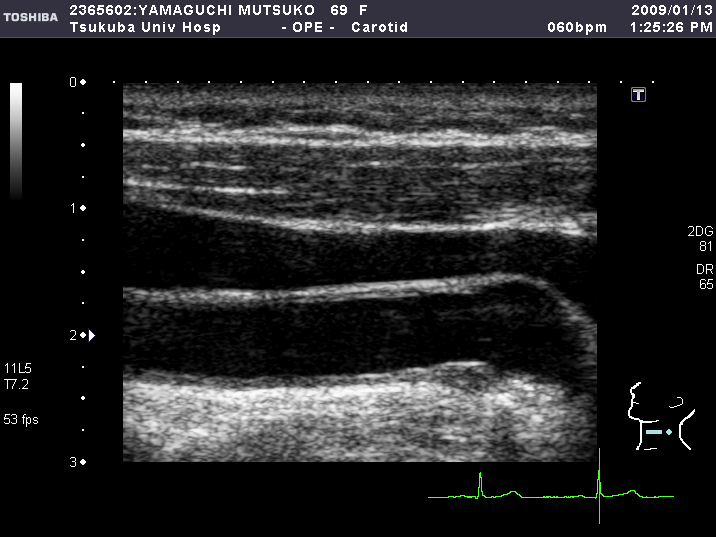

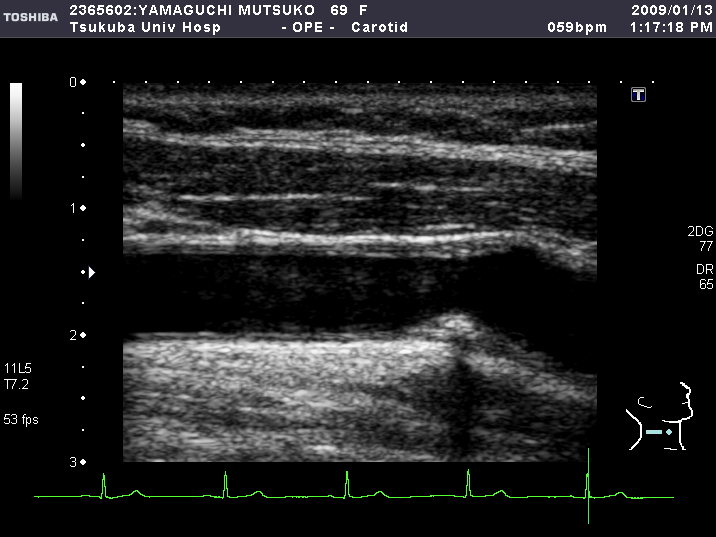


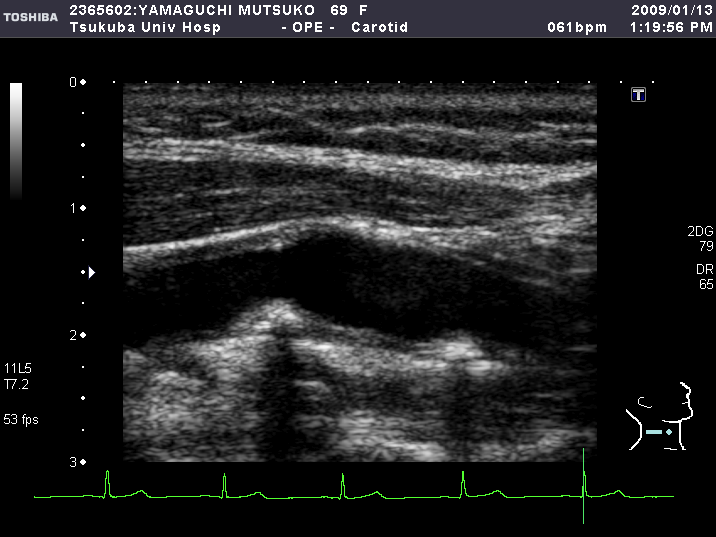

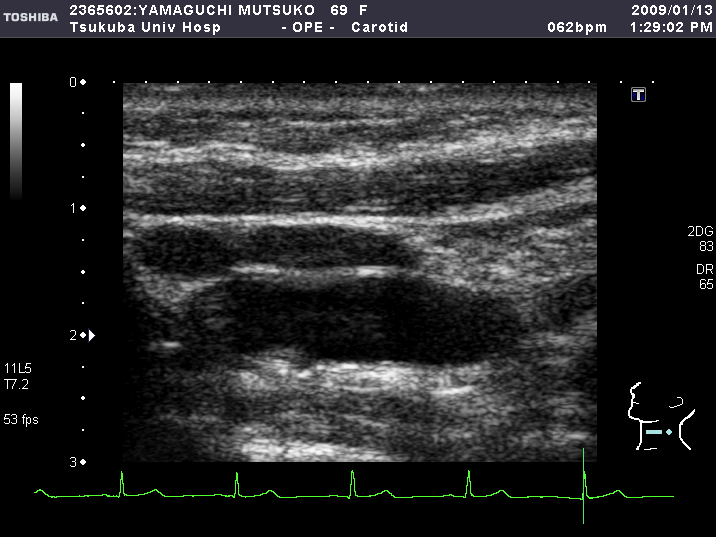

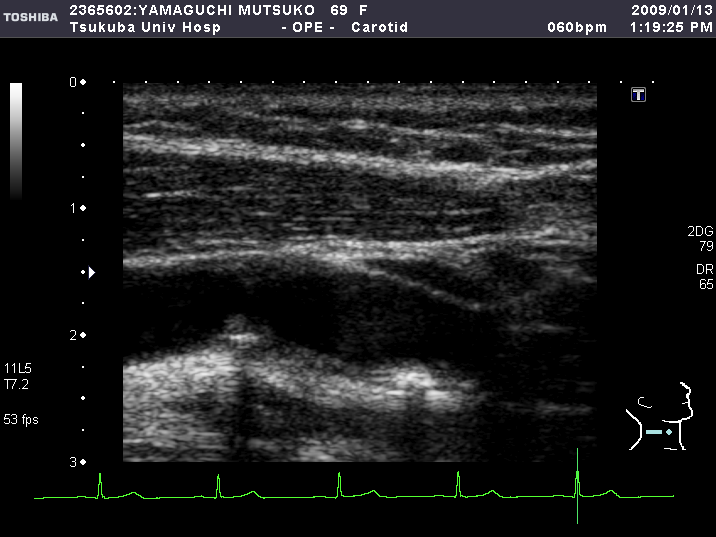
Rt-bulb Lt-bulb


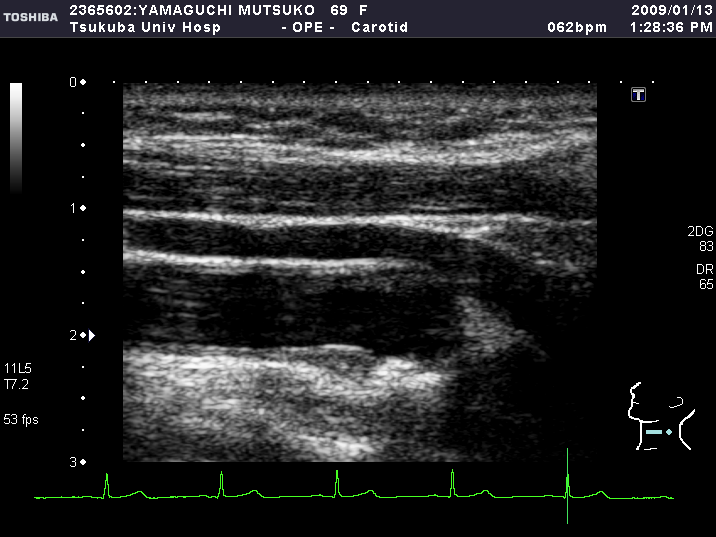


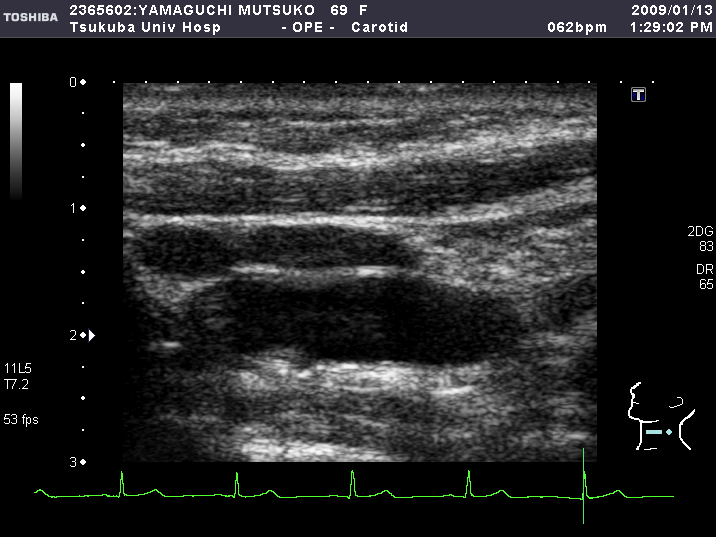

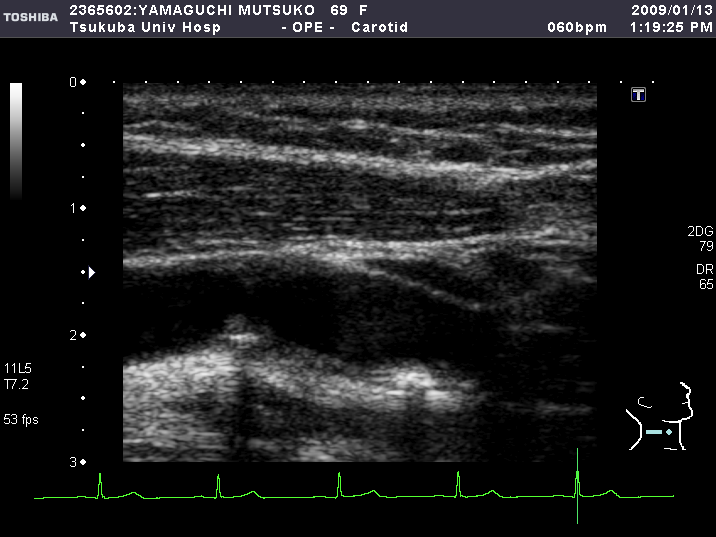
Rt-ICA Lt-ICA
