# Supplementary material for: Febuxostat does not delay progression of carotid atherosclerosis in patients with asymptomatic hyperuricemia: A randomized, controlled trial
Source: PLoS Med. 2020 Apr 22;17(4):e1003095. doi: 10.1371/journal.pmed.1003095 (PMC7176100; doi:10.1371/journal.pmed.1003095)
Supplement: S4 Text — (DOCX) [file pmed.1003095.s005.docx]

**S4 Text.**

**Statistical analysis plan**

A Collaborative Randomized Multicenter Comparative Study on Prophylaxis of Vascular Dysfunction with Febuxostat, a Xanthine Oxidase Inhibitor, in Hyperuricemia

Program of vascular evaluation under uric acid control
by xanthine oxidase inhibitor, febuxostat: multicenter, randomized controlled study

PRIZE study

**Principal investigator:**

Department of Respiratory Medicine, Saga Medical School Faculty of Medicine, Saga University

Koichi Noda

Statistical Analysis Plan Version 1.0 Date Approved: October 01, 2015

Statistical Analysis Plan Version 1.1 Date Approved: January 11, 2019

**Lead Statistician:**

Associate Professor, Department of Public Health, Keio University Hospital

Keio University Hospital Clinical and Translational Research Center Biostatistics
 Department Head

Yasunori Sato

Statistical Analysis Plan Ver 1.0 Approved on: October 01, 2015.

Statistical Analysis Plan Ver 1.1 Approved on: January 11, 2019.

**Revision History:**

| **Date written** | **Revision No.** |
| --- | --- |
| July 17, 2015 | 1.0 |
| January 10, 2019 | 1.1 |

# **Definition of terms**

Patient characteristics: sex, drinking and smoking history, pre- or post-menopause (women)

complications, past medical history: diabetes mellitus, kidney disease (eGFR< 60 mL/min/1.73 m^2^, other), liver disease, arteriosclerotic cerebro-cardiovascular disease (myocardial infarction, angina pectoris, stroke, ASO), heart failure, PCI, CABF, arrhythmias, hypertension, lipid abnormalities, gouty arthritis, etc.

Current pharmacotherapy: antihypertensive drugs (calcium antagonists, ACE inhibitors, ARB, beta blockers, diuretics, renin inhibitors, etc.), antidiabetic agent (sulfonylureas), biguanides, alpha-glycosidase inhibitor, thiazolidines, DPP-4 inhibitors, GLP-1 analogues, insulin preparations, etc.), dyslipidemia treatment drugs (statins, fibrate preparations, EPA preparation, small intestine absorption inhibitor, resin, other), antiplatelet drugs (aspirin, ticlopidine, clopidogrel, silostazol, etc.)

Physiological exams: height, weight, BMI, blood pressure, pulse

Carotid duplex exam: Common carotid artery, bulb, internal carotid artery

Cardiovascular function test: echocardiography (systolic/diastolic function, left atrial diameter, left ventricular weight coefficient), flow mediated dilation (FMD), pulse wave velocity (PWV), cardio-ankle vascular index (CAVI), arteriosclerosis index (AI)

Hematology: red blood cell count, white blood cell count, hemoglobin, hematocrit, platelet count

Blood biochemistry: AST, ALT, LDH, BUN, Na, K, Cl, blood glucose, serum uric acid, serum lipids (TC, HDL-C, TG, non-HDL-C, LDL-C [indirect method]), serum creatinine, eGFR

Special blood biochemistry tests: NT-proBNP, high sensitivity CRP, 1,5AG, small granule LDL, RLP-C, MDA-LDL, serum cystatin C, RAGE, high molecular adiponectin, high sensitivity troponin I, ANGPTL2

Urinalysis: urinary albumin excretion (creatinine corrected), urinary L-FABP (if complicated by diabetes or CKD)

Summary statistics: no. of subjects, mean, standard deviation, mean 95% confidence interval, minimum value, 1st quartile, median, 3rd quartile, maximum value

Amount of change: (current measured value) - (baseline value)

Percentage change: [(measured value at each time point) - (baseline value)]/(baseline value)

Incidence: incidence of adverse events and adverse drug reactions are defined as follows.

(percentage change) = (no. of cases reported)/(no. of cases in analysis set) x 100

# **Study objectives**

The objective of this study is to compare the investigational treatment (febuxostat group) to a group where the natural clinical course is observed (control group) on the carotid artery intimal media thickness (IMT) as measured using carotid duplex imaging as an indicator of febuxostat efficacy in delaying atherosclerotic progression. See the Study Protocol Version 5.4 for the study design. Details regarding statistical analysis in the final analyses are described after chapter 3.

# **Analysis sets**

## Definition of analysis set for final analysis

### Full Analysis Set (FAS)

The analysis set comprises a maximum analysis set that includes all patients enrolled in this study with the exception of the following groups:

- Subjects who withdrew consent

- Patients with no primary endpoint (mean IMT for the common carotid artery) data after randomization.

### PPS (Per Protocol Set)

Those with the following serious study protocol violations regarding study method or concomitant therapy will be excluded from the FAS.

- Violation of inclusion criteria

- Violation of exclusion criteria

- Violation of concomitant drug use

- Violation of concomitant treatment use

### Safety analysis set:

Randomly allocated patients with data after allocation.

## Handling statistical analysis items

The FAS will be the main analysis set for this study and it will be used in the primary efficacy endpoint and secondary endpoint analyses. Primary and secondary endpoints for efficacy will be analyzed in the PPS to determine sensitivity of the FAS analysis results.

Cardiovascular-renal events and all safety analyses will be continued for the safety analysis set.

## **Data set for analysis and data handling**

- Data where follow-up is complete and fixed will be analyzed. Moreover, data in cases of duplicated enrollment will be analyzed after deleting all unnecessary data.
- The amount of change and percentage change definitions are as stated below.

Amount of change (current measured value) - (baseline value)

Percentage change = (measured value after intervention - baseline
 measurement)/baseline measurement

- Transformation of variables
   If the measured values do not follow a normal distribution, logarithmic
   transformation or other transformations will be performed.
- Handling missing data

Missing data need not be considered for primary and secondary endpoints, but for the sensitivity analysis, mixed effects model for repeated measures (MMRM) and LOCF, multiple substitutions will be used as necessary and stability of analysis results assessed.

- Since IMT will be measured in both the left and right blood vessels, the percentage
   change of IMT can be determined on both the left and right sides and the mean will
   be used as a primary endpoint. However, if findings are only discovered unilaterally
   on either the left or right, the side where findings are present will be used.

# **Analysis of primary endpoint**

Primary efficacy endpoint is the percentage change in mean IMT after 24 months of treatment. Differences in percentage change of mean IMT of the common carotid artery are assessed for statistical significance. An analysis of covariance will be performed where the fixed-effect and randomized adjustment factors will be considered covariant and tested on a null hypothesis that the percentage change in mean common carotid artery IMT of the two groups is equal. Randomized adjustment factors include: Age (<65 years or ≥65 years), sex (male/female), diabetes complications (present/absent), serum uric acid level (<8.0 mg/dL or ≥8.0 mg/dL, and maximum IMT during institutional measurement (<1.3 mm or ≥1.3 mm). Moreover, the summary statistics (no. of subjects, mean, standard deviation, minimum, median, and maximum values) for the percentage change at 24 months will be calculated. Also, the 95% confidence interval (two-sided) with a 5% level of significance (two-sided) will be calculated.

For the sensitivity analysis, changes in mean common carotid artery IMT over time will be shown for each group. Data measured over time will be analyzed in a mixed effect model for repeated measures (MMRM) using an unstructured correlation structure. Factors will include the treatment effect, timing effect, and interaction between treatment and timing as fixed effects, while interaction and amount of change effects will be the subjects nested in the group effect. However, if the correlation structure is unstructured and the calculation results do not converge, the composite symmetry (CS) will be used.

# **Analysis of secondary endpoints**

## Carotid duplex (mean IMT, maximum IMT, plaque area, plaque echogenicity of the common carotid artery, bulb, and internal carotid artery)

For each endpoint, summary statistics for measured value, amount of change, and percentage change (no. of subjects, mean, standard deviation, minimum value, median, and maximum value) after 0, 12, and 24 months of treatment will be calculated. Student's *t*-test will be used to test the null hypothesis that the measured values, change, and percentage change between the two groups will be equal. The 95% confidence interval based on a *t*-distribution of the difference in measured value, change and percentage change of each endpoint will be calculated. The level of significance will be 5% (two-sided). Adjustment will not be made for multiplicity.

## 6.2 Serum uric acid

Summary statistics (no. of subjects, means, standard deviation, minimum values, median, maximum value) of measured values, amount of change, and percentage change after 0, 6, 12, and 24 months of treatment will be calculated. Using Student's *t*-test, based on a null hypothesis that measurements, and differences in the amount of change and percentage change between the 2 groups will be equal, the 95% confidence interval based on a t-distribution will be calculated.

## 6.3 Analysis of clinical laboratory test values and biomarkers

For each endpoint, the summary statistics (no. of subjects, mean, standard deviation, Minimum value, median, and maximum) for the measured value, amount of change, and percentage change for each time point will be calculated. Student's *t*-test will be used to test the null hypothesis that the measured values, change, and percentage change between the two groups will be equal. The 95% confidence interval based on a *t*-distribution of the difference in measured value, change and percentage change of each endpoint will be calculated.

# **Analysis of exploratory endpoints**

## 7.1 Cardiovascular function tests

For each endpoint, the summary statistics (no. of subjects, mean, standard deviation, minimum value, median, and maximum) for the measured value, amount of change, and percentage change at 0, 12, and 24 months of treatment will be calculated. Student's *t*-test will be used to test the null hypothesis that the measured values, change, and percentage change between the two groups will be equal. The 95% confidence interval based on a *t*-distribution of the difference in measured value, change and percentage change of each endpoint will be calculated.

## 7.2 Special blood biochemistry tests

For each endpoint, the summary statistics (no. of subjects, mean, standard deviation, minimum value, median, and maximum) for the measured value, amount of change, and percentage change for each time point will be calculated. Student's *t*-test will be used to test the null hypothesis that the measured values, change, and percentage change between the two groups will be equal. The 95% confidence interval based on a *t*-distribution of the difference in measured value, change and percentage change of each endpoint will be calculated.

# **Analysis of the safety evaluation items**

## Analysis of events (cardiovascular death, non-fatal myocardial infarction and stroke, renal events, total deaths).

Cardiovascular death, non-fatal myocardial infarction and stroke, renal events (serum creatinine doubles, introduction of hemodialysis, etc.), total deaths, or a combination of such events will be summarized into a list for each group. A K-M plot will be created for each event as necessary and groups compared using log-rank test while Cox regression will be performed to estimate the hazard ratio.

## Adverse events

The number of adverse events and percentage of patients with adverse events will be determined. A table of adverse events will be created for each group. Fisher's exact test will be used to compare groups as needed.

# **Statistical analysis plan based on subject characteristics**

Distribution of patient characteristics data and summary statistics are calculated in each group. Category incidence and percentage will be shown for all nominal variables. For continuous variables, the summary statistics (no. of cases, mean, standard deviation, minimum value, median, maximum value) will be calculated for each group. Comparison between groups will be performed using Fisher's exact probability test for the nominal variables and Student's *t*-test for the continuous variables. The level of significance will be 5% (two-sided). A list of nominal variables and continuous variables are shown below

## Nominal variables

Sex, drinking/smoking history, pre- or post-menopause (women), complications/past medical history: diabetes, kidney disease (eGFR<60 mL/min/1.73m^2^, other), liver disease, arteriosclerotic cerebrovascular disease (myocardial infarction, angina, stroke, ASO), heart failure, PCI, CABG, arrhythmia, hypertension, lipid abnormalities, gouty arthritis, etc.

Ongoing pharmacotherapy: antihypertensive drugs (calcium antagonists, ACE inhibitors, ARB, beta blockers, diuretics, renin inhibitors, etc.), antidiabetic agent (sulfonylureas), biguanides, alpha-glycosidase inhibitor, thiazolidines, DPP-4 inhibitors, GLP-1 analogues, insulin preparations, etc.), dyslipidemia treatment drugs (statins, fibrate preparations, EPA preparation, small intestine absorption inhibitor, resin, other), antiplatelet drugs (aspirin, ticlopidine, clopidogrel, silostazol, etc.)

## Continuous variables

Age, height, weight, BMI, pulse, outpatient blood pressure (systolic, diastolic) maximum IMT, and clinical laboratory test values such as eGFR, LDL, HDL, TG, TC, non-HDL, serum uric acid, fasting blood glucose.

# **Subgroup (stratified analysis)**

Patients will be stratified into the following groups based on patient characteristics and analyses similar to 5 will be performed.

- BMI: <25% vs ≥25%
- Hypertension: Present vs Absent (sBP≥ 140 mmHg or use of antihypertensive drug is defined as having hypertension)
- eGFR: <60 vs ≥60
- Arteriosclerotic cerebro-cardiovascular disease: Present vs Absent (Disease definition: cerebral infarction, TIA, myocardial infarction, angina pectoris, PCI, CABG, other such as ASO and lower limb and cervical artery revascularization)
- Gouty arthritis: Present or Absent (1 year or longer after eligibility confirmation).
- Statins: Used vs Not used
- Antiplatelet agents: Used vs not used

# **Additional analysis**

If necessary, additional analyses will be conducted. If an additional analysis is performed, the decision date and the reason for adding an analysis must be documented.

# **Overall concerns in data analysis**

## 12.1 Statistical analysis software

The figures, tables, and lists planned in this statistical analysis plan will be created using Windows 8.1 or later and SAS version 9.4 or later. Results will be saved as Microsoft Excel sheets and PDFs. If using originally coded statistical software other than SAS, this should be documented in the Statistical Analysis Report. SAS programs created for individual analysis processing will not be validated.

## 12.2 Overall considerations regarding figures, tables, and list format

A4-sized paper will be used. Generally, figures and summary tables are in portrait view while lists and data lists are created in landscape view, but this can be decided based on the individual figure or table.

## 12.3 Common rules in data processing

- Rules regarding number of days and duration

1. When calculating the number of days, subtract the start date from the end date and
    add 1.

(Example)

- Days of administration: If administration is discontinued on the day it is started, the
   dosing
   duration is considered 1 day.
- Time until appearance of adverse events: If an adverse event occurs the day after the start
   of administration, the number of days to the onset of an adverse event will be 2 days.
- Survival time: If the patient dies the day after the start of administration, the survival time
   will be 2 days.

1. When calculating days to years, months, and weeks, 1 year is equivalent to 365.25
    days, 1 month is 30.4375 days, and 1 week is 7 days.
2. When expressing as "duration (days) from the start of administration" or "time of
    onset
    (day)" the day administration started is considered day 1, and the day before
    administration is -1. Thereafter the days should be counted up or counted down for
    each day.

- Rules on rounding after decimal point and how to round numbers.

1. Mean, standard deviation, and median will be rounded to 1 decimal place (round off
    the 2nd decimal place)
2. Percentages will be expressed up to 1 decimal place below the point (e.g. 12.3%).
    The 2nd decimal point will be rounded off.
3. If statistical amounts for mean, standard deviation, median are calculated, rounding
    will not be done during calculation, but instead, the final calculation results will be
    rounded.

- Survival curve

Estimation of the survival curve using Kaplan-Meier will be plotted according to the SAS LIFETEST procedure. Together with the survival curve graph, the number of cases, number of deaths (or no. of events), median survival time (MST) and their 95% confidence intervals will be shown. If the confidence interval is indefinite, a "-" (hyphen) will be used. If days of survival are converted to years and months, the rules of the above days and duration must be followed.

The 95% confidence interval for median survival time will be calculated based on a sign test for censored data.

# **Modification History**

## Version history of the statistical analysis plan

| Date | Modifications, etc. |
| --- | --- |
| January 10, 2019 | Documentation of patients subject to the cardiovascular event assessment, addition of data collection items, and subgroup definitions |
