# Supplementary material for: Febuxostat does not delay progression of carotid atherosclerosis in patients with asymptomatic hyperuricemia: A randomized, controlled trial
Source: PLoS Med. 2020 Apr 22;17(4):e1003095. doi: 10.1371/journal.pmed.1003095 (PMC7176100; doi:10.1371/journal.pmed.1003095)
Supplement: S1 Table — (DOCX) [file pmed.1003095.s007.docx]

**S1 Table. Inclusion and exclusion criteria for the PRIZE study**

| **Inclusion** | **Exclusion** |
| --- | --- |
| Adults (aged ≥20 years) | Patients being treated with any of the following SUA-lowering agents within 8 weeks before confirmation of the eligibility criteria: allopurinol, benzbromarone, probenecid, bucolome, topiroxostat, or febuxostat |
| Patients with asymptomatic hyperuricemia with a SUA >7.0 mg/dL | Patients being treated with any of the following agents at the time of confirmation of the eligibility criteria: mercaptopurine hydrate, azathioprine, vidarabine, or didanosine |
| Patients with a maximum IMT of the common carotid artery ≥1.1 mm | Patients who have undergone an operation or who have severe infections or serious injury at the time of confirmation of the eligibility criteria |
| The patient provided written informed consent to participate in the study | Patients who had a myocardial infarction, angina pectoris, percutaneous transluminal coronary angioplasty/bypass surgery, cerebral infarction, cerebral hemorrhage, subarachnoid hemorrhage, or transient cerebral ischemic attack within 3 months before confirmation of the eligibility criteria |
|  | Patients with advanced heart failure (NYHA functional classification IV) |
|  | Patients with gouty tophus, or those who have subjective symptoms of gout arthritis within 1 year before confirmation of the eligibility criteria |
|  | Patients with a complication or a disease history (eGFR <30 mL/min/1.73 m^2^ or patients on dialysis) |
|  | Patients with severe liver dysfunction (AST or ALT ≥2 times the upper limit of the institutional standard value) |
|  | Patients with a complication or a disease history (e.g. malignancy) who are considered not eligible for the study by the investigators |
|  | Patients with a history of hypersensitivity to febuxostat |
|  | Pregnant, possibly pregnant, or lactating women or those who wish to become pregnant during participation in the study |
|  | Patients who have undergone CEA or CAS surgery |
|  | Patients who are considered not eligible for the study by the investigators due to other reasons |

Abbreviations: ALT, alanine aminotransferase; AST, aspartate transaminase; CAS, carotid artery stenting; CEA, carotid endarterectomy; eGFR, estimated glomerular filtration rate; IMT, intima-media thickness; NYHA, New York Heart Association; SUA, serum uric acid.
