# Supplementary material for: Febuxostat does not delay progression of carotid atherosclerosis in patients with asymptomatic hyperuricemia: A randomized, controlled trial
Source: PLoS Med. 2020 Apr 22;17(4):e1003095. doi: 10.1371/journal.pmed.1003095 (PMC7176100; doi:10.1371/journal.pmed.1003095)
Supplement: S2 Table — (DOCX) [file pmed.1003095.s008.docx]

**S2 Table. Sensitive analysis for primary endpoint**

| **Method** | **Adjusted percentage change at 24 months (95% CI)** | | **Group difference (95% CI)** | ***P* Value** |
| --- | --- | --- | --- | --- |
|  | **Febuxostat (*n* = 239)** | **Control (*n* = 244)** |  |  |
| MMRM | 0.0053 (0.0052 to 0.0054) | 0.0052 (0.0051 to 0.0054) | 0.0001 (-0.0001 to 0.0002) | 0.43 |

Abbreviations: CI, confidential interval; MMRM, mixed-effects model for repeated measures.
