# Supplementary material for: Febuxostat does not delay progression of carotid atherosclerosis in patients with asymptomatic hyperuricemia: A randomized, controlled trial
Source: PLoS Med. 2020 Apr 22;17(4):e1003095. doi: 10.1371/journal.pmed.1003095 (PMC7176100; doi:10.1371/journal.pmed.1003095)
Supplement: S3 Table — (DOCX) [file pmed.1003095.s009.docx]

**S3 Table. Changes in clinical and laboratory parameters**

| **Variable** | **Time point** | **Febuxostat** | | **Control** | | **Group difference (95% CI)** | ***P* Value** |
| --- | --- | --- | --- | --- | --- | --- | --- |
|  |  | **Number** | **Mean (SD)** | **Number** | **Mean (SD)** |  |  |
| Uric acid, mg/dL | Baseline | 238 | 7.76 (0.98) | 241 | 7.73 (1.04) | 0.03 (-0.15 to 0.21) | 0.75 |
|  | 6 months | 229 | 5.14 (1.26) | 230 | 7.43 (1.14) | -2.28 (-2.50 to -2.06) | <0.001 |
|  | 12 months | 216 | 5.06 (1.32) | 219 | 7.31 (0.99) | -2.62 (-2.47 to -2.03) | <0.001 |
|  | 24 months | 213 | 4.66 (1.27) | 204 | 7.28 (1.27) | -2.62 (-2.86 to -2.38) | <0.001 |
| Delta from baseline, mg/dL | 6 months | 228 | -2.63 (1.35) | 227 | -0.31 (1.17) | -2.31 (-2.55 to -2.08) | <0.001 |
|  | 12 months | 215 | -2.69 (1.42) | 216 | -0.38 (1.21) | -2.31 (-2.56 to -2.06) | <0.001 |
|  | 24 months | 212 | -3.07 (1.30) | 202 | -0.42 (1.55) | -2.65 (-2.93 to -2.37) | <0.001 |
| Systolic blood pressure, mm Hg | Baseline | 236 | 128.94 (14.67) | 236 | 127.03 (15.69) | 1.91 (-0.84 to 4.66) | 0.17 |
|  | 12 months | 204 | 128.32 (15.19) | 206 | 130.13 (16.28) | -1.80 (-4.86 to 1.25) | 0.25 |
|  | 24 months | 198 | 128.47 (14.26) | 181 | 128.44 (15.97) | 0.03 (-3.04 to 3.10) | 0.99 |
| Delta from baseline, mm Hg | 12 months | 201 | -0.79 (15.53) | 202 | 3.77 (16.47) | -4.56 (-7.70 to -1.43) | 0.004 |
|  | 24 months | 197 | -0.52 (15.15) | 179 | 1.46 (17.97) | -1.98 (-5.37 to 1.41) | 0.25 |
| Diastolic blood pressure, mm Hg | Baseline | 235 | 73.30 (11.64) | 236 | 74.18 (11.42) | -0.88 (-2.97 to 1.20) | 0.41 |
|  | 12 months | 203 | 72.86 (12.16) | 206 | 74.31 (11.59) | -1.45 (-3.76 to 0.86) | 0.22 |
|  | 24 months | 198 | 72.48 (11.08) | 181 | 73.76 (10.43) | -1.28 (-3.45 to 0.90) | 0.25 |
| Delta from baseline, mm Hg | 12 months | 199 | -0.42 (12.44) | 202 | 0.48 (11.98) | -0.90 (-3.30 to 1.50) | 0.46 |
|  | 24 months | 196 | -0.79 (11.70) | 179 | -0.07 (11.30) | -0.72 (-3.05 to 1.62) | 0.55 |
| BMI, kg/m^2^ | Baseline | 239 | 24.84 (3.95) | 239 | 24.97 (3.52) | -0.13 (-0.80 to 0.54) | 0.70 |
|  | 12 months | 189 | 24.87 (3.86) | 189 | 25.14 (3.52) | -0.26 (-1.01 to 0.48) | 0.49 |
|  | 24 months | 200 | 24.85 (4.16) | 192 | 25.05 (3.60) | -0.20 (-0.97 to 0.57) | 0.61 |
| Delta from baseline, kg/m^2^ | 12 months | 189 | 0.09 (1.06) | 189 | -0.06 (1.21) | 0.15 (-0.08 to 0.38) | 0.19 |
|  | 24 months | 200 | -0.06 (2.25) | 191 | -0.21 (1.45) | 0.15 (-0.23 to 0.52) | 0.44 |
| Fasting plasma glucose, mg/dL | Baseline | 154 | 117.39 (27.86) | 157 | 117.05 (38.91) | 0.34 (-7.20 to 7.88) | 0.93 |
|  | 12 months | 131 | 118.34 (29.01) | 134 | 118.81 (32.94) | -0.47 (-7.97 to 7.03) | 0.90 |
|  | 24 months | 139 | 117.6 (28.25) | 122 | 115.62 (23.21) | 1.97 (-4.30 to 8.25) | 0.54 |
| Delta from baseline, mg/dL | 12 months | 113 | 1.70 (20.80) | 113 | -1.19 (32.24) | 2.89 (-4.08 to 9.85) | 0.42 |
|  | 24 months | 111 | 0.26 (24.96) | 100 | -2.46 (39.65) | 2.72 (-6.40 to 11.84) | 0.56 |
| HDL-C, mg/dL | Baseline | 235 | 52.41 (14.05) | 237 | 52.94 (13.68) | -0.53 (-3.04 to 1.98) | 0.68 |
|  | 12 months | 214 | 53.11 (16.25) | 215 | 53.37 (14.29) | -0.27 (-3.17 to 2.64) | 0.86 |
|  | 24 months | 212 | 53.34 (15.33) | 202 | 52.78 (14.76) | 0.56 (-2.35 to 3.47) | 0.71 |
| Delta from baseline, mg/dL | 12 months | 211 | 0.76 (11.71) | 208 | 0.20 (8.56) | 0.55 (-1.41 to 2.52) | 0.58 |
|  | 24 months | 208 | 1.27 (9.84) | 197 | 0.33 (9.15) | 0.94 (-0.91 to 2.80) | 0.32 |
| Non-HDL-C, mg/dL | Baseline | 229 | 130.40 (32.51) | 227 | 128.63 (34.14) | 1.78 (-4.36 to 7.91) | 0.57 |
|  | 12 months | 208 | 125.09 (32.89) | 209 | 126.82 (38.59) | -1.73 (-8.63 to 5.17) | 0.62 |
|  | 24 months | 207 | 122.80 (31.89) | 198 | 122.69 (34.67) | 0.10 (-6.41 to 6.62) | 0.98 |
| Delta from baseline, mg/dL | 12 months | 203 | -4.97 (22.20) | 199 | -1.71 (28.04) | -3.26 (-8.23 to 1.70) | 0.20 |
|  | 24 months | 201 | -7.72 (24.70) | 187 | -4.81 (28.17) | -2.91 (-8.21 to 2.40) | 0.28 |
| Triglyceride, mg/dL | Baseline | 235 | 154.87 (95.22) | 238 | 153.60 (102.24) | 1.27 (-16.58 to 19.12) | 0.89 |
|  | 12 months | 215 | 144.84 (83.11) | 215 | 150.88 (102.41) | -6.04 (-23.72 to 11.65) | 0.50 |
|  | 24 months | 212 | 144.32 (89.65) | 201 | 147.90 (87.46) | -3.58 (-20.72 to 13.55) | 0.68 |
| Delta from baseline, mg/dL | 12 months | 212 | -11.62 (90.53) | 209 | -1.60 (83.94) | -10.01 (-26.74 to 6.71) | 0.24 |
|  | 24 months | 208 | -9.16 (87.62) | 197 | -4.37 (95.39 | -4.80 (-22.72 to 13.12) | 0.60 |
| eGFR, mL/min/1.73m^2^ | Baseline | 238 | 56.26 (15.41) | 239 | 57.12 (15.83) | -0.86 (-3.68 to 1.95) | 0.55 |
|  | 12 months | 215 | 55.47 (17.16) | 218 | 56.35 (15.87) | -0.88 (-4.00 to 2.24) | 0.58 |
|  | 24 months | 213 | 54.05 (16.52) | 203 | 55.11 (17.22) | -1.06 (-4.32 to 2.19) | 0.52 |
| Delta from baseline, mL/min/1.73m^2^ | 12 months | 214 | -0.49 (7.98) | 215 | -0.48 (7.18) | -0.02 (-1.46 to 1.43) | 0.98 |
|  | 24 months | 212 | -1.91 (8.12) | 199 | -2.06 (8.22) | 0.15 (-1.44 to 1.74) | 0.85 |

Abbreviations: BMI, body mass index; CI, confidential interval; eGFR, estimated glomerular filtration rate; HDL-C, high-density lipoprotein cholesterol; SD, standard deviation.

SI conversion factor: To convert uric acid to µmol/L, multiply by 59.48; to convert fasting plasma glucose to mmol/L, multiply by 0.0555; to convert HDL-C and non-HDL-C to mmol/L, multiply by 0.0259; to convert triglyceride to mmol/L, multiply by 0.0113.
