# Supplementary material for: Febuxostat does not delay progression of carotid atherosclerosis in patients with asymptomatic hyperuricemia: A randomized, controlled trial
Source: PLoS Med. 2020 Apr 22;17(4):e1003095. doi: 10.1371/journal.pmed.1003095 (PMC7176100; doi:10.1371/journal.pmed.1003095)
Supplement: S4 Table — (DOCX) [file pmed.1003095.s010.docx]

**S4 Table. Summary of adverse events**

| **Adverse event** | **Febuxostat (*n* = 241)** | **Control (*n* = 247)** |
| --- | --- | --- |
| Any adverse events, number (%) | 52 (21.6)  [88 events] | 48 (19.4)  [83 events] |
| Severity of adverse events^a^ |  |  |
| Mild, number (%) | 25 (10.4)  [31 events] | 12 (4.9)  [16 events] |
| Moderate, number (%) | 29 (12.0)  [43 events] | 27 (10.9)  [46 events] |
| Severe, number (%) | 13 (5.4)  [14 events] | 16 (6.5)  [21 events] |

^a^ Judged by local investigators.
