# Supplementary material for: Febuxostat does not delay progression of carotid atherosclerosis in patients with asymptomatic hyperuricemia: A randomized, controlled trial
Source: PLoS Med. 2020 Apr 22;17(4):e1003095. doi: 10.1371/journal.pmed.1003095 (PMC7176100; doi:10.1371/journal.pmed.1003095)
Supplement: S5 Table — (DOCX) [file pmed.1003095.s011.docx]

**S5 Table. Individual adverse events reported**

| **Adverse event** | **Febuxostat (*n* = 241)** | **Control (*n* = 247)** |
| --- | --- | --- |
| Abnormal laboratory data | 3 (1.2) | 1 (0.4) |
| Acute kidney injury | 0 (0.0) | 1 (0.4) |
| Acute myelocytic leukemia | 1 (0.4) | 0 (0.0) |
| acute cholecystitis | 1 (0.4) | 0 (0.0) |
| Albuminuria | 2 (0.8) | 0 (0.0) |
| Anemia | 2 (0.8) | 2 (0.8) |
| Angina pectoris | 3 (1.2) | 1 (0.4) |
| Anuresis | 0 (0.0) | 1 (0.4) |
| Anxiety disorder | 0 (0.0) | 1 (0.4) |
| Aortic aneurysm | 2 (0.8) | 1 (0.4) |
| Apendicitis | 0 (0.0) | 2 (0.8) |
| Arteriosclerosis obliterans | 0 (0.0) | 1 (0.4) |
| Asthma | 0 (0.0) | 1 (0.4) |
| Atrial fibrillation | 2 (0.8) | 0 (0.0) |
| Bipolar disorder | 0 (0.0) | 1 (0.4) |
| Body weight loss | 1 (0.4) | 0 (0.0) |
| Bone fracture | 2 (0.8) | 2 (0.8) |
| Brain cancer | 0 (0.0) | 1 (0.4) |
| Cardiac sudden death | 0 (0.0) | 2 (0.8) |
| Cataract | 1 (0.4) | 1 (0.4) |
| Chest pain | 0 (0.0) | 1 (0.4) |
| Chronic kidney disease | 1 (0.4) | 0 (0.0) |
| Colon Cancer | 0 (0.0) | 1 (0.4) |
| Colon perforation | 0 (0.0) | 1 (0.4) |
| Colon polyp | 1 (0.4) | 1 (0.4) |
| Constipation | 1 (0.4) | 0 (0.0) |
| Dehydration | 0 (0.0) | 1 (0.4) |
| Diabetes | 2 (0.8) | 0 (0.0) |
| Diabetic coma | 0 (0.0) | 1 (0.4) |
| Diabetic ketoacidosis | 0 (0.0) | 1 (0.4) |
| Diabetic retinopathy | 0 (0.0) | 1 (0.4) |
| Diabetic renal disease | 1 (0.4) | 0 (0.0) |
| Diarrhea | 1 (0.4) | 0 (0.0) |
| Endocarditis | 0 (0.0) | 1 (0.4) |
| Facial nerve paralysis | 1 (0.4) | 0 (0.0) |
| Fatal myocardial infarction | 1 (0.4) | 0 (0.0) |
| Fatigue | 1 (0.4) | 0 (0.0) |
| Fever | 0 (0.0) | 1 (0.4) |
| Gait disorder | 1 (0.4) | 0 (0.0) |
| Gastric cancer | 2 (0.8) | 1 (0.4) |
| Glaucoma | 1 (0.4) | 0 (0.0) |
| Gout arthritis | 0 (0.0) | 4 (1.6) |
| Headache | 1 (0.4) | 0 (0.0) |
| Heart failure | 5 (2.1) | 6 (2.4) |
| Heart failure death | 0 (0.0) | 1 (0.4) |
| Hyperglycemia | 2 (0.8) | 0 (0.0) |
| Hyperkalemia | 1 (0.4) | 0 (0.0) |
| Hyperkeratosis | 0 (0.0) | 1 (0.4) |
| Hypoglycemia | 0 (0.0) | 1 (0.4) |
| Hyponatremia | 0 (0.0) | 1 (0.4) |
| Impaired liver function | 9 (3.7) | 3 (1.2) |
| Impaired renal function | 1 (0.4) | 2 (0.8) |
| Influenza | 0 (0.0) | 1 (0.4) |
| Lid edema | 1 (0.4) | 0 (0.0) |
| Loss of appetite | 1 (0.4) | 0 (0.0) |
| Melena | 1 (0.4) | 1 (0.4) |
| Myeloma | 0 (0.0) | 1 (0.4) |
| Nausea | 1 (0.4) | 0 (0.0) |
| Non-fatal myocardial infarction | 0 (0.0) | 1 (0.4) |
| Non-fatal stroke | 3 (1.2) | 5 (2.0) |
| Orchiditis | 1 (0.4) | 0 (0.0) |
| Pain in back | 1 (0.4) | 0 (0.0) |
| Pancreas cancer | 0 (0.0) | 1 (0.4) |
| Pancreatitis | 1 (0.4) | 0 (0.0) |
| Paresthesia | 1 (0.4) | 0 (0.0) |
| Parotidectomy | 1 (0.4) | 0 (0.0) |
| Peripheral edema | 1 (0.4) | 0 (0.0) |
| Peritonitis | 0 (0.0) | 1 (0.4) |
| Pharyngitis | 1 (0.4) | 1 (0.4) |
| Pneumonia | 0 (0.0) | 1 (0.4) |
| Pneumothorax | 0 (0.0) | 1 (0.4) |
| Rectal cancer | 0 (0.0) | 1 (0.4) |
| Rectal ulcer | 0 (0.0) | 1 (0.4) |
| Right bundle branch block | 0 (0.0) | 1 (0.4) |
| Sepsis | 0 (0.0) | 2 (0.8) |
| Skin eruption | 5 (2.1) | 0 (0.0) |
| Subcutaneous abscess | 0 (0.0) | 1 (0.4) |
| Subcutaneous bleeding | 1 (0.4) | 0 (0.0) |
| Subdural hemorrhage | 2 (0.8) | 0 (0.0) |
| Sudden hearing loss | 1 (0.4) | 0 (0.0) |
| Tinnitus | 1 (0.4) | 0 (0.0) |
| Transient ischemic attack | 0 (0.0) | 1 (0.4) |
| All-cause (non-cardiovascular) death | 2 (0.8) | 4 (1.6) |
| Upset | 1 (0.4) | 0 (0.0) |
| Ureter cancer | 1 (0.4) | 0 (0.0) |
| Urinary tract infection | 0 (0.0) | 1 (0.4) |
| Urinary lithiasis | 0 (0.0) | 1 (0.4) |
| Ventricular fibrillation | 0 (0.0) | 1 (0.4) |
| Ventricular premature contraction | 2 (0.8) | 1 (0.4) |
| Ventricular tachycardia | 0 (0.0) | 1 (0.4) |
| Vertigo | 2 (0.8) | 0 (0.0) |
| Xeroderma | 0 (0.0) | 1 (0.4) |

Data are shown as number (%).
