# Supplementary figures and images for: Febuxostat does not delay progression of carotid atherosclerosis in patients with asymptomatic hyperuricemia: A randomized, controlled trial
Source: PLoS Med. 2020 Apr 22;17(4):e1003095. doi: 10.1371/journal.pmed.1003095 (PMC7176100; doi:10.1371/journal.pmed.1003095)

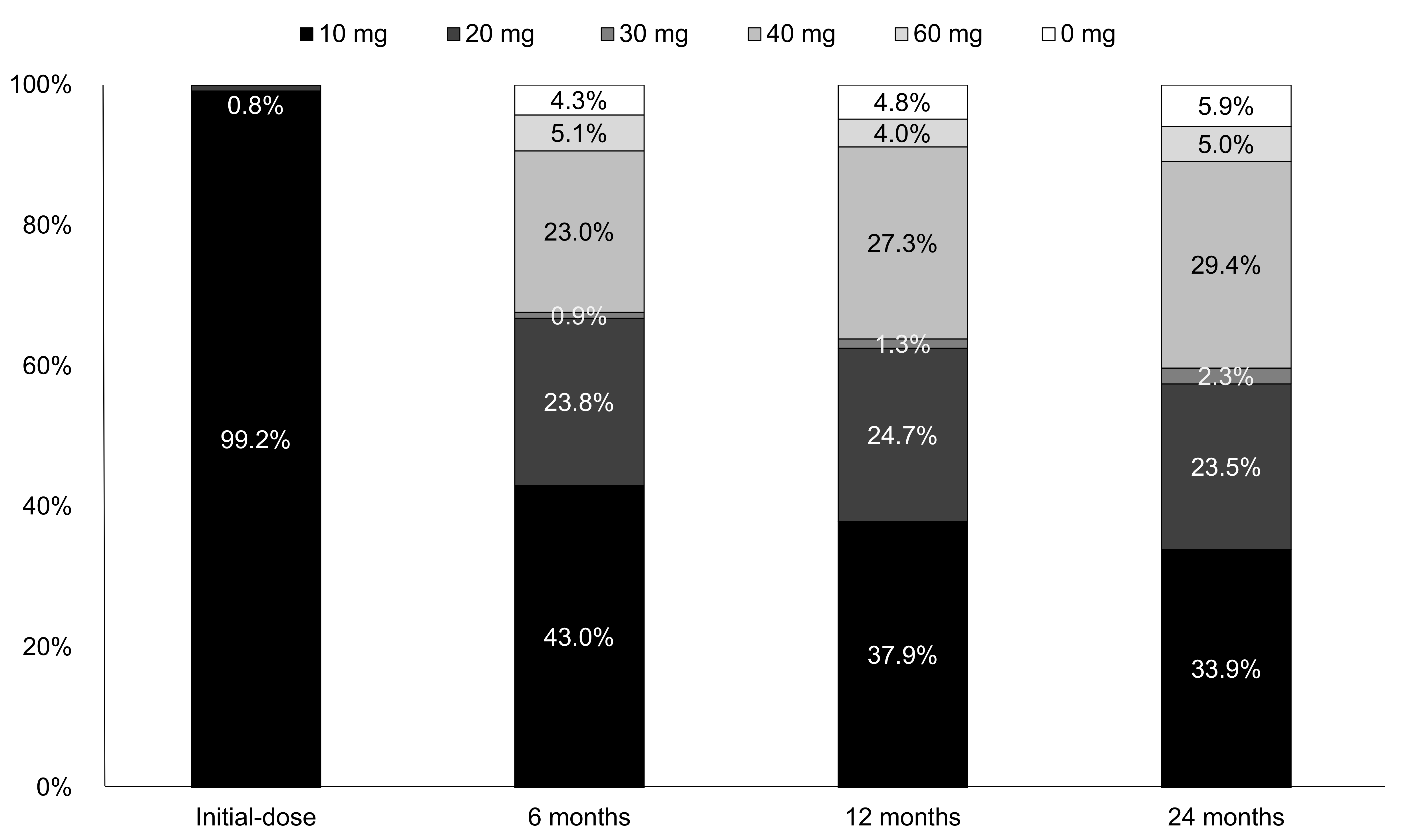

Supplement: S1 Fig — (TIF) [file pmed.1003095.s012.tif]

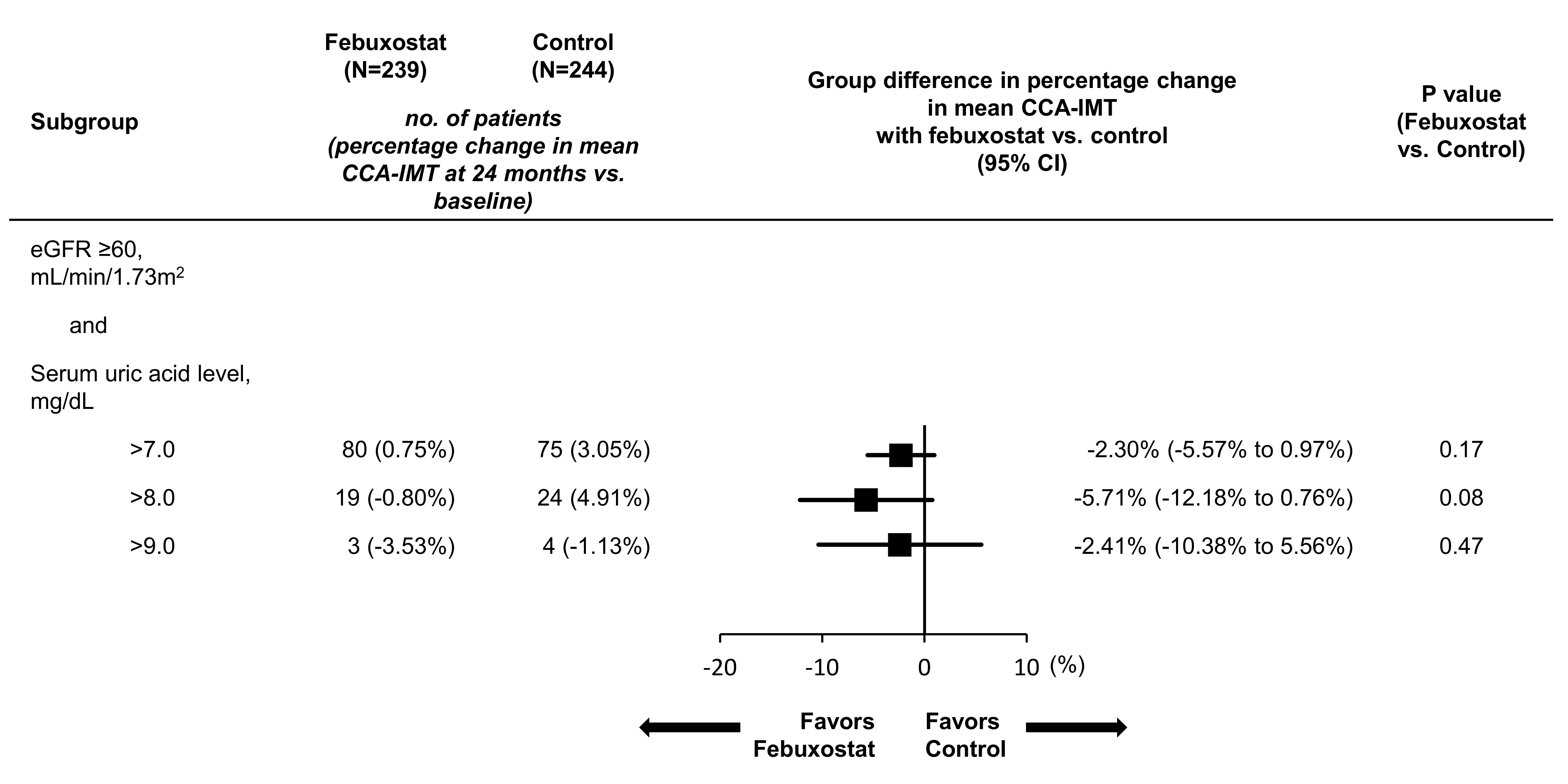

Supplement: S2 Fig — CCA, common carotid artery; eGFR, estimated glomerular filtration rate; IMT, intima-media thickness (TIF) [file pmed.1003095.s013.tif]
